# Photoluminescence of Ni(II), Pd(II), and Pt(II) Complexes [M(Me_2_dpb)Cl] Obtained from C‒H Activation of 1,5-Di(2-pyridyl)-2,4-dimethylbenzene (Me_2_dpbH)

**DOI:** 10.3390/molecules26165051

**Published:** 2021-08-20

**Authors:** Lukas Kletsch, Rose Jordan, Alicia S. Köcher, Stefan Buss, Cristian A. Strassert, Axel Klein

**Affiliations:** 1 Department für Chemie, Institut für Anorganische Chemie, Universität zu Köln, Greinstraße 6, D-50939 Köln, Germany; lukas.kletsch@uni-koeln.de (L.K.); rjordan@smail.uni-koeln.de (R.J.); akoecher@smail.uni-koeln.de (A.S.K.); 2 Institut für Anorganische und Analytische Chemie, Westfälische Wilhelms-Universität Münster, Corrensstr. 28/30, D-48149 Münster, Germany; s_buss14@uni-muenster.de; 3 CeNTech, CiMIC, SoN, Westfälische Wilhelms-Universität Münster, Heisenbergstr. 11, D-48149 Münster, Germany

**Keywords:** nickel, palladium, platinum, cyclometalation, C‒H activation, photoluminescence

## Abstract

The three complexes [M(Me_2_dpb)Cl] (M = Ni, Pd, Pt) containing the tridentate *N*,*C*,*N*-cyclometalating 3,5-dimethyl-1,5-dipyridyl-phenide ligand (Me_2_dpb^−^) were synthesised using a base-assisted C‒H activation method. Oxidation potentials from cyclic voltammetry increased along the series Pt < Ni < Pd from 0.15 to 0.74 V. DFT calculations confirmed the essentially ligand-centred π*-type character of the lowest unoccupied molecular orbital (LUMO) for all three complexes in agreement with the invariant reduction processes. For the highest occupied molecular orbitals (HOMO), contributions from metal d_yz_, phenyl C4, C2, C1, and C6, and Cl p_z_ orbitals were found. As expected, the d_z_^2^ (HOMO-1 for Ni) is stabilised for the Pd and Pt derivatives, while the antibonding d_x_^2^_−y_^2^ orbital is de-stabilised for Pt and Pd compared with Ni. The long-wavelength UV-vis absorption band energies increase along the series Ni < Pt < Pd. The lowest-energy TD-DFT-calculated state for the Ni complex has a pronounced d_z_^2^-type contribution to the overall metal-to-ligand charge transfer (MLCT) character. For Pt and Pd, the d_z_^2^ orbital is energetically not available and a strongly mixed Cl-to-π*/phenyl-to-π*/M(d_yz_)-to-π* (XLCT/ILCT/MLCT) character is found. The complex [Pd(Me_2_dpb)Cl] showed a structured emission band in a frozen glassy matrix at 77 K, peaking at 468 nm with a quantum yield of almost unity as observed for the previously reported Pt derivative. No emission was observed from the Ni complex at 77 or 298 K. The TD-DFT-calculated states using the TPSSh functional were in excellent agreement with the observed absorption energies and also clearly assessed the nature of the so-called “dark”, i.e., d‒d*, excited configurations to lie low for the Ni complex (≥3.18 eV), promoting rapid radiationless relaxation. For the Pd(II) and Pt(II) derivatives, the “dark” states are markedly higher in energy with ≥4.41 eV (Pd) and ≥4.86 eV (Pt), which is in perfect agreement with the similar photophysical behaviour of the two complexes at low temperatures.

## 1. Introduction

Luminescent transition metal complexes have gained enormous importance in the last 20 years with potential applications in fields such as photocatalysis [1,2], sensing [3,4,5,6], and optoelectronic devices [5,6,7,8,9,10,11,12,13,14]. Phosphorescent metal complexes are of particular interest in the field of OLED (organic light emitting diode) applications, as these materials are able to harvest all generated excitons in the operating electroluminescent devices [11]. Efficient intersystem crossing (ISC) and otherwise spin-forbidden phosphorescence is favoured through large spin-orbit coupling (SOC) of the heavy metal centres [5,6,7,8,9,10,11]. Typical phosphorescent emitters are based on metal cations such as the d^6^-configured Re(I), Ru(II), Os(II), and Ir(III) centres [3,4,8,9,10,11,12], or the d^8^-configured Pt(II) or Au(III) species [3,4,5,6,7,9,10,11,12,13,14,15,16]. The d^8^ electron configured metals adopt square-planar geometries with open coordination vacancies in the axial positions. These axial flanks can lead to metal–metal (M···M) and/or π···π stacking interactions in aggregates with red-shifted emissions from MMLCT states (metal–metal-to-ligand charge transfer character, eventually with excimeric M···M shortening), along with metal-perturbed ligand-centred (π‒π*) excited configurations of the monomeric species [17,18,19,20,21,22,23,24,25,26].

While phosphorescent Pt(II) complexes are common in the literature, luminescent materials based on the lighter group 10 metals, namely Pd(II) [13,23,24,25,26,27,28,29,30,31,32] and Ni(II), [33] are much more scarce. This is attributed to the less efficient SOC associated with the lighter metal ion, as well as to the lower ligand field splitting of the metal d-orbitals providing thermally accessible metal-centred (MC or d‒d*) states with dissociative character, which enable an efficient non-radiative decay pathway back to the ground state via conical intersections [31,34,35,36].

**Scheme 1:**
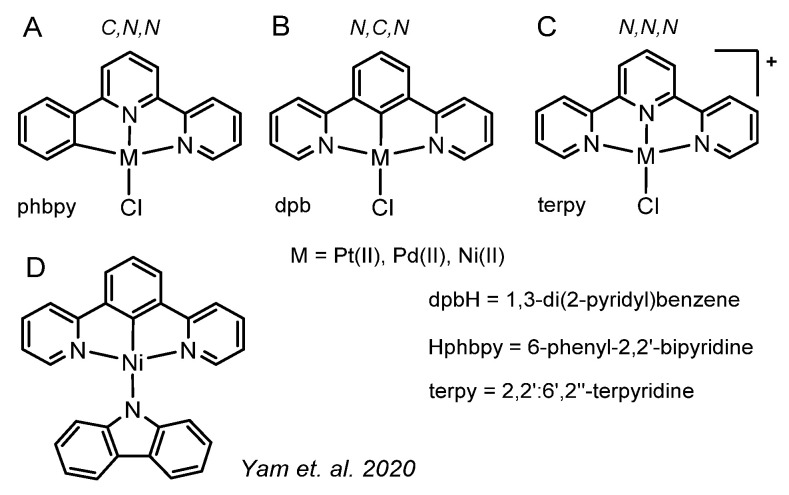
Neutral Pt(II), Pd(II), and Ni(II) complexes with cyclometalating *C*,*N*,*N* (**A**) and *N*,*C*,*N* (**B**) coordinating ligands phbpy and dpb compared with the analogous cationic complexes with the *N*,*N*,*N* coordinating terpy (**C**). [Ni(dpb)(carbazolate)] (**D**) showed triplet emission at 77 K in frozen glassy matrices and at 298 K in the solid state [33].

Cyclometalating tridentate or tetradentate ligands revealed to be very suitable to provide a stronger ligand field and move up the so-called “dark” (non-radiative) d‒d* states in energy above the triplet-emitting metal-to-ligand charge transfer (MLCT) or π-π* states (or mixtures thereof) [5,6,7,13,16,17,26,27,28,29,30,32,33,37,38,39,40,41,42,43,44]. Amongst them, tridentate *C*,*N*,*N* or *N*,*C*,*N* coordinating units (Scheme 1A,B) derived from the prototypical tridentate *N,N,N*-ligand 2,2′:6′,2″-terpyridine (Scheme 1C) constitute a well-studied group [5,7,30,32,33,36,37,38,42,43,44]. Their relatively straightforward syntheses and modification by substitution combined with the benefit of a fourth highly variable ancillary ligand at the metal centre makes complexes with these ligands interesting candidates for larger-scale studies. Very recently, a benchmarking study reported on the Ni(II) complex [Ni(dpb)(carbazolate)] (dpbH = 1,3-di(2-pyridyl)benzene (Scheme 1D) which showed triplet emission at 77 K in frozen glassy matrices and at 298 K in the solid state [33].

Being interested in comparative studies of isoleptic Pt(II), Pd(II), and Ni(II) complexes [30,32,45,46], we started to explore the three complexes bearing the dpb ligand. Very recently, we found an elegant way to cyclonickelate the dpb ligand forming [Ni(dpb)Cl] [47]. This complex, the precursor for the benchmarking [Ni(dpb)(carbazolate)], was so far only accessible through a transmetalation procedure using the organomercurial [Hg(dpb)Cl] and NiCl_2_ [33]. We recently synthesised this key complex in 76% yield using anhydrous NiCl_2_ and dpbH in a base-assisted direct metalation in refluxing xylene using the base combination K_2_CO_3_/KOAc. This reaction was initially developed for the *C*,*N*,*N* coordinating ligand 6-phenyl-2,2′-bipyridine (Hphbpy; Scheme 1A) [48].

**Scheme 2:**
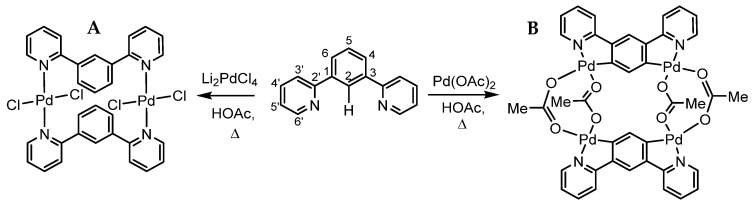
Cyclometalation reaction of dpbH with Li_2_[PdCl_4_] (**A**) and Pd(OAc)_2_ (**B**). Adopted from Cardenas, D.J.; Echavarren, A.M.; and Ramirez de Arellano, M.C. in Divergent Behavior of Palladium(II) and Platinum(II) in the Metalation of 1,3-Di(2-pyridyl)benzene, *Organometallics* **1999**, *18*, 3337–3341, Reference [49].

Compared with the Ni derivative, the Pt(II) complex [Pt(dpb)Cl] is easily accessible via the established electrophilic C‒H activation of the protoligand dpbH with K_2_[PtCl_4_] in boiling glacial acetic acid (HOAc) and yields of 50 to 80% were reported [38,49,50,51,52,53,54]. For the Pd derivative [Pd(dpb)Cl], the same method using Li_2_[PdCl_4_] led to the non-cyclometalated binuclear complex [Pd_2_Cl_2_(µ-κ^2^*N*,*N*dpbH)] (Scheme 2A), whereas the use of Pd(OAc)_2_ gave the cyclometalated tetranuclear [Pd_4_(µ-κ^2^,κ^2^dpb)_2_(µ-κ^1^,κ^1^-OAc)_4_] species (Scheme 2B) [49]. Based on these early findings, the above mentioned transmetalation procedure using [Hg(dpbCl] was used in a later study [55]. The Hg-transmetalation method was initially developed for the *C*,*N*,*N* coordinated derivative [Pd(phbpy)Cl] [56]. Later, the same authors reported the synthesis of [Pd(dpb)Cl] and a chiral derivative from Na_2_[PdCl_4_] and dpbH in refluxing HOAc with an 87% yield [51]. However, a successful repetition of this procedure was never reported, although the complex [Pd(dpb)Cl] and its derivatives have been described as promising candidates for Pd-based triplet luminescence or sensitisation [35,36]. In a very recent report, the 6′-(4-OMe)Ph-substituted derivative [Pd(MeOPh_2_dpb)Cl] was synthesised from K_2_[PdCl_4_] and the protoligand in NaHCO_3_ HOAc/H_2_O showed a 42% yield, whereas for the derivative, in which the two methoxy groups were replaced by an intramolecular ether-connection, a 66% yield was achieved [57].

**Scheme 3:**
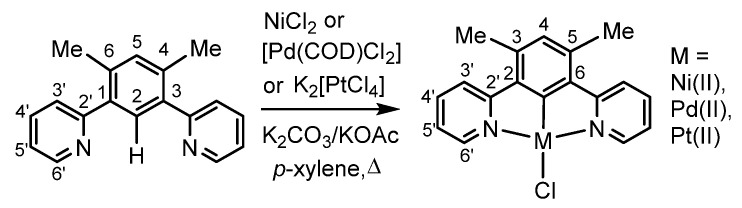
Synthesis of the target [M(Me_2_dpb)Cl] complexes from the present study; note the different numbering of the Me_2_dpbH protoligand and complexes.

When trying to apply the base-assisted direct cyclometalation of dpbH with Pd(II) precursors such as K_2_[PdCl_4_], PdCl_2_, or [Pd(COD)Cl_2_] (COD = 1,5-cyclooctadiene), we were not able to obtain the complex [Pd(dpb)Cl]. Instead, we obtained mixtures of the binuclear and tetranuclear complexes A and B (Scheme 2) together with traces of the target complex and further unidentifiable by-products. In view of the “wrong” cyclometalation site at the 4,6-positions of the dpbH ligand, as shown in the structure F, we decided to use the 4,6-dimethylated dpbH derivative Me_2_dpbH (Scheme 3).

Thus, for the herein reported study, we used the Me_2_dpbH protoligand and present the syntheses and characterisation of the three cyclometalated Ni(II), Pd(II), and Pt(II) complexes [M(Me_2_dpb)Cl] (Scheme 3) containing the tridentate anionic 3,5-dimethyl-2,6-dipyridyl-phenide ligand (Me_2_dpb^−^). The complexes were synthesised using a base-assisted C‒H activation method starting from the protoligand 1,3-di(2-pyridyl)-4,6-dimethylbenzene (Me_2_dpbH), the suitable metal precursors, and a 1:1 mixture of KOAc and K_2_CO_3_ in vigorously dried aprotic solvents. This allowed to study the homologous Ni, Pd, and Pt series of these complexes concerning their fundamental electronic properties through electrochemical methods, UV-vis absorption, and time-resolved photoluminescence spectroscopy, which were interpreted with the aid of (TD-)DFT calculations.

Furthermore, our study allowed us to trace the influence of the two methyl groups of the dpb ligand system. Starting from the parent [Pt(dpb)Cl] systems, various substituents on both ligand parts have been introduced previously and the effects on UV-vis absorption, photoluminescence, and electrochemical potentials were studied [5,16,38,52,54,58,59,60,61,62,63,64,65]. Within this series, [Pt(Me_2_dpb)Cl] was very recently synthesised from the protoligand Me_2_dpbH and K_2_[PtCl_4_] in a 50% yield using the “classical” procedure of heating in glacial acetic acid [60]. The electrochemical and photophysical properties of this complex were compared with the un-substituted complex [Pt(dpb)Cl] (dpbH = 1,3-di(2-pyridyl)-benzene) and 1,2,3-triazole-containing derivatives [60]. Thus, the present study also completes the opto-electronic dataset for this complex.

## 2. Results and Discussion

### 2.1. Preparation and Analytical Characterisation

The three complexes [M(Me_2_dpb)Cl] were synthesised from the protoligand 1,5-di(2-pyridyl)-2,4-dimethylbenzene (Me_2_dpbH), anhydrous NiCl_2_, [Pd(COD)Cl_2_], or K_2_[PtCl_4_] by using the base-assisted C‒H activation method with KOAc and K_2_CO_3_ in vigorously dried aprotic solvents, as previously reported for the Ni(II) complex [Ni(dpb)Cl] (dpbH = 1,3-di(2-pyridyl)-benzene) [47] and the *C*,*N*,*N* coordinated derivative [Ni(phbpy)Cl] (Hphbpy = 6-phenyl-2,2′-bipyridine) [48]. The three products were characterised through elemental analyses, EI-MS(+), ^1^H, and ^13^C NMR spectroscopy, and additionally ^195^Pt NMR for [Pt(Me_2_dpb)Cl] (see Supplementary Materials). The ^1^H and ^13^C NMR of the Pt(II) complex matched perfectly the previously reported data [60].

The Pt derivative [Pt(Me_2_dpb)Cl] was previously synthesised via the classical method of heating the ligand and K_2_[PtCl_4_] in glacial acetic acid [38,50,52,53,54] for about 1 h with a yield of 50% [60]. With a 70% yield, our method is superior, while the reaction time was markedly longer.

Using the base-assisted C‒H activation method with KOAc and K_2_CO_3_ in *p*-xylene, the yield for the Pd derivative was increased from 6% to 68% by avoiding light. Importantly, this procedure replaces the previously applied transmetalation using organomercurials [55] and we are optimistic to be able to increase the yield even more through optimisation of the reaction conditions. When applying the same procedure for the unsubstituted protoligand dpbH with Pd, we obtained the aforementioned and previously reported tetrameric complex [Pd_4_(µ-κ^2^,κ^2^-dpb)_2_(µ-κ^1^,κ^1^-OAc)_4_] with yields of up to 31% [49]. When using pivalic acid (HOPiv) instead of HOAc, we obtained the complex [Pd_4_(µ-κ^2^,κ^2^-dpb)_2_(µ-κ^1^,κ^1^-OPiv)_4_] with a 28% yield (see Experimental Section and Supplementary Materials, Scheme S1). Then, we explored the synthesis of [Pd(Me_2_dpb)Cl] through reaction of the ligand with Na_2_[PdCl_4_] in acetic acid, as reported for the unsubstituted derivative [Pd(dpb)Cl] (87%) [52], and obtained the target complex in a 98% yield.

The yield of 94% for the Ni complex [Ni(Me_2_dpb)Cl] is excellent and markedly exceeded the yield for the unsubstituted derivative [Ni(dpb)Cl], reaching 76% [47].

To summarise the synthesis experiments, the base-assisted metalation works for all three elements but with very different yields of 94% for Ni and about 70% for Pt and Pd. While for Ni our method is without an alternative, the yields for the Pt complex were comparable to those of other methods. For the Pd complex, the classical electrophilic substitution—i.e. heating the components in acetic acid—gave an excellent yield of 98%, which so far exceeds our base-assisted metalation method.

### 2.2. X-ray Diffractometric Analysis of Single Crystals and DFT-Calculated Structures

From the Pt, Pd, and Ni complexes, single crystals were obtained for X-ray diffraction experiments. For Ni, the compound [Ni(Me_2_dpb)Cl]·CH_2_Cl_2_ was solved and refined in an orthorhombic cell in the space group *P*bca (Table S1, Figures S1–S3). Unfortunately, the overall quality of this structure is low, which is due to the poor characteristics of the crystals (*R*_int_~24%). The molecular metrics were refined to reasonable values and the agreement with the DFT-calculated data was good (Table S2). [Pd(Me_2_dpb)Cl] crystallised without solvent molecules and the structure was solved in monoclinic *P*21/c with excellent refinement parameters (Table S1, Figures S4–S6). For Pt, we obtained the previously reported triclinic structure (*P*-1) for the compound [Pt(Me_2_dpb)Cl]·CH_2_Cl_2_ [60].

**Figure 1:**
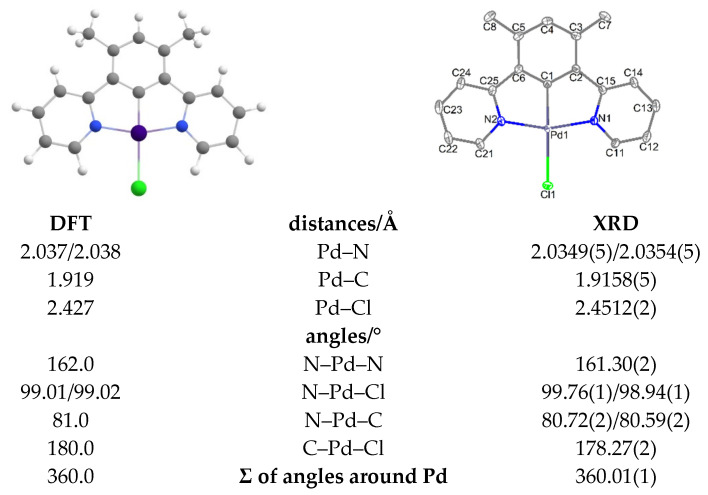
DFT-calculated molecular structure of [Pd(Me_2_dpb)Cl] (left) and experimental structure from single crystal X-ray diffraction (ORTEP plot with displacement ellipsoids at 50% probability, right) (Table S3).

The experimental data for the Pd complex [Pd(Me_2_dpb)Cl] and the previously reported data for the Pt complex [Pt(Me_2_dpb)Cl]·CH_2_Cl_2_ were used to benchmark our DFT geometry-optimisation (BP86/def-TZVP/D3/COSMO(THF)) on the reported data (Figure 1) with a very good agreement between the calculated molecular metrics and reported values (Tables S3 and S4).

### 2.3. Electrochemistry and DFT Calculations of Frontier Orbitals

**Figure 2:**
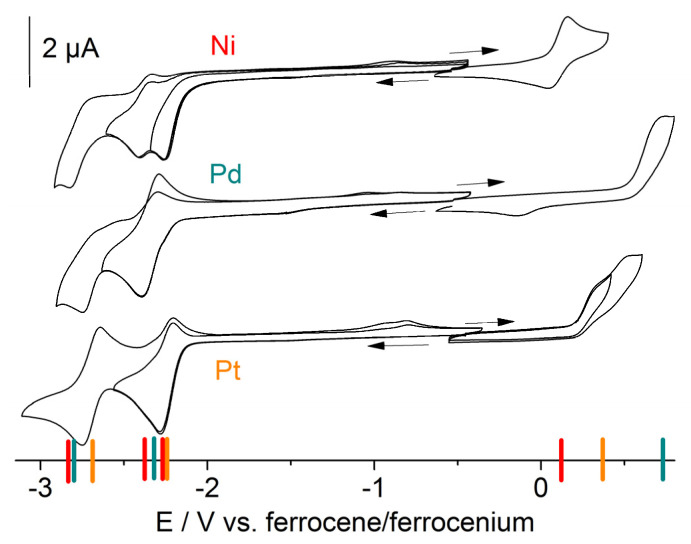
Cyclic voltammograms of [M(Me_2_dpb)Cl] in *n*-Bu_4_NPF_6_/THF. Coloured bars represent half-wave potentials (*E*_1/2_) for reversible reductions and anodic peak potentials (*E*_pa_) for irreversible oxidations.

At first glance, the electrochemical behaviour of the three complexes [M(Me_2_dpb)Cl] is very different. The cyclic voltammograms (CVs) of the Pt and Pd complexes exhibit reversible first reduction processes and for the Pt derivative, even a second reversible process is found (Figure 2). For the Ni derivative, a completely irreversible first reduction is followed by a partially reversible second process and a third irreversible wave. The same features were found for the unsubstituted Ni complex [Ni(dpb)Cl] and ascribed to rapid cleavage of the halide Cl^−^ after reduction [47]. Such an EC (electrochemical reduction + chemical reaction) mechanism was previously studied in detail for the *C*,*N*,*N* coordinated complex [Ni(phbpy)Br] [66,67]. The reversible behaviour of the Pd and Pt complexes [M(Me_2_dpb)Cl] means that the Cl^−^ cleavage is not observed on the timescale of the CV experiment. A reversible behaviour was also previously reported for derivatives of [Pt(Rdpb)Cl] (HRdpy = substituted 1,5-di(2-pyridyl)-benzene) [50,60]. The two observed reduction potentials for the Pt and Pd complex differ by quite constant values (0.41 and 0.45 V; Δ*E* (Red1‒Red2), Table 1), which stands in contrast to the *C*,*N*,*N* coordinated complexes [M(phbpy)Cl], for which the separation is markedly larger (~0.68 V). This and the overall lower reduction potentials is in agreement with the superior accepting properties of the phbpy ligand that contains a 2,2′-bipyridine unit, in contrast to the two phenyl-separated pyridyl units of the dpb ligand [47], and is a clear indication of dpb-based reduction processes for all three complexes. In addition, for the oxidation processes, the Ni derivative (reversible wave) differs markedly from the Pt and Pd (both irreversible) species.

The first reduction potentials at around −2.3 V are similar for all three complexes with only slightly lower values for the Pd derivative (Table 1). In contrast to this, the oxidation processes are very different for the three metals following the series Ni < Pt < Pd. In recent studies on such homologous series including the series [M(phbpy)(X)] (X = Cl or CN), the oxidation potentials increased along the series Ni < Pt < Pd [32,46]. However, ultraviolet photoelectron spectroscopy (UPS) on a series of the three metals chelated with an *O*,*N*,*N*,*S*-coordinating phenolate-pyridine thiosemicarbazone, the observed ionisation energy ranked as Pt < Ni < Pd [46].

DFT-based single point calculations were performed on the optimised geometries using the hybrid functional TPSSh [68], which has recently been shown to provide qualitatively very good results for organometallic Ni and also for Pd and Pt complexes [46,47,69]. The calculations show an essentially ligand-centred π*-type lowest unoccupied molecular orbital (LUMO) with very similar energies for all three complexes at around −2.3 eV (Figure S8), in agreement with the invariant experimental reduction potentials (Figure 2). The largest contribution comes from the two pyridyl units, while C1, C2, and C6 from the central phenyl core have only a minor impact. For the second LUMO (i.e., LUMO+1), the phenyl C2, C3, C5, and C6 show marked contributions. This is in line with rather invariant reduction potentials of the Pt(II) complexes of substituted dpb ligands (Table 1). 3,5-Me_2_-substitution leads to slightly lower (more negative) potentials, while 4-Me substitution has no impact [50,54]. Slightly higher (less negative) reduction potentials were obtained for F- or CF_3_-substituted derivatives (Table 1) with the highest reported shift of +0.26 V compared with the parent Pt complex when introducing the 3,5-(CF_3_)_2_-dpb ligand [54]. Metal contributions of the d_x_^2^−_y_^2^-type orbital to the unoccupied MOs are found lowest in energy for Ni (LUMO+3) and markedly higher for both Pd (LUMO+4) and Pt (LUMO+5), as expected from the drastically increased ligand field across the series Ni < Pd < Pt.

The highest occupied molecular orbital (HOMO) gains essential contributions from phenyl C4, C2, C1, and C6, as well as metal d_yz_ and Cl p_z_ orbitals (the *y*-axis bisects the molecule and z stands perpendicular to the coordination plane, Figure S8). Although their character is similar for the three metals, the energies are quite different, decreasing from Ni > Pt > Pd and thus excellently reproducing the series of oxidation potentials, which confirms the versatility of the TPSSh functional for the entire triad [46,47,68,69]. The calculated compositions also agree very well with the observation that the methyl substitution in the 3,5-positions (dpb→Me_2_dpb) does not have a marked effect on the oxidation potential, while the 4-methyl or 4-methylester substitution strongly modifies the oxidation potential. An orbital with d_z_^2^ character forms the HOMO-1 orbital for Ni; for Pd, an orbital of the d_xy_-type lies at the same energy; and for Pt, the d_z_^2^ orbital is markedly lower in energy than the d_xy_-type, again properly reproducing the trends in ligand field splitting within the triad. These differences might also account for the variations in reversibility for the oxidation. Experimentally, the oxidation potentials are quite invariant upon substitution of the parent [Pt(dpb)Cl] complex, as outlined above, but a marked solvent effect is found (Table 1) in agreement with the oxidation locus extended over the phenyl‒M‒Cl unit.

The calculated HOMO-LUMO gaps increase along the series Ni < Pt < Pd and thus qualitatively agree well with those derived from the electrochemical measurements (Δ*E* (ox1‒red1) (Table 1).

### 2.4. UV-Vis Absorption Spectroscopy and TD-DFT Calculated Transitions

**Figure 3:**
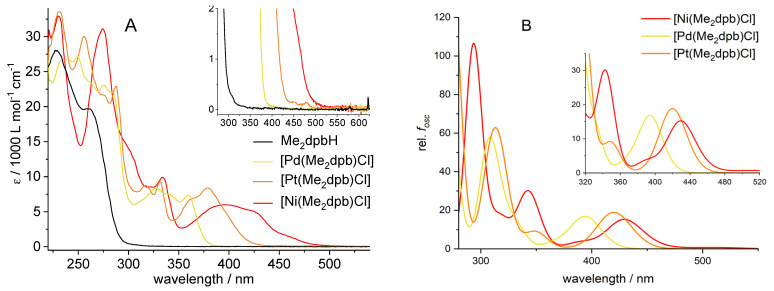
UV-vis absorption spectra of Me_2_dpbH and the complexes [M(Me_2_dpb)Cl] in CH_2_Cl_2_ solution (**A**) and TD-DFT-calculated spectra (**B**) (TPSSh/def2-TZVP level of theory). The yellow (Pd), orange (Pt), and red (Ni) colour code refer to the colours of the compounds.

The UV-vis absorption spectra of the three complexes in the CH_2_Cl_2_ solution (Figure 3, data in Table 2) are characterised by very intense bands in the UV-range of up to 300 nm. Since they also occur for the protoligand Me_2_dpbH, we can assign them to transitions into π-π* states. They are followed by intense, structured absorptions in the range of 300 to 450 nm. For the Ni complex, the long-wavelength absorption band shows a maximum at 395 nm, an intense shoulder at 420 nm, a small component at 465 nm, and finally tails down to a cut-off at 516 nm (Figure 3A insert). For the Pt derivative, the maximum of this band is blue-shifted to 380 nm and the cut-off appears at 442 nm. For the Pd complex, the maximum is even more blue-shifted to 360 nm and the cut-off shifts to 402 nm. Thus, there is a clear series of increasing optical gaps, i.e., Ni (2.40) < Pt (2.80) < Pd (3.08), when taking these cut-off energies into account. They are slightly higher than the electrochemical gaps for Ni (+0.04 eV) and Pd (+0.01 eV), but markedly different for Pt (+0.21 eV). Basically, these differences represent the reorganisation after the oxidation or reduction compared with the vertical Franck–Condon excitation.

A closer look reveals some low-energy features with very low intensities at 450 and 480 nm for the Pt complex (Figure 3A, insert, and Table 2, *λ*_7_). They are ascribed to the spin-forbidden transitions into the triplet manifold. They partly gain allowance due to the large spin-orbit coupling (SOC) of Pt [11,38,63] and have been previously reported for [Pt(Me_2_dpb)Cl] [60] and the complex [Pt(dpb)Cl] with the unsubstituted ligand [50]. The absorption spectra recorded for [Pt(Me_2_dpb)Cl] fully agrees with the previously reported data [60].

When comparing Pt complexes with substituted dpb ligands, rather small variations were found for the dominant band maximum at around 380 nm (Table 2, *λ*_5_); e.g., for the 3,5-difluorinated derivative [Pt(3,5-F_2_-dpb)Cl], this band is blue-shifted by only 350 cm^−1^ from 380 nm to 375 nm if compared with the Me_2_dpb or dpb complex [58]. However, 3,5-substitution seems to shift the long-wavelength component at around 400 nm (Table 2, *λ*_6_), which is visible for the parent dpb complex, the 4-Me and 4-MeOOC [50] derivatives, and also for the 3-F complex [58] (Table 2). Remarkably, substitution with electronegative groups as in 3,4,5-F or 3,5-CF_3_ seems to be detrimental for a blue-shift of the 380 nm band and the absorption maxima of these two complexes lie very close to the parent complex [54].

**Figure 4:**
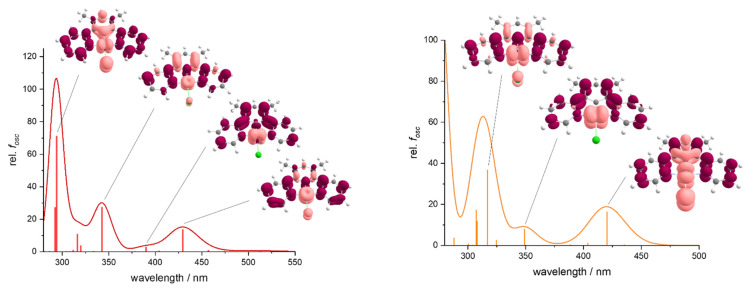
TD-DFT-calculated transitions for [M(Me_2_dpb)Cl] with M = Pt (**left**) and Ni (**right**), calculated at the TPSSh/def2-TZVP level of theory.

TD-DFT calculated transitions generally agree qualitatively well with experimentally observed absorption maxima (Figure 4 and Figure S9, Tables S6–S8) with the calculated low-energy transitions at 429 nm (Ni), 420 nm (Pt), and 394 nm (Pd), but slightly red-shifted if compared with the experimental maxima (395, 380, 360 nm). These bands have approximately the same character for the Pt and Pd derivatives and are best described as transitions into Cl(p)‒M(d_yz_)‒ph to ligand π*(py) charge transfer states with mixed XLCT/MLCT/ILCT character (Figure 4, left). The calculated vertical S_0_→S_1_ transitions (Tables S6 and S7) show that these bands essentially mirror the excitation into HOMO→LUMO+1 configurations, while the HOMO→LUMO configured states were calculated with energies corresponding to 435 nm for Pt and to 403 nm for Pd but with very low intensities. However, they match very well with the so-called optical cut-offs for these two complexes at 442 nm (Pt) and 402 nm (Pd). Moreover, a look at the calculated frontier orbitals of the ground states shows that an overlap of the HOMO with the LUMO+1 looks more probable than with the LUMO (Figure S8). Both findings strongly support our calculations and assignments.

For the Ni complex, the lowest-energy transition has markedly lower Cl-character and the contributions of the phenyl group are different from those of the Pd and Pt derivatives, and they are also generally smaller (Figure 4). Thus, for the Ni complex, we can relate the long-wavelength transition to an excitation into a state with almost pure MLCT character (d_z_^2^ to π*). The calculated vertical S_0_→S_1_ transitions (Table S8) confirm this and show that the 429 nm band corresponds to an *S*_1_ state with almost equal HOMO-2→LUMO (52%) and HOMO→LUMO+1 (43%) contributions. The HOMO-1→LUMO configuration is calculated with an energy of 513 nm and matches perfectly the optical cut-off observed at 516 nm.

The different character of the states involved in the long-wavelength bands for the Ni complex and for the heavier homologues is due to the energetic availability of the Ni d_z_^2^ orbital, while for the Pd and Pt derivatives, this orbital is markedly stabilised in relation to the calculated energies and composition of the *S*_0_ ground state (Figure S8).

The shoulder calculated at 390 nm for the Ni complex corresponds to a transition into a state with high MLCT (d_xz_ to π*; HOMO-1→LUMO+1, 62%) character with the π* extending over the pyridyl and phenyl cores, but it also obtains a marked contribution from the Ni d_x_^2^_−y_^2^ (HOMO-1→LUMO+3 configuration, 16%). For the Pd and Pt derivatives, this kind of transition is blue-shifted to 349 nm for Pt and 331 nm for Pd and does not contain any contribution from d_x_^2^_−y_^2^ orbitals to the excited state configuration. For Pd, the lowest energy state with such d_x_^2^_−y_^2^ component is reached at 281 nm (HOMO-2→LUMO+4, 71%), while for Pt, a band at 255 nm involves such states (HOMO-2→LUMO+5, 38%). This perfectly reflects the strongly increased ligand field splitting for Pd(II) and Pt(II) compared with Ni(II) and is in line with the efficient photoluminescence for the Pt and Pd complexes, while for Ni, the low-lying excited d‒d* configuration very probably quenches the luminescence through radiationless relaxation (see later). In contrast to this, the 343 nm band of the Ni complex is related to a state with mixed ILCT/MLCT character, which is unmatched by Pt and Pd.

The most intense bands in the spectra around 300 nm differ markedly for all three complexes with respect to the excited state character. For the Pt derivative, a mixed ILCT/MLCT contribution was observed with a strong Pt(d_xz_) component and a rather low Cl(p_z_) participation. For the Pd complex, the Cl(p_z_) contribution is increased while the participation of the phenyl core is almost vanished, and an overall mixed XLCT/MLCT character must be ascribed (Figure S9). For the Ni derivative, the phenyl contribution becomes strongest within this series and is very similar to the character of the state reached by long-wavelength transitions, where a mixed XLCT, ILCT, and MLCT character is found involving metal d_xz_ contribution.

### 2.5. Spectroelectrochemistry

**Figure 5:**
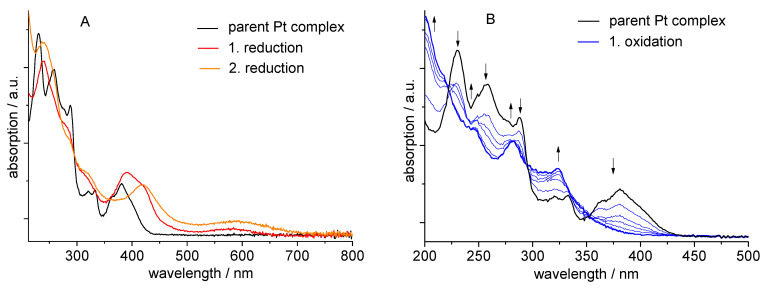
UV-vis absorption spectra of [Pt(Me_2_dpb)Cl] in THF/*n*-Bu_4_NPF_6_, recorded during cathodic reduction (**A**) and anodic oxidation (**B**).

Upon electrochemical reduction, all the three complexes [M(Me_2_dpb)Cl] exhibit long-wavelength absorptions at around 600 and 400 nm (Figure 5, Figures S10 and S11). We ascribe them to the radical anionic complexes [M(Me_2_dpb)Cl]^•−^ or [M(Me_2_dpb)(THF)]^•^ when assuming the cleavage of the Cl^−^ ligand after reduction. The bands can be assigned to transitions into π*‒π* states within the reduced dpb ligand frame and they are very similar to those observed for [Ni(dpb)Cl] [47]. The subtle red-shift of these two bands upon the second reduction for the Pt complex (Figure 5) confirms this assignment. Upon oxidation, the long-wavelength MLCT bands vanish. As in all three cases, the oxidations are irreversible in the CV experiment and the species produced upon oxidation are not clear. However, in all three cases, very similar spectroscopic features are observed (Table S9) and pointing to comparable products from oxidation and subsequent chemical reaction (decomposition).

### 2.6. Photoluminescence Spectroscopy

**Figure 6:**
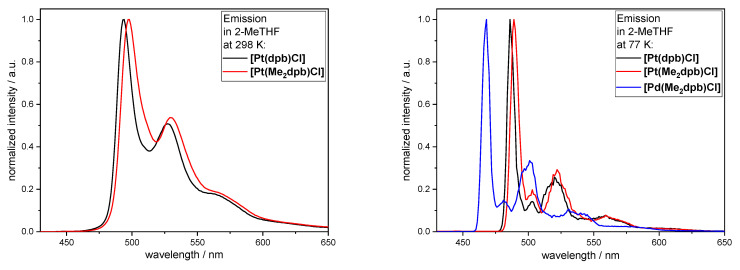
Photoluminescence spectra of [Pt(dpb)Cl] (black), [Pt(Me_2_dpb)Cl] (red), and [Pd(Me_2_dpb)Cl] (blue) in 2-MeTHF at 298 K (**left**) and at 77 K as a glassy matrix (**right**). The Pd complex was not emissive at 298 K.

We studied the photoluminescence of the three complexes [M(Me_2_dpb)Cl] (M = Pt, Pd, and Ni) alongside with the unsubstituted [Pt(dpb)Cl] at 298 K in fluid solutions (DCM and 2-MeTHF) and at 77 K in frozen glassy 2-MeTHF matrices. Under none of these conditions did the Ni complex show any photoluminescence (PL), which is consistent with the relatively low-lying d‒d * states calculated for Ni (390 nm or 3.18 eV) compared with Pd (281 nm or 4.41 eV) and Pt (255 nm or 4.86 eV). The photophysical properties are summarised in Figure 6 and Table 3, and the full set of spectra and photoluminescence decays is shown in Figures S12–S25.

The recorded data for the complexes [Pt(Me_2_dpb)Cl] [60] and [Pt(dpb)Cl] [50,54,58,63,72] agreed very well with previous reports. Our results also confirm that the 3,5-dimethyl substitution does not markedly vary the photophysical properties of the parent [Pt(dpb)Cl] complex both at 298 K and at 77 K [60]. At 298 K, only a slight red-shift of about 250 cm^−1^ is found for the 3,5-Me_2_ derivative, which vanishes at 77 K (Table 3). For the 4-Me derivative, a moderate red-shift of 565 cm^−1^ of the emission bands was previously reported at 298 K [50]. Moderate to marked blue-shifts had been found for the complexes involving the 4-MeOOC (423 cm^−1^) [50], 3-F (424 cm^−1^), and 3,5-F_2_ (947 cm^−1^) [54,58] substitution arrangements. As observed for the absorption energies, the 3,4,5-F_3_ or 3,5-(CF_3_)_2_ substitution pattern led to emission energies close to those of the parent complex [54]. Regardless of these shifts, neither the photoluminescence quantum yields (*Φ*_L_) nor the excited states’ lifetimes were markedly affected, the latter lying around 6 µs in DCM (dichloromethane) and between 3.5 and 5 µs in 2-MeTHF (2-methyl-tetrahydrofuran). At 77 K in frozen glassy matrices (2-MeTHF), both Pt complexes show a *Φ*_L_ of almost unity.

The Pd complex [Pd(Me_2_dpb)Cl] did not show any PL at 298 K, but at 77 K we recorded structured excitation and emission spectra (Figure 6), which at first glance resembled to the corresponding Pt derivative. A closer inspection showed that the first emission maximum of the Pd complex is shifted to 468 nm if compared with the Pt complex (489 nm). In addition, the low-energy excitation maximum observed for Pd at 373 nm is blue-shifted if compared with the Pt complex (394 nm). The long lifetime of about 153 µs recorded for the Pd complex can be explained by the smaller SOC, which dampens all the intrinsically spin-forbidden deactivation rates from the lowest triplet state. In fact, we found two components for the decay into the grounds state with 157 µs corresponding to the main component (92%) and about 102 µs for a minor component (8%). Both of them are markedly longer-lived if compared with the analogous Pt complexes lying between 5 and 10 µs, depending on the substitution pattern of the dpb ligand.

The most remarkable finding is the photoluminescence quantum yield of almost unity for the Pd complex. For the Pt derivative and the unsubstituted complex [Pt(dpb)Cl], the same values were found, which is in agreement with previous reports [50,54,58,60,63,72]. The Pd complex thus joins the small list of highly efficient Pd(II) complexes and very probably leads the short list of isoleptic Pt(II) and Pd(II) complexes for which Pd performs as good as Pt [13,24,27,28], while contrasting with the long list of Pt(II) outperforming their Pd(II) homologues [23,25,26,29,30,31,32,36,42,44]. The main reason for the outstanding performance of the Pd complex probably lies in the rigidity of the coordination environment and the significant ligand field splitting, which prevents radiationless deactivation processes. This calls for further dynamic quantum-chemical calculations, which will be part of future work on these complexes and derivatives with alternative co-ligands.

## 3. Materials and Methods

**Instrumentation:**^1^H, ^13^C, and correlation spectra were recorded on a *Bruker* Avance II 300 MHz (^1^H: 300 MHz, ^13^C: 75 MHz), equipped with a double resonance (BBFO) 5 mm observe probe head with a z-gradient coil (Bruker, Rheinhausen, Germany). Chemical shifts were relative to TMS (^1^H, ^13^C). UV–vis absorption spectra were recorded on a *Varian* Cary 05E spectrophotometer (Varian Medical Systems, Darmstadt, Germany). Elemental analyses were conducted using a *HEKAtech* CHNS EuroEA 3000 analyzer (HEKAtech, Wegberg, Germany). EI-MS spectra in the positive mode were measured using a *Finnigan* MAT 95 mass spectrometer. Simulations were performed using ISOPRO 3.0. Electrochemical measurements were carried out in 0.1 M *n*-Bu_4_NPF_6_ solution in THF (tetrahydrofuran) using a three-electrode configuration (glassy carbon electrode, Pt counter electrode, Ag/AgCl reference) and a Metrohm Autolab PGSTAT30 or µStat400 potentiostat (Metrohm, Filderstadt, Germany). The potentials were referenced against the ferrocene/ferrocenium redox couple as an internal standard. UV-vis-spectroelectrochemical measurements (in 0.1 M *n*-Bu_4_NPF_6_/THF solution) were performed using an optically transparent thin-layer electrode (OTTLE) cell [73] at room temperature.

**Photophysical measurements:** For the steady-state and time-resolved measurements, a FluoTime 300 spectrometer from PicoQuant was used. For the photoluminescence quantum yields, a Hamamatsu Photonics absolute PL quantum yield measurement system was used (Hamamatsu Photonics Deutschland GmbH, Geldern, Germany). All cuvettes used were round quartz cuvettes and the solvent 2-methyltetrahydrofuran (2-MeTHF) was purchased from abcr (Karlsruhe, Germany) in at least 99% purity and stabilised with 150–400 ppm BHT. The used dichloromethane (DCM) was of spectroscopic grade (Uvasol^®^). Time-resolved luminescence decay curves are shown in Figures S15–S25. Further instrumental details can be found in the Supplementary Materials.

**Single crystal structure analysis by X-ray diffractometry (XRD):** The measurements were performed at 170(2) K, employing a Bruker D8 Venture including a Bruker Photon 100 CMOS detector using Mo-Kα radiation (*λ* = 0.71073 Å) (Bruker, Rheinhausen, Germany). The crystal data was collected using APEX3 v2015.5-2 [74]. The structures were solved by dual space methods using SHELXT (*Sheldrick 2015*) [75] and the refinement was carried out with SHELXL 2017, employing the full-matrix least-squares methods on *F*_0_^2^ ≥ 2σ(*F*_0_^2^) [76]. The non-hydrogen atoms were refined with anisotropic displacement parameters without any constraints. The hydrogen atoms were included by using appropriate riding models. Data of the structure solutions and refinements can be obtained for [Ni(Me_2_dpb)Cl]·CH_2_Cl_2_ (CCDC 2093819) and [Pd(Me_2_dpb)Cl]·CH_2_Cl_2_ (CCDC 2095954) free of charge at https://summary.ccdc.cam.ac.uk/structure-summary-form (accessed on 19 August 2021) or from the Cambridge Crystallographic Data Centre, 12 Union Road, Cambridge, CB2 1EZ UK (Fax: +44-1223-336-033 or e-mail: deposit@ccdc.cam.ac.uk).

**Quantum chemical calculations using density functional theory (DFT):** All calculations were performed using the ORCA 4.2.1 program package [77,78]. Chemcraft 1.8 was used for the visualisation of results [79]. The geometries of the three complexes [M(Me_2_dpb)Cl] (M = Ni, Pd, and Pt) were optimised using the BP86 functional, def2-TZVP basis sets, *Grimme*’s D3 dispersion correction and the conductor-like screening model (COSMO) parametrised for THF [80,81,82,83,84,85]. For Pd and Pt, the def2-ECPs (ecp-28 and ecp-46, respectively) were used for the core electrons [86]. Subsequent frequency calculations on all three complexes yielded no imaginary modes, thus confirming the energetically minimal nature of the optimised geometries. Based on the optimised geometries, single point and TD-DFT [87] calculations using the *Tamm–Dancoff* approximation [88] were performed using the TPSSh hybrid functional [68,89]; def2-TZVP basis sets with def2-ECPs for Pd and Pt; *Grimme*’s D3 dispersion correction; and the conductor-like screening model (COSMO) parametrised for THF. For each compound, 80 transitions were calculated. Molecular orbital energies and isosurfaces were extracted from the single point calculations.

**Materials:** 1,3-Di(pyridyl)benzene (dpbH) [47] and the complex [Pt(dpb)Cl] [49] were synthesised as previously reported.

**Synthesis of the protoligand 1,5-di(2-pyridyl)-2,4-dimethylbenzene (Me_2_dpbH):** Under inert conditions, a *Schlenk* flask was filled with 37.8 mL (60.5 mmol, 3.2 eq.) of a 1.6 M solution of *n*-butyl lithium diluted with 140 mL THF and cooled to −78 °C. A solution of 5.5 mL (56.7 mmol, 3.0 eq.) 2-bromopyridine in 20 mL THF was slowly added dropwise and stirred for 1 h. Afterwards, a suspension of 10.30 g (75.6 mmol, 4.0 eq.) ZnCl_2_ (dried overnight at 140 °C under vacuum) in 60 mL THF was added to the reaction mixture and the reaction was warmed up to ambient temperature. Then, 1.09 g (0.95 mmol, 5 mol%) of [Pd(PPh_3_)_4_] and a solution of 5.00 g (18.9 mmol, 1.0 eq.) 1,5-dibromo-2,4-dimethylbenzene in 10 mL THF were added to the solution, which was then heated under reflux for 17 h. The reaction was terminated through the addition of 3.03 g (56.7 mmol, 2.9 eq.) NH_4_Cl in 10 mL H_2_O. The solution was extracted with EtOAc, the extract was washed with H_2_O/brine, and the combined organic layers were dried over MgSO_4_. After filtration, the solvents were evaporated and the product was purified by column chromatography with a *c*-Hex:EtOAc mixture (5:1 *v*/*v*). The product was obtained as a colourless solid (4.14 g, 15.88 mmol, 84%). R_f_ = 0.137 (*c*-Hex:EtOAc = 5:1). Elemental analysis found (calculated for C_18_H_16_N_2_, M = 260.34 g mol^−1^): C, 83.07 (83.04); H, 6.13 (6.19); N, 10.66 (10.76). ^1^H NMR (300 MHz, DMSO-*d*_6_): δ = 8.67 (ddd, 2H, *J* = 4.8, 1.9, 1.0 Hz, H8,8′), 7.87 (td, 2H, *J* = 7.7, 1.9 Hz, H6,6′), 7.57 (dt, 2H, *J* = 7.9, 1.1 Hz, H5,5′), 7.46 (s, 1H, H9), 7.36 (ddd, 2H *J* = 7.6, 4.8, 1.1 Hz, H7,7′), 7.26 (s, 1H, H1), 2.38 (s, 6H, CH_3_) ppm. ^13^C NMR (75 MHz, DMSO-*d*_6_): *δ* = 158.67 (C4,4′), 149.00 (C8,8′), 137.68 (C3,3′), 136.51 (C6,6′), 135.23 (C2,2′), 133.13 (C1), 130.99 (C9), 123.92 (C5,5′), 121.88 (C7,7′),19.82 (CH_3_). EI-MS(+) *m*/*z* = 259 (100%) [M]^+^, 245 (95%) [M‒CH_3_]^+^, 182 (10%) [M‒Py]^+^, 167 (10%) [M‒Py‒CH_3_]^+^, 129 (20%) [M‒L + Cl]^+^, 102 (5%) [M‒Py-Py]^+^, 78 (5%) [Py]^+^.

**Synthesis of [Ni(Me_2_dpb)Cl]:** In an inert flask equipped with a water trap filled with molecular sieve (3 Å), anhydrous 0.168 g NiCl_2_ (1.3 mmol, 1.3 eq.), 0.100 g KOAc (1.0 mmol, 1.0 eq.), and 0.140 g K_2_CO_3_ (1.0 mmol, 1.0 eq.) were dried for 1 h in vacuum at 170 °C. Then, 0.26 g 1,5-di(2-pyridyl)-2,4-dimethylbenzene (1.0 mmol, 1.0 eq.) and dry *p*-xylene (200 mL) were added and heated under reflux for 72 h. After cooling to ambient temperature, the precipitated solid was filtered off, washed once with *p*-xylene, and the product was extracted using THF and washed with CH_2_Cl_2_ afterwards. The solvent was removed and the dark orange product was isolated. Careful crystallisation of the *p*-xylene fraction gave further product. Total yield: 0.319 g (0.94 mmol, 94%). Elemental analysis found (calculated for C_18_H_15_N_2_NiCl, M = 353.48 g mol^−1^): C, 61.20 (61.16); H, 4.36 (4.28); N, 7.96 (7.93). ^1^H NMR (300 MHz, CD_2_Cl_2_): *δ* = 8.84 (br s, 2H, H8,8′), 7.81 (td, 2H, *J* = 7.8, 1.7 Hz, H6,6′), 7.65 (d, 2H, *J* = 8.1 Hz, H5,5′), 7.09 (ddd, 2H, *J* = 7.3, 5.8, 1.4 Hz, H7,7′),6.71 (s, 1H, H1), 2.55 (s, 6H, CH_3_) ppm. ^13^C NMR (75 MHz, CD_2_Cl_2_): *δ* = 167.02 (not assigned), 164.55 (not assigned), 139.24 (C6,6′), 135.33 (not assigned), 132.65 (C1), 122.07 (C7,7′), 121.55 (C5,5′), 21.79 (CH_3_) ppm. EI-MS(+) *m*/*z* = 352 (25%) [M]^+^, 317 (100%) [M‒Cl]^+^, 259 (25%) [M‒NiCl]+, 245 (25%) [M‒NiCl‒CH_3_]^+^, 229 (5%) [PyPhPy]^+^, 180 (5%) [M‒NiCl‒Py]^+^, 167 (5%) [PyPhCH_3_]^+^, 151 (5%) [PhPy]^+^, 129 (5%) [M‒L + Cl]^+^.

**Synthesis of [Pd(Me_2_dpb)Cl]:** In an inert flask equipped with a water trap filled with molecular sieves (3 Å), 0.186 g [Pd(COD)Cl_2_] (0.65 mmol, 1.3 eq.), 0.491 g KOAc (0.5 mmol, 1.0 eq.), and 0.693 g K_2_CO_3_ (0.5 mmol, 1.0 eq.) were prepared and 0.130 g 1,5-di(2-pyridyl)-2,4-dimethylbenzene (0.5 mmol, 1.0 eq.) as well as dry *p*-xylene (120 mL) were added and heated under reflux for 72 h under the strict exclusion of light. After cooling to ambient temperature, the precipitated greyish-yellow solid was filtered off, washed once with *p*-xylene, and the product was extracted using CH_2_Cl_2_. The solvent was removed and the pale orange solid was isolated (0.178 g, 0.44 mmol, 68%). Elemental analysis found (calculated for C_18_H_15_N_2_PdCl, M = 401.20 g mol^−1^): C, 53.82 (53.89); H, 4.27 (4.28); and N, 6.94 (6.98). ^1^H NMR (300 MHz, CD_2_Cl_2_): *δ* = 9.09 (d, 2H, *J* = 4.8 Hz, H8,8′), 7.85 (td, 2H, *J* = 7.8, 7.3, 1.7 Hz, H6,6′), 7.79 (d, 2H *J* = 7.8 Hz, H5,5′), 7.18 (ddd, 2H, *J* = 7.1, 5.4, 1.4 Hz, H7,7′), 6.65 (s, 1H, H1), and 2.55 (s, 6H, CH_3_) ppm. ^13^C NMR (75 MHz, CD_2_Cl_2_): *δ* = 177.17 (not assigned), 173.98 (not assigned), 152.88 (C8,8′), 139.23 (C6,6′), 136.77 (not assigned), 132.62 (C1), 122.79 (C7,7′), 122.67 (C5,5′), and 22.67 (CH_3_) ppm. EI-MS(+) *m*/*z* = 400 (20%) [M]^+^, 365 (100%) [M‒Cl]^+^, 259 (100%) [M‒PdCl]^+^, 245 (40%) [M‒PdCl‒CH_3_]^+^, 229 (5%) [PyPhPy]^+^, 180 (10%) [M‒PdCl‒Py]^+^, 167 (10%) [PyPhCH_3_]^+^, 151 (5%) [PhPy]^+^, and 78 (5%) [Py]^+^.

**Synthesis of [Pt(Me_2_dpb)Cl]**: 0.166 g (0.40 mmol, 1.0 eq.) K_2_PtCl_4_ and 0.104 g (0.4 mmol, 1.0 eq.) 1,5-di(2-pyridyl)-2,4-dimethylbenzene were suspended in 15 mL glacial acetic acid and refluxed for 3 d. The reaction mixture was then cooled down to room temperature and the precipitated bright orange solid was filtered off, washed with MeOH, H_2_O, EtOH and Et_2_O, and dried over P_4_O_10_ at a reduced pressure yielding 0.135 mg (0.28 mmol, 70%). Elemental analysis found (calculated for C_18_H_15_N_2_PtCl, M = 489.87 g mol^−1^): C, 43.14 (44.13); H, 3.06 (3.09); N, 5.75 (5.72). ^1^H NMR (300 MHz, CD_2_Cl_2_): *δ* = 9.39 (dd, 2H, *J* = 5.7, 2.3 Hz, *J*_PtH_ = 42 Hz, H8,8′), 7.93 (ddd, 2H, *J* = 7.8, 7.3, 1.8 Hz, H6,6′), 7.85 (d, 2H, *J* = 7.7 Hz, H5,5′), 7.24 (ddd, 2H, *J* = 7.4, 5.7, 1.6 Hz, H7,7′), 6.79 (s, 1H, H1), 2.64 (s, 6H, CH_3_) ppm. ^13^C NMR (75 MHz, CD_2_Cl_2_): *δ* = 168.83, 164.19 (not assigned), 152.49 (C8,8′), 139.37 (C6,6′), 137.27 (not assigned), 131.19 (C1), 123.00 (C7,7′), 122.83 (C5,5′), 22.27 (CH_3_) ppm. ^195^Pt NMR (64 MHz, DMSO-*d*_6_): *δ* = −3609.20. EI-MS(+) *m*/*z* = 489 (60%) [M]^+^, 454 (100%) [M‒Cl]^+^, 438 (20%) [M‒Cl‒CH_3_]^+^, 424 (10%) [M‒Cl‒2CH_3_]^+^, 259 (60%) [M‒PtCl]^+^, 245 (60%) [M‒PtCl‒CH_3_]^+^, 229 (5%) [PyPhPy]^+^, 180 (10%) [M‒PtCl‒Py]^+^, 167 (10%) [PyPhCH_3_]^+^, 151 (5%) [PhPy]^+^, 78 (5%) [Py]^+^.

**Alternative synthesis of[Pd(Me_2_dpb)Cl]:** 0.112 g (0.43 mmol, 1.0 eq.) of 1,5-di(2-pyridyl)-2,4-dimethylbenzene and 0.140 g (0.43 mmol, 1.0 eq.) K_2_[PdCl_4_] were suspended in 30 mL HOAc and heated under reflux for 22 h. The precipitated solid was filtered; washed with HOAc, MeOH, and Et_2_O (20 mL each), and then dried under vacuum. The product was isolated as a pale orange solid (0.168 g, 0.42 mmol, 98%). Elemental analysis found (calculated for C_18_H_15_N_2_PdCl, M = 401.20 g mol^−1^): C, 53.88 (53.89); H, 4.29 (4.28); N, 6.97 (6.98). NMR and MS characterisation gave identical values to those reported above.

**Attempted syntheses of [Pd(dpb)Cl]**: We also tried to cyclopalladate the unsubstituted 1,3-dipyridyl-benzene (dpbH) protoligand using our base-assisted method, starting from the Pd precursors PdCl_2_, K_2_[PdCl_4_], or [Pd(COD)Cl_2_] (Scheme S1). However, the target complex was not obtained. Instead, we isolated the previously reported tetranuclear complex [Pd_4_(µ-κ^2^,κ^2^dpb)_2_(µ-κ^1^,κ^1^-OAc)_4_] (see Scheme 2) and its unprecedented pivalate derivative. Details are provided in the Supplementary Materials.

## 4. Conclusions

In this study, we completed the series of homologous complexes [M(Me_2_dpb)Cl] containing the tridentate *N*,*C*,*N*-coordinating 3,5-dimethyl-2,6-dipyridyl-phenide ligand (Me_2_dpb^−^) by adding the Ni(II) and Pd(II) derivatives to the previously reported Pt(II) complex. All three complexes were synthesised using a base-assisted C‒H activation method starting from the protoligand 1,5-di(2-pyridyl)-2,4-dimethylbenzene (Me_2_dpbH), metal chlorides, and KOAc and K_2_CO_3_ in vigorously dried *p*-xylene.

Quite invariant electrochemical reductions around −2.3 V are essentially constant and DFT calculations confirmed the expected ligand-π*-centred reductions. The lowest unoccupied molecular orbital (LUMO) for all three complexes is very similar and the DFT-calculated energies of about −2.3 eV perfectly match the experimental results when using the TPSSh functional. The oxidation potentials increased along the series Pt < Ni < Pd from 0.15 to 0.74 V and the DFT-calculated highest occupied molecular orbitals (HOMO) confirm the marked contributions from metal d_yz_ orbitals alongside with phenyl C4, C2, C1, and C6, and Cl p_z_ contributions. As expected, the d_z_^2^ (HOMO-1 for Ni) is stabilised for the Pd and Pt derivative while the anti-bonding d_x_^2^_−y_^2^ orbital is destabilised in line with the larger ligand field splitting of Pd and Pt.

The experimental long-wavelength UV-vis absorption energies increased along the series Ni < Pt < Pd, matching well with the observed colours of the compounds ranging from red to yellow. The TD-DFT calculated absorption energies are only slightly red-shifted if compared with the experimental data. The lowest-energy transition calculated for the Ni complex has a pronounced d_z_^2^-type contribution to the overall metal-to-ligand charge transfer (MLCT) character of the excited configuration. For the Pt and Pd compounds, the d_z_^2^ is energetically not available and strongly mixed Cl-to-π*/phenyl-to-π*/M(d_yz_)-to-π* (XLCT/ILCT/MLCT) states were found. Contributions of the antibonding metal d_x_^2^−_y_^2^ orbitals to dissociative states were found for Ni at around 390 nm (3.18 eV), while such states were found at 281 nm (4.41 eV) for Pd and at 255 nm (4.86 eV) for Pt, in line with the expected trends regarding the ligand field splitting. The observed lowest energy absorption bands were assigned to the almost purely (95%) HOMO→LUMO+1 configurations for the Pt and Pd complexes, based on the calculated vertical S_0_→S_1_ transitions; for the Ni derivative, the transition leads to an excited state with the HOMO-2→LUMO (52%) and HOMO→LUMO+1 (43%) character that matches well with the observed long-wavelength bands. The HOMO→LUMO (for Pt and Pd) and HOMO-1→LUMO (Ni) states were assigned to absorptive transitions with low probability and match very well the so-called optical cut-off in the spectra. This confirms the versatility of the TPSSh functional for the full Ni-Pd-Pt triad.

The previously reported Pt complex [Pt(Me_2_dpb)Cl] showed triplet luminescence at 498 nm in fluid 2-Me-THF solutions at 298 K, which shifted to 489 nm in frozen glassy 2-MeTHF matrices at 77 K. The Pd derivative showed a structured emission profile at 77 K, peaking at 468 nm with an outstanding PL quantum yield reaching unity, as observed for the Pt derivative and the unsubstituted Pt complex [Pt(dpb)Cl]. No emission was observed for the Ni complex at 77 or at 298 K in any form (solution or solids). This is in agreement with the above-mentioned dissociative states at relatively low energies below 3.18 eV with contributions of a metal-centred character to the excited configurations, promoting rapid radiationless relaxation to the ground state. For the Pd(II) and Pt(II) derivatives, such “dark states” lie at markedly higher energies, namely above 4.41 eV for Pd and above 4.86 eV for Pt. Remarkably, the calculated vertical S_0_→S_1_ transitions show excited states with a contribution of the configuration involving the LUMO+4 for Pd (71%) and a markedly lower participation of the LUMO+5 on the excited state for Pt (38%), which is in line with the very different behaviour at 298 K. The more efficient radiationless decay of the Pd complex at 298 K is attributed to this higher antibonding d_x_^2^_−y_^2^-orbital participation if compared with the analogous Pt derivative. In contrast to this, at 77 K, the quite similar energies of the “dark” states for Pt and Pd are decisive, leading to very similar photoluminescence patterns.

In any case, the similar PL efficiency for a Pd(II) complex with its Pt(II) homologue is remarkable and rarely encountered. We will thus use the observed balance of energy + character + dynamics of excited states to design more Pd(II) complexes with efficient PL in future work.

## Figures and Tables

**Table 1:** Redox potentials of Me_2_dpbH, of complexes [M(Me_2_dpb)Cl] (M = Pt, Pd, Ni), and of comparable complexes ^a^.

	*E*_pc_ (Red2)	*E*_1/2_ (Red1)	*E*_pa_ (ox1)	Δ*E* (Red1‒Red2)	Δ*E* (ox1‒Red1)	Solvent	*DFT* ^k^
Me_2_dpbH	−2.52	−1.74 irr	-	0.70	-	THF	
[Ni(Me_2_dpb)Cl]	−2.37 rev ^b^	−2.26 irr	0.10 rev	0.11 (0.45) ^b^	2.36	THF	3.07
[Pd(Me_2_dpb)Cl]	−2.75	−2.34	0.74	0.41	3.07	THF	3.39
[Pt(Me_2_dpb)Cl]	−2.69	−2.24	0.35 ^c^	0.45	2.59	THF	3.15
[Pt(dpb)Cl] ^d^	-	−2.14	0.35	-	2.49	MeCN	
[Pt(4-Medpb)Cl] ^d^	-	−2.15	0.29	-	2.44	MeCN	
[Pt(4-MeOOCdpb)Cl] ^d^	-	−2.04	0.39	-	2.43	MeCN	
[Pt(dpb)Cl] ^e^	-	−2.18	0.41	-	2.59	DMF	
[Pt(3,5-(CF_3_)_2_dpb)Cl] ^e^	-	−1.92	0.50	-	2.42	DMF	
[Pt(3,4,5-F_3_dpb)Cl] ^e^	-	−2.07	0.51	-	2.58	DMF	
[Pt(Me_2_dpb)Cl] ^f^	-	−2.03	0.43	-	2.46	CH_2_Cl_2_	
[Ni(dpb)Cl] ^g^	−2.57	−2.33 irr	0.06 rev	0.24	2.39	THF	
[Ni(phbpy)Cl] ^h,i^	−2.60 rev	−1.93	0.06 rev	0.67	1.99	THF	
[Pd(phbpy)Cl)] ^i^	−2.60 rev	−1.92	0.80 ^b^	0.68	2.72	THF	3.58
[Pt(phbpy)Cl] ^i^	−2.48 rev	−1.78	0.41	0.70	2.19	THF	

^a^ From cyclic voltammetry in *n*-Bu_4_NPF_6_/THF (tetrahydrofuran). Potentials in V vs. ferrocene/ferrocenium; half-wave potentials (*E*_1/2_) for reversible processes (rev); cathodic peak potentials (*E*_pc_) for irreversible reductions (irr); accuracy of potentials: ± 0.003 V. ^b^ When taking into account the third irreversible reduction at −2.82 V, the separation between the second and third potential fits to the 0.45 V observed for the Pt and Pd derivatives. However, we assume that the [Ni(Me_2_dpb)(THF)]^+^ complex causes the second and third reduction processes (see text). ^c^ Further irreversible oxidation at 0.52 V. ^d^ From Reference [50]. ^e^ From Reference [54]. ^f^ Reduction measured in MeCN and oxidation in CH_2_Cl_2_ from Reference [60]. ^g^ From Reference [47]. ^h^ From Reference [70]. ^i^ From Reference [71]. ^k^ DFT-calculated HOMO-LUMO gaps for the *S*_0_ states.

**Table 2:** Selected UV-vis absorption maxima of Me_2_dpbH, the complexes [M(Me_2_dpb)Cl], and related derivatives ^a^.

	*λ*_1_ (ε)	*λ*_2_ (*ε*)	*λ*_3_ (*ε*)	*λ*_4_ (*ε*)	*λ*_5_ (*ε*)	*λ*_6_ (*ε*)	*λ*_7_ (*ε*)
Me_2_dpbH*—this work*	227 (28.2)	260 (19.7)	340 (0.4)	636 (0.3)	-	-	-
[Pt(Me_2_dpb)Cl]*—this work*	231 (33.7)	256 (30.0)	287 (22.9)	331 (9.2)	380 (8.3)	413 (1.5)	480 (0.1)
[Pd(Me_2_dpb)Cl]*—this work*	239 (26.7)	275 (23.2)	283 (22.0)	327 (8.3)	360 (7.4)	375 (1.2)	-
[NiMe_2_dpb)Cl]—*this work*	230 (33.0)	274 (31.2)	297 (14.7)	333 (9.0)	395 (6.1) ^b^	-	465 (1.1)
[Pt(dpb)Cl] ^c,e,e,f^	-	255 (25.2)	289 (21.1)	332 (6.3)	379 (8.6)	402 (7.0)	485 (0.1)
[Pt(4-Medpb)Cl] ^d^	-	-	-	335 (5.7)	381 (6.9)	412 (6.8)	495 (0.1)
[Pt(4-MeOOCdpb)Cl] ^d^	-	-	-	329 (7.5)	380 (9.9)	397 (7.9)	478 (0.2)
[Pt(3-F-dpb)Cl] ^e^	-	-	-	-	379 (9.1)	401 (5.2)	477 (0.1)
[Pt(3,5-F_2_-dpb)Cl] ^e^	-	-	-	-	375 (7.6)	-	467 (0.1)
[Pt(3,4,5-F_3_-dpb)Cl] ^c^	-	-	-	-	380 (10.4)	405 (4.6)	480 (0.1)
[Pt(3,5-(CF_3_)_2_-dpb)Cl] ^c^	-	-	-	-	382 (10.4)	408 (8.6)	479 (0.2)
[Ni(dpb)Cl] ^g^	236 (35.5)	279 (26.8)	-	332 (5.7)	412 (6.3)	437 (6.7)	
[Ni(phbpy)Cl] ^g,h^	281		321	354	391	596	
[Pd(phbpy)Cl] ^h,i,k^	266 (20)	278 (21)	311 (12)	325 (13)	342 (8)	402 (1)	
[Pt(phbpy)Cl] ^h,i^	278	302	330	363	410	430	

^a^ Absorption maxima *λ* in nm in fluid CH_2_Cl_2_ solution at rt; molar absorption coefficient *ε* in 1000 L mol^−1^ cm^−1^. ^b^ Further shoulder at 420 (5.3). ^c^ From Reference [54]. ^d^ From Reference [50]. ^e^ From Reference [58]. ^f^ From Reference [72]. ^g^ From Reference [47]. ^h^ From Reference [70]. ^i^ From Reference [71]. ^k^ From Reference [42].

**Table 3:** Photoluminescence data of the investigated Pt and Pd complexes ^a^.

		[Pt(dpb)Cl]			[Pt(Me_2_dpb)Cl]			[Pd(Me_2_dpb)Cl]
Solvent		DCM	2-MeTHF		DCM	2-MeTHF		2-MeTHF
Temperature		298 K	298 K	77 K	298 K	298 K	77 K	77 K
*λ*_Em_/nm		490, 523, 560sh	494, 526, 565sh	486, 503, 520, 560sh	495, 527, 565	498, 530, 567sh	489, 503, 522, 560sh	468, 481, 501, 531sh
*λ*_Exc_/nm		289, 330sh, 381, 403sh	298, 330sh, 388, 415	295, 335, 380, 410	300, 342, 390	302, 343, 394	303, 343, 373, 390	300, 347, 373
*τ*_av_/µs ^b^	Air-equilibrated	0.4464 ± 0.0015	0.1659 ± 0.0006	5.717 ± 0.016	0.3428 ± 0.0015	0.1185 ± 0.0006	5.794 ± 0.017 ^c^	152.8 ± 0.6 [157 ± 3 (92); 102 ± 12 (8)] ^d^
	Deaerated	6.050 ± 0.019	3.842 ± 0.009		6.420 ± 0.016	4.608 ± 0.010		
*Φ*_L_ ± 0.02	Air-equilibrated	0.04	<0.02	0.98	0.04	< 0.02	0.98	0.98
	Deaerated	0.64	0.52		0.75	0.64		

^a^ The Ni complex did not show any photoluminescence under these conditions. The Pd complex was not emissive at 298 K. ^b^ Photoluminescence decay measured at 495 nm, except: ^c^ 485 nm or ^d^ 470 nm (for multiexponential decays, the amplitude-weighted average lifetimes are given, as well as the different components in square brackets with their relative amplitudes indicated as percentages in parentheses). Photoluminescence quantum yields *Φ*_L_. Main band maxima are underlined. Abbreviation: sh, shoulder.

## Data Availability

Data might be requested directly from the authors.

## Supplementary Materials

The following information is available online: Supplementary Material contains 25 Figures and 21 Tables with crystal and molecular structures, cyclic voltammogramms, UV-vis absorption spectra, steady-state photoluminescence spectra and time-resolved photoluminescence decays, as well as UV-vis absorption spectra of reduced and oxidises species; Supplementary Material II contains 27 Figures with NMR and MS characterisation of Ni, Pd, and Pt complexes including Pd side-products.

## Sample Availability

Samples of the compounds are not available.
