# Supplementary material for: Photoluminescence of Ni(II), Pd(II), and Pt(II) Complexes [M(Me2dpb)Cl] Obtained from C‒H Activation of 1,5-Di(2-pyridyl)-2,4-dimethylbenzene (Me2dpbH)
Source: Molecules. 2021 Aug 20;26(16):5051. doi: 10.3390/molecules26165051 (PMC8401505; doi:10.3390/molecules26165051)
Supplement: Supplementary file 1 [file molecules-26-05051-s001.zip › Supplement II Ni Pd Pt Me2DPB Cl.pdf]

Supplementary Materials II – NMR and MS data
for
Photoluminescence of Ni(II), Pd(II), and Pt(II) complexes [M(Me_2dpb)Cl] obtained from C–H activation of 1,5-di(2-pyridyl)-2,4-dimethylbenzene (Me_2dpbH)

Contents:

Figure. S001. 300 MHz ^{1}H-NMR spectrum of 1,5-di(2-pyridyl)-2,4-dimethylbenzene measured in DMSO-d_6 at RT.
Figure. S002. 75 MHz ^{13}C-NMR spectrum of 1,5-di(2-pyridyl)-2,4-dimethylbenzene measured in DMSO-d_6 at RT.
Figure. S003. H,C-HSQC spectrum of 1,5-di(2-pyridyl)-2,4-dimethylbenzene measured in DMSO-d_6 at RT.
Figure. S004. H,H-COSY spectrum of 1,5-di(2-pyridyl)-2,4-dimethylbenzene measured in DMSO-d_6 at RT.
Figure. S005. 300 MHz ^{1}H-NMR spectrum of [Ni(Me_2dpb)Cl] measured in CD_2Cl_2 at RT.
Figure. S006. 75 MHz ^{13}C-NMR spectrum of [Ni(Me_2)Cl] measured in CD_2Cl_2 at RT.
Figure. S007. H,C-HSQC spectrum of [Ni(Me_2dpb)Cl] measured in CD_2Cl_2 at RT.
Figure. S008. H,H-COSY spectrum of [Ni(Me_2dpb)Cl] measured in CD_2Cl_2 at RT.
Figure. S009. 300 MHz ^{1}H-NMR spectrum of [Pd(Me_2dpb)Cl] measured in CD_2Cl_2 at RT.
Figure. S010. 75 MHz ^{13}C-NMR spectrum of [Pd(Me_2dpb)Cl] measured in CD_2Cl_2 at RT.
Figure. S011. H,C-HSQC spectrum of [Pd(Me_2dpb)Cl] measured in CD_2Cl_2 at RT.
Figure. S012. H,H-COSY spectrum of [Pd(Me_2dpb)Cl] measured in CD_2Cl_2 at RT.
Figure. S013. 300 MHz ^{1}H-NMR spectrum of [Pt(Me_2dpb)Cl] measured in CD_2Cl_2 at RT.
Figure. S014. 75 MHz ^{13}C-NMR spectrum of [Pt(Me_2dpb)Cl] measured in CD_2Cl_2 at RT.
Figure. S015. H,C-HSQC spectrum of [Pt(Me_2dpb)Cl] measured in CD_2Cl_2 at RT.
Figure. S016. H,H-COSY spectrum of [Pt(Me_2dpb)Cl] measured in CD_2Cl_2 at RT.
Figure. S017. ^{195}Pt{1H} NMR spectrum of [Pt(Me_2dpb)Cl] measured in DMSO-d_6 at RT.
Figure. S018. H,Pt-HMBC spectrum of [Pt(Me_2dpb)Cl] measured in DMSO-d_6 at RT.
Figure. S019. MS-EI(+) of 1,5-di(2-pyridyl)-2,4-dimethylbenzene (Me_2dpbH)
Figure. S020. MS-EI(+) of [Pt(Me_2dpb)Cl]
Figure. S021. MS-EI(+) of [Pd(Me_2dpb)Cl]
Figure. S022. MS-EI(+) of [Ni(Me_2dpb)Cl]
Figure S023. 300 MHz ^{1}H NMR spectrum of [Pd_4(μ-κ^2,κ^{2}dpb)$_2$(μ-κ^1,κ^1-OAc)$_4$] in CD_2Cl_2.
Figure S024. 300 MHz ^{1}H NMR spectrum of [Pd_4(μ-κ^2,κ^{2}dpb)$_2$(μ-κ^1,κ^1-OAc)$_4$] with traces of [Pd(PyPhPy)Cl] in CD_2Cl_2.
Figure S025. EI-MS(+) of the product mixture of [Pd_4(μ-κ^2,κ^{2}dpb)$_2$(μ-κ^1,κ^1-OAc)$_4$] with traces of [Pd(PyPhPy)Cl].
Figure S026. 300 MHz ^{1}H NMR spectrum of [Pd_4(μ-κ^2,κ^{2}dpb)$_2$(μ-κ^1,κ^1-OPiv)$_4$] in DMSO-d_6.
Figure S027. UV-vis absorption spectra of [Pd_4(μ-κ^2,κ^{2}dpb)$_2$(μ-κ^1,κ^1-OAc)$_4$] and [Pd_4(μ-κ^2,κ^{2}dpb)$_2$(μ-κ^1,κ^1-OPiv)$_4$] in THF.

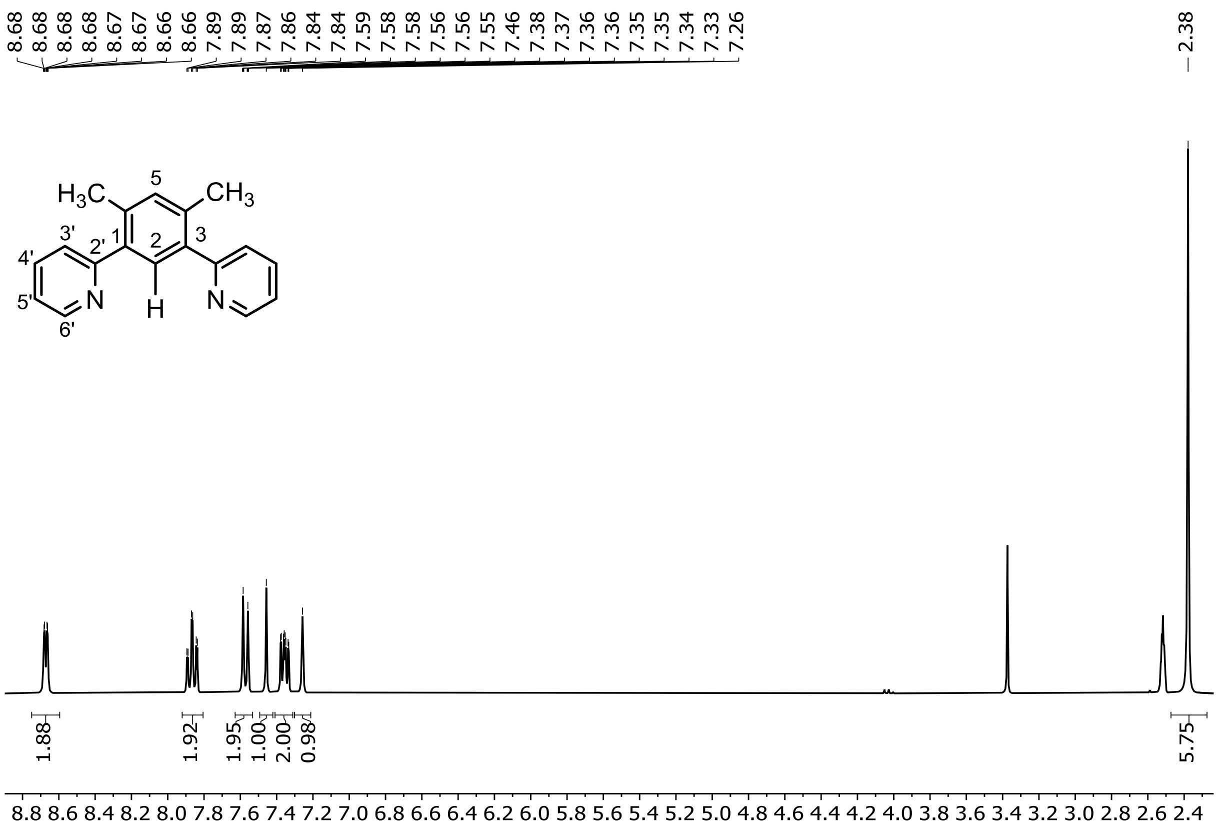

Figure. S001. 300 MHz ^{1}H-NMR spectrum of 1,5-di(2-pyridyl)-2,4-dimethylbenzene measured in DMSO-d_6 at RT.

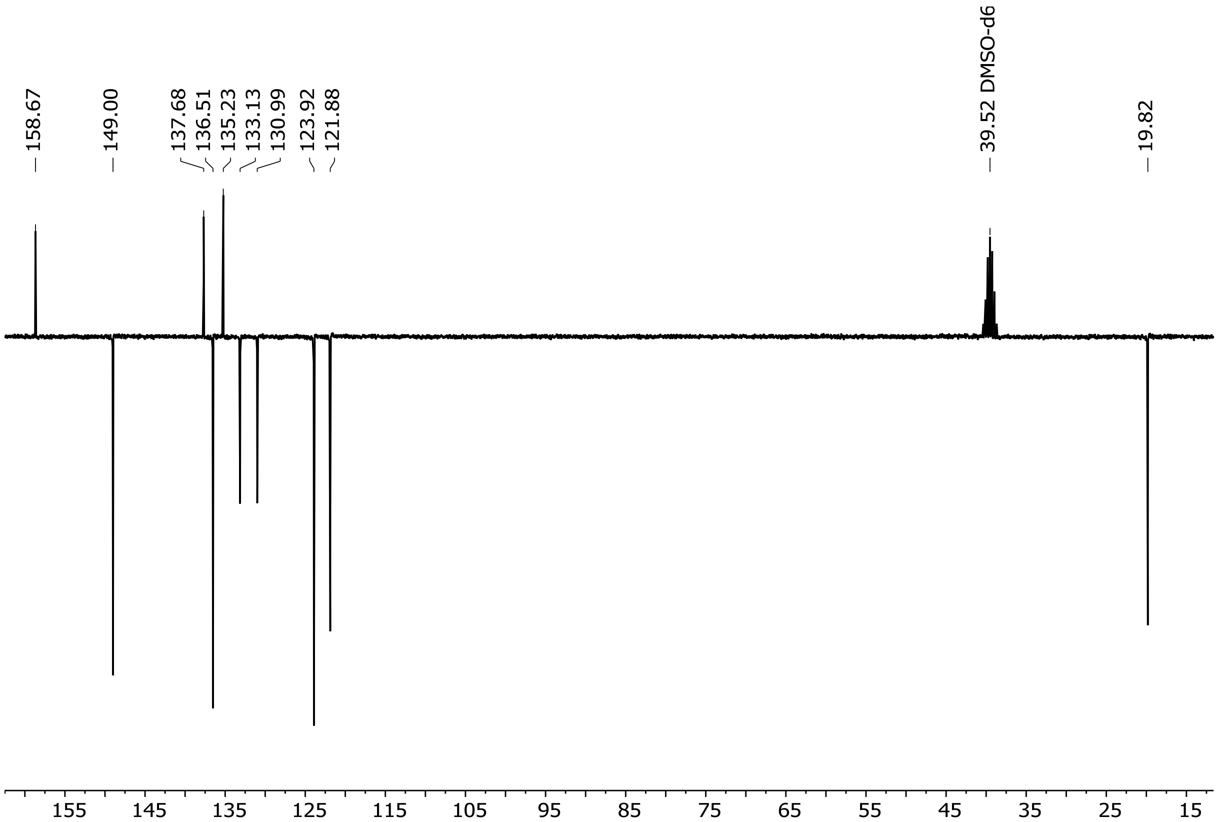

Figure. S002. 75 MHz ^{13}C-NMR spectrum of 1,5-di(2-pyridyl)-2,4-dimethylbenzene measured in DMSO-d_6 at RT.

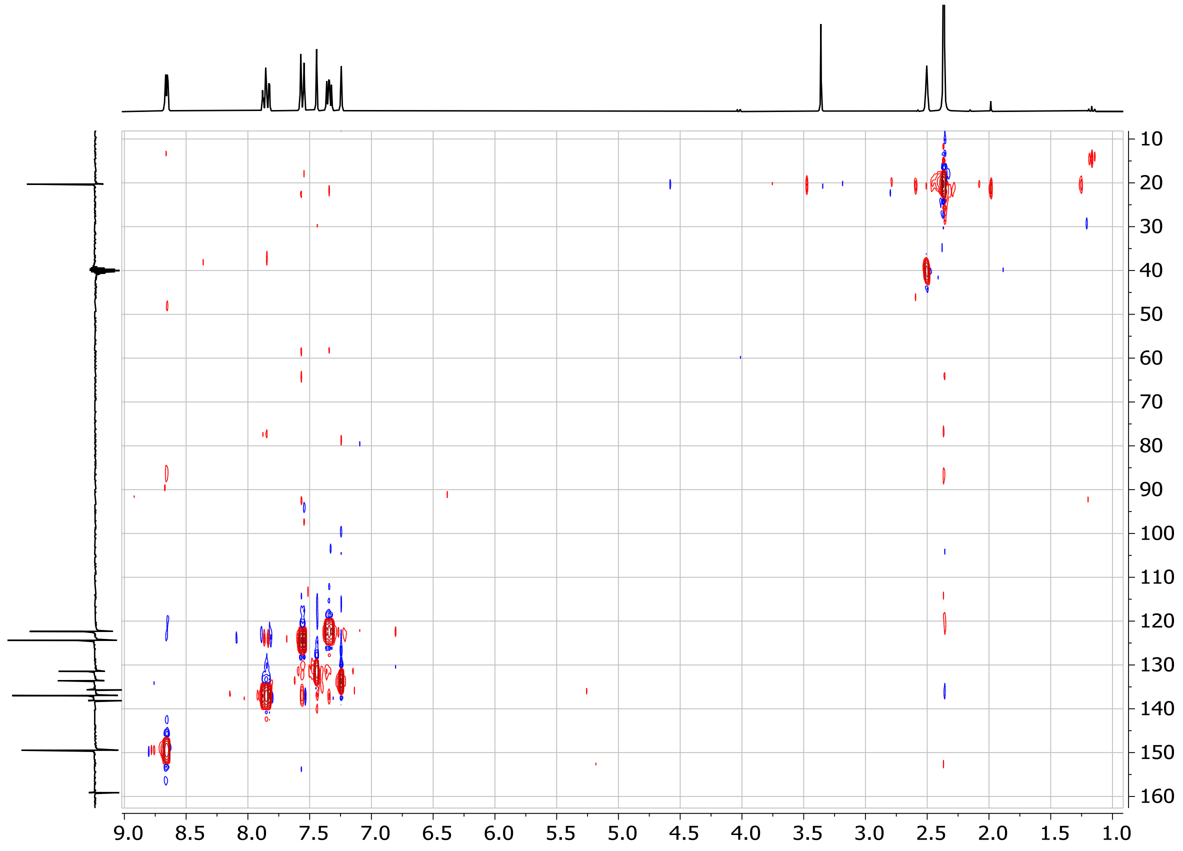

Figure. S003.H,C-HSQC spectrum of 1,5-di(2-pyridyl)-2,4-dimethylbenzene measured in DMSO-d_6 at RT.

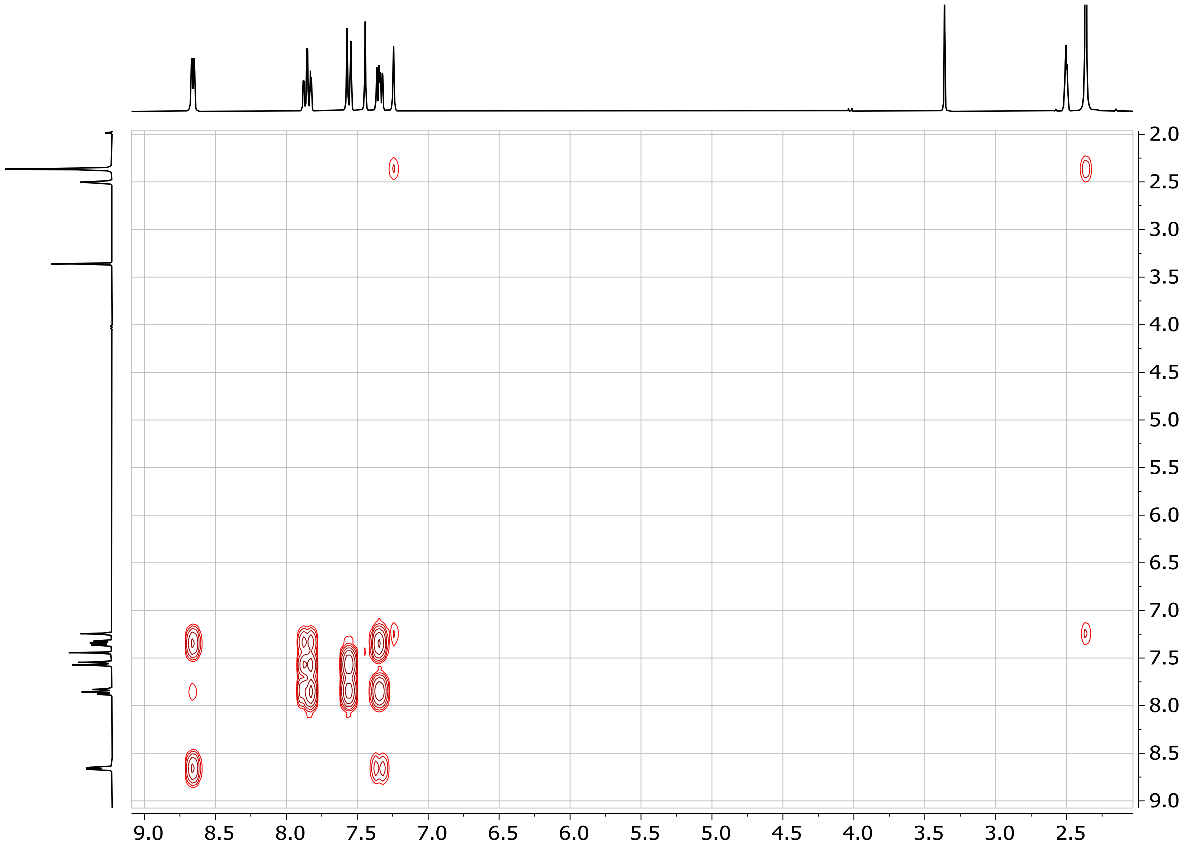

Figure. S004. H,H-COSY spectrum of 1,5-di(2-pyridyl)-2,4-dimethylbenzene measured in DMSO-d_6 at RT.

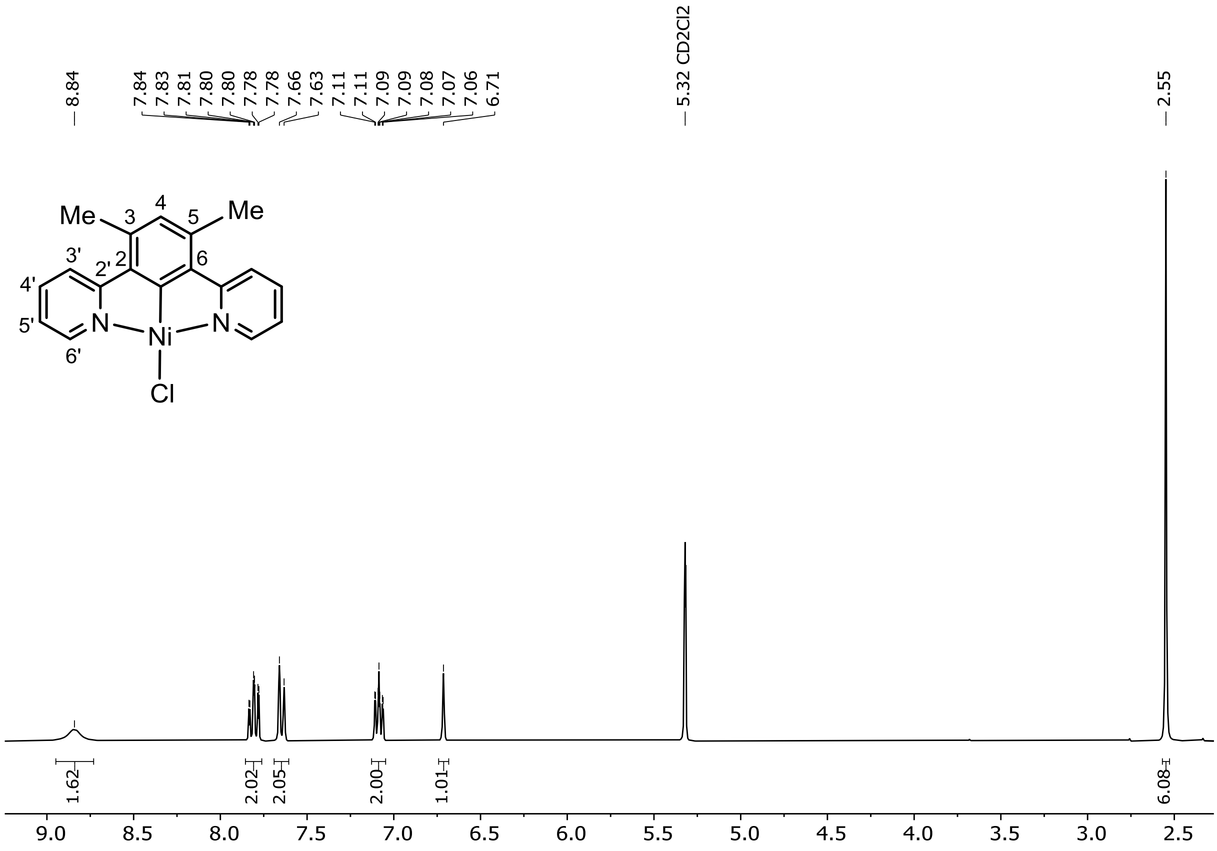

Figure. S005. 300 MHz ^{1}H-NMR spectrum of [Ni(Me$_2$dpb)Cl] measured in CD_2Cl_2 at RT.

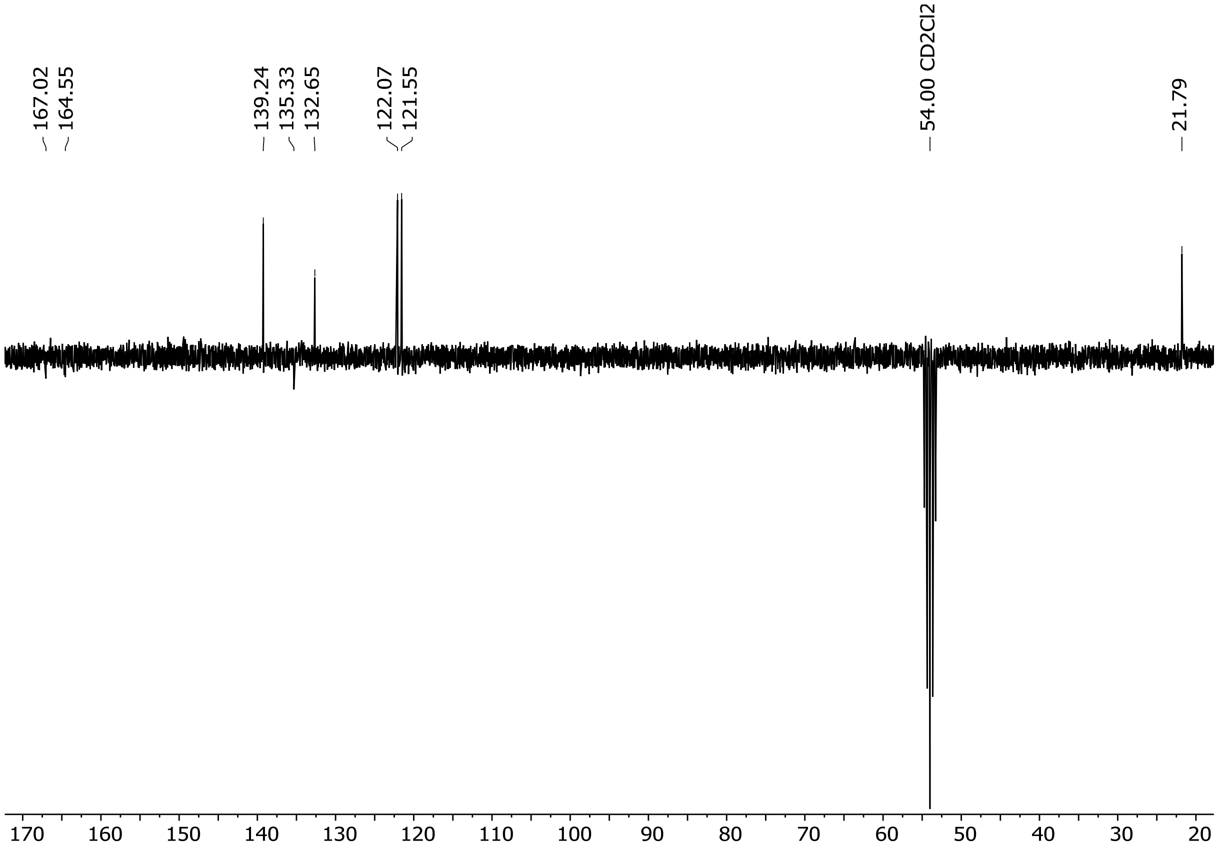

Figure. S006. 75 MHz ^{13}C-NMR spectrum of [Ni(Me$_2$dpb)Cl] measured in CD_2Cl_2 at RT.

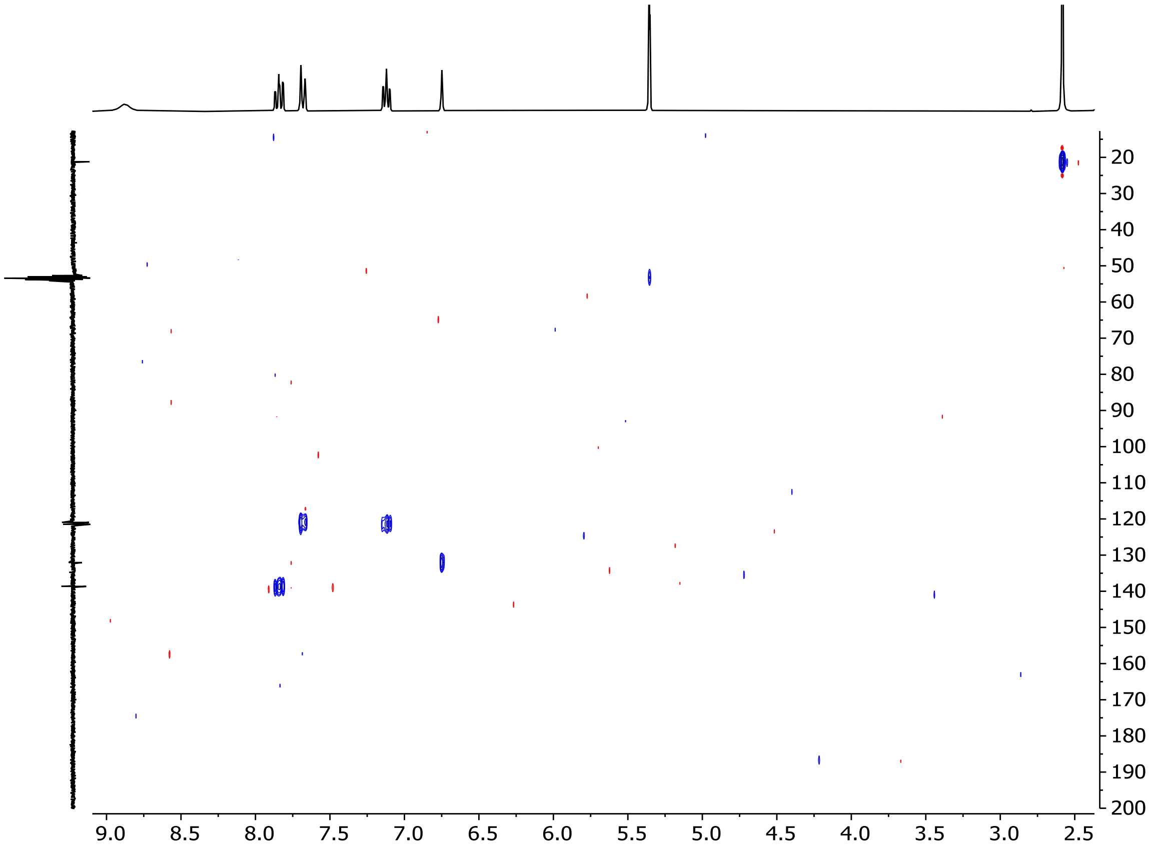

Figure. S007. H,C-HSQC spectrum of [Ni(Me2dpb)Cl] measured in CD_2Cl_2 at RT.

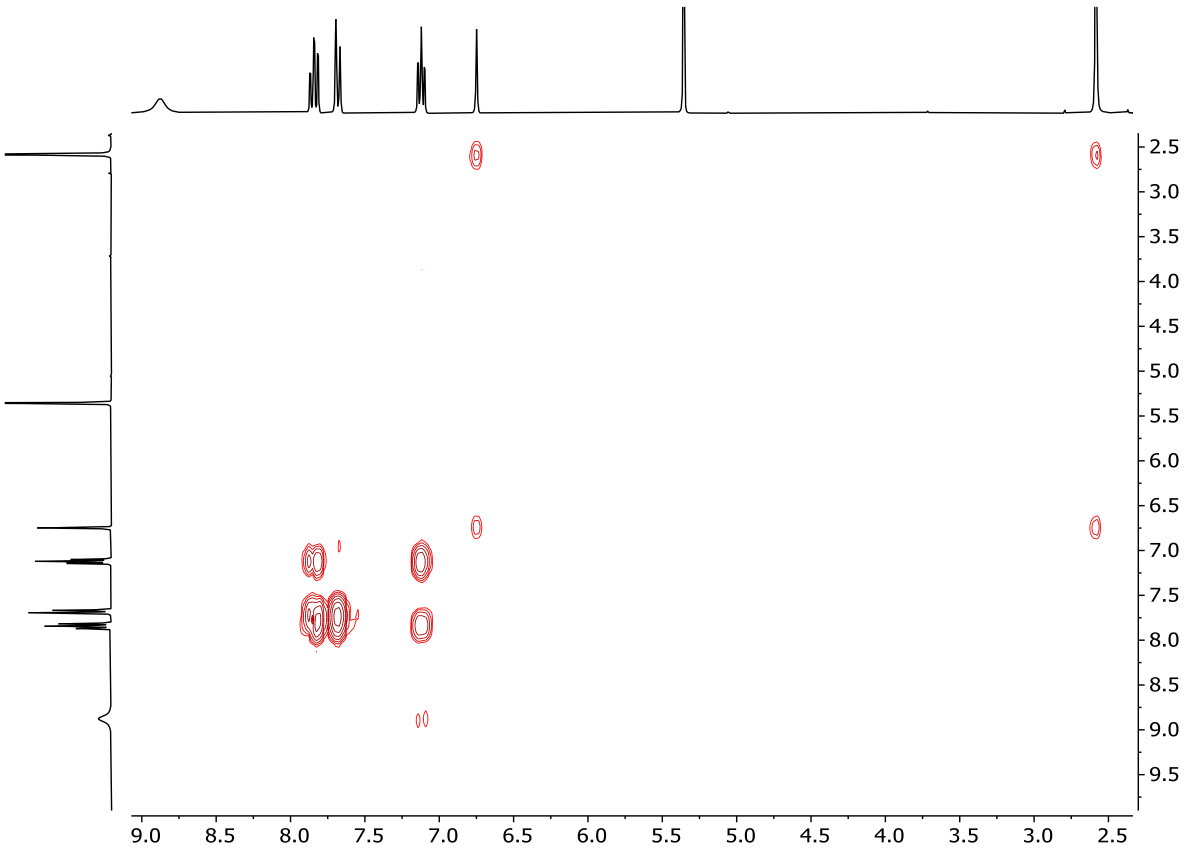

Figure. S008. H,H-COSY spectrum of [Ni(Me2dpb)Cl] measured in CD_2Cl_2 at RT.

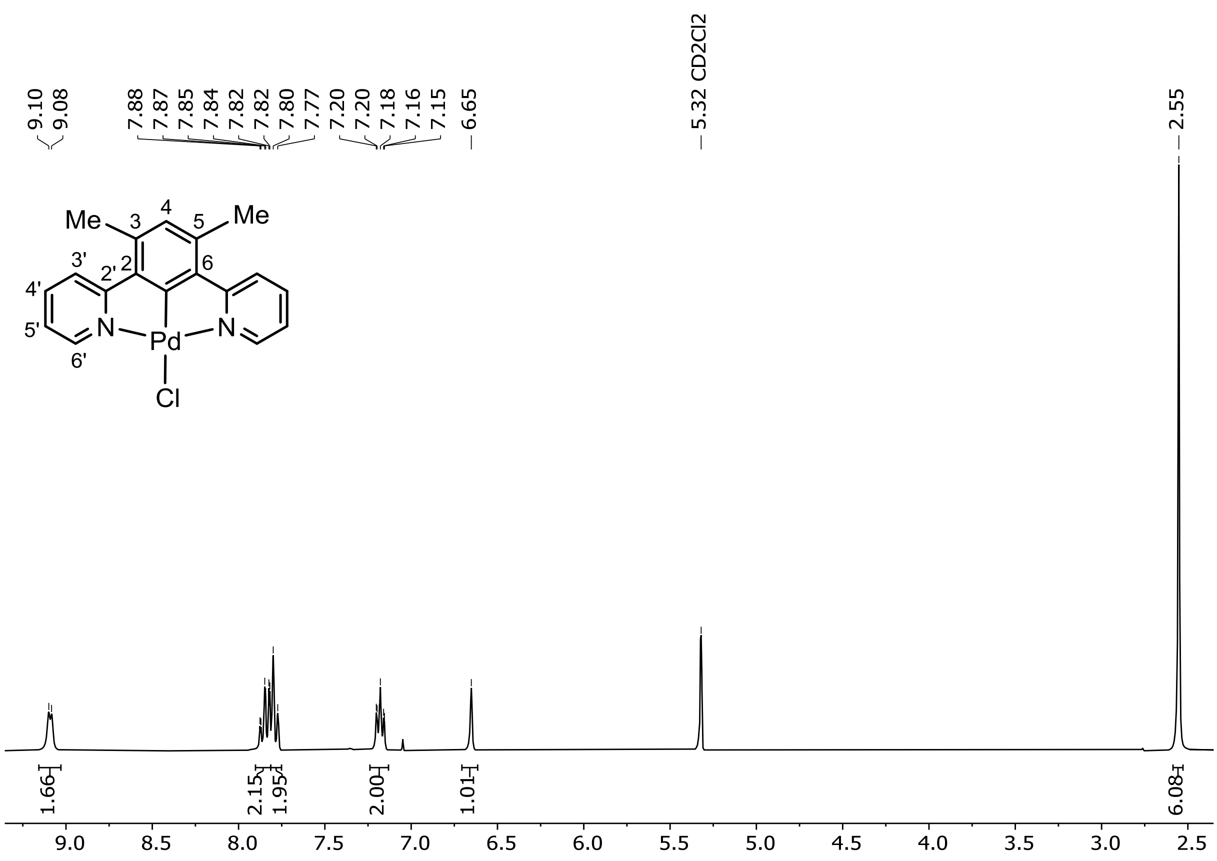

Figure. S009. 300 MHz ^{1}H-NMR spectrum of [Pd(Me$_2$dpb)Cl] measured in CD_2Cl_2 at RT.

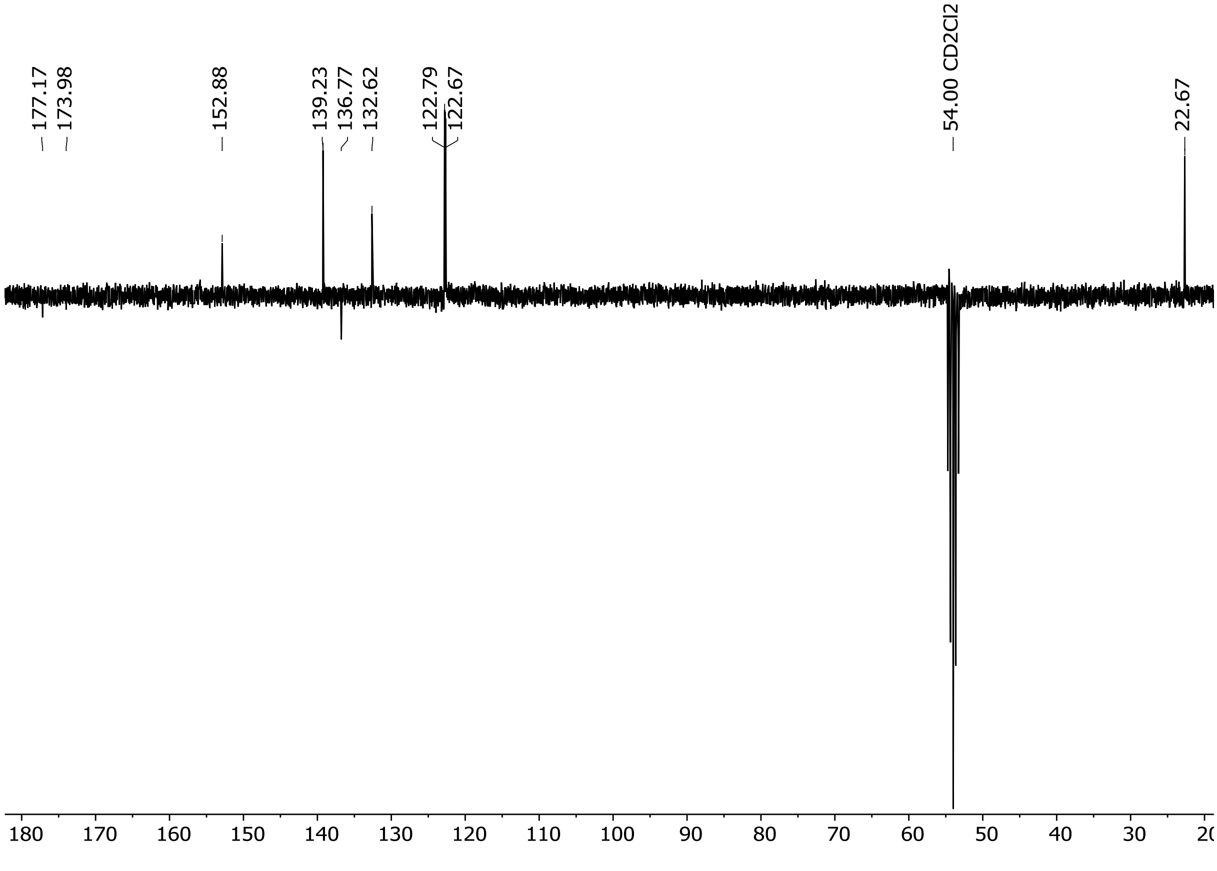

Figure. S010. 75 MHz ^{13}C-NMR spectrum of [Pd(Me$_2$dpb)Cl] measured in CD_2Cl_2 at RT.

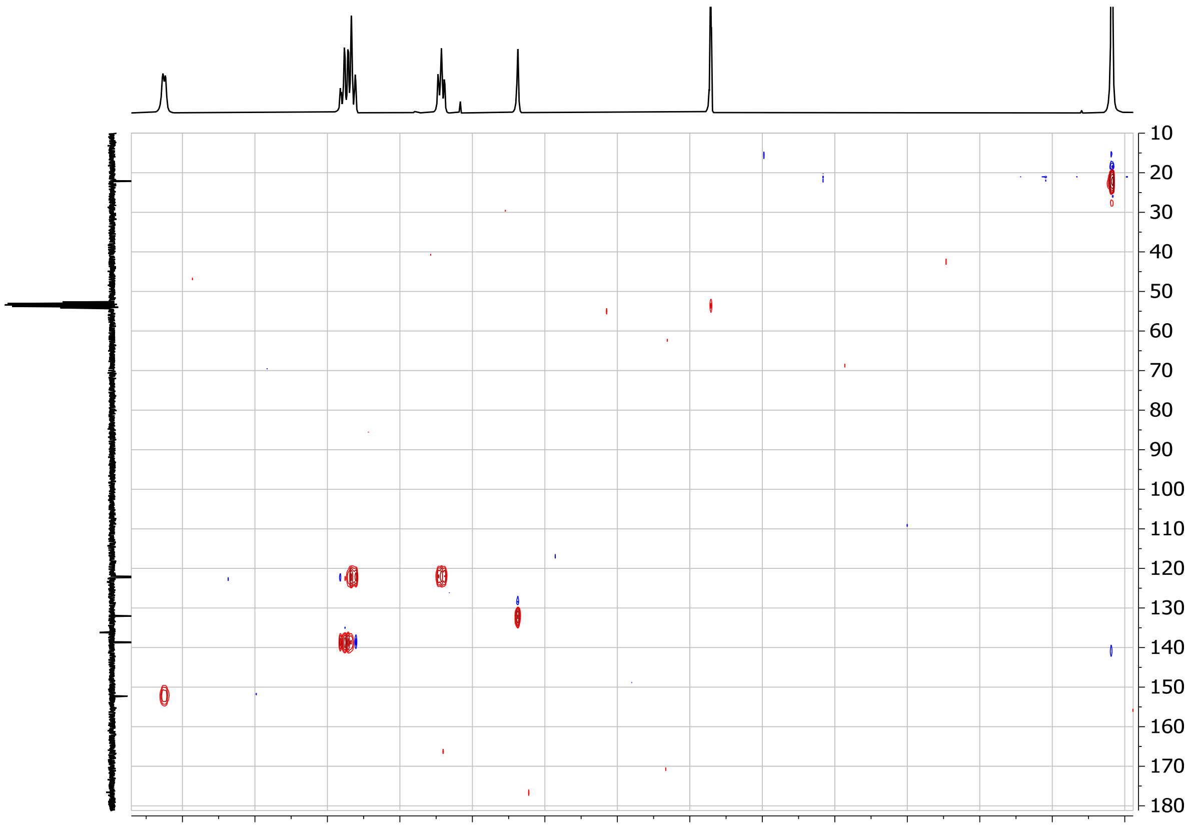

Figure. S011. H,C-HSQC spectrum of [Pd(Me_2dpb)Cl] measured in CD_2Cl_2 at RT.

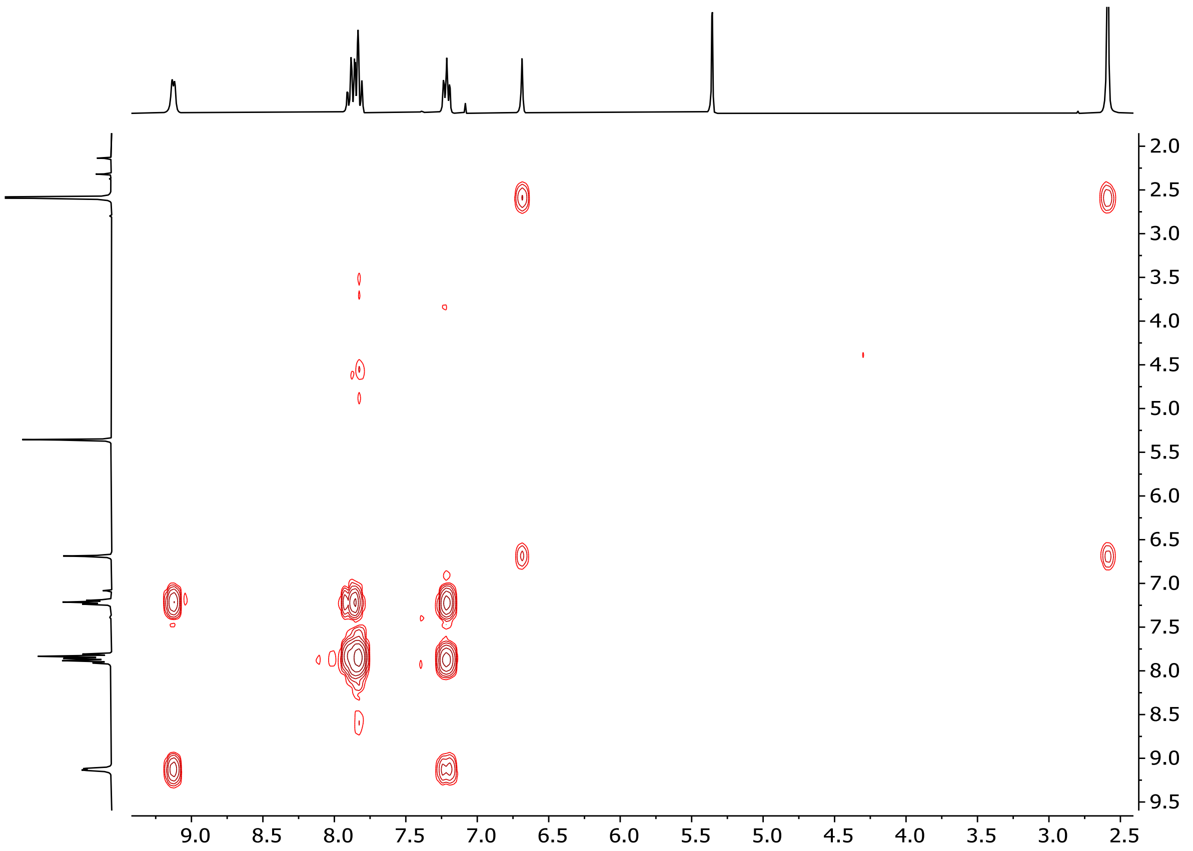

Figure. S012. H,H-COSY spectrum of [Pd(Me_2dpb)Cl] measured in CD_2Cl_2 at RT.

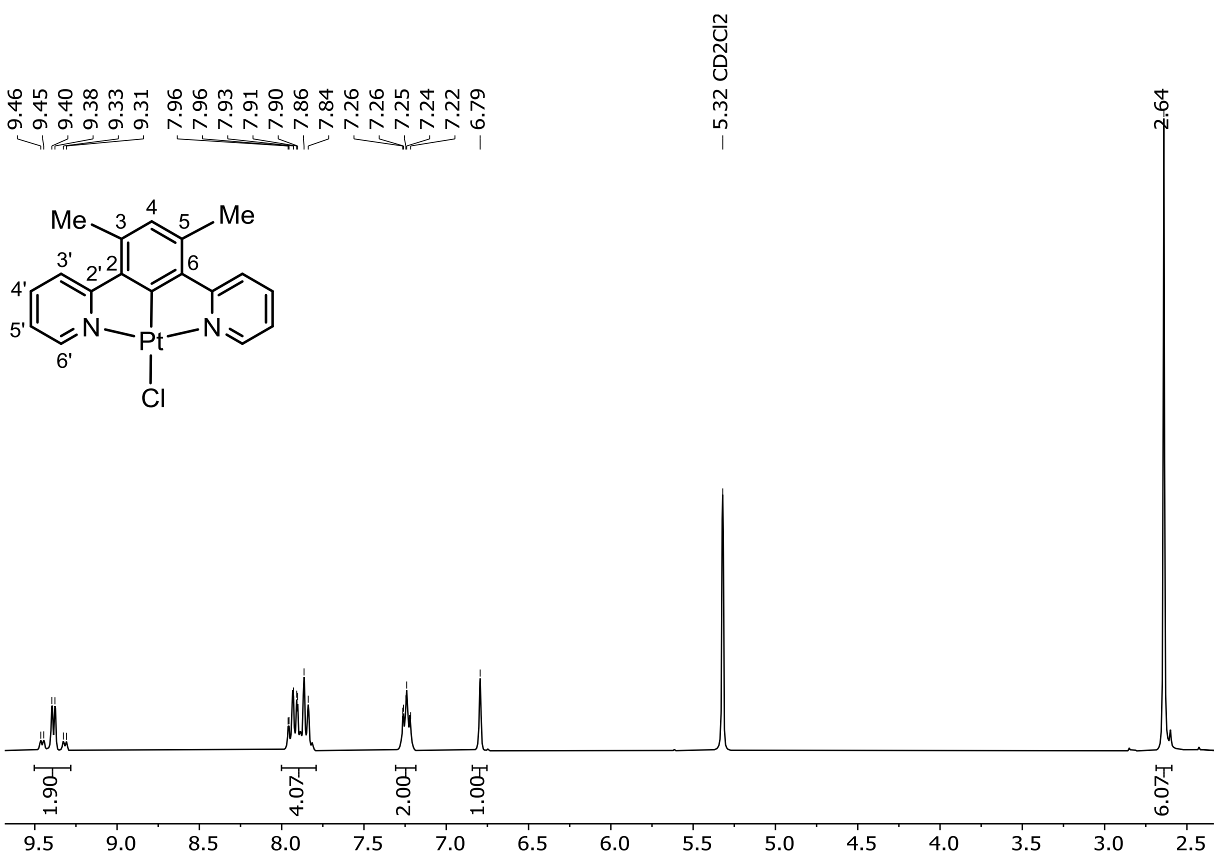

Figure. S013. 300 MHz ^{1}H-NMR spectrum of [Pt(Me$_2$dpb)Cl] measured in CD_2Cl_2 at RT.

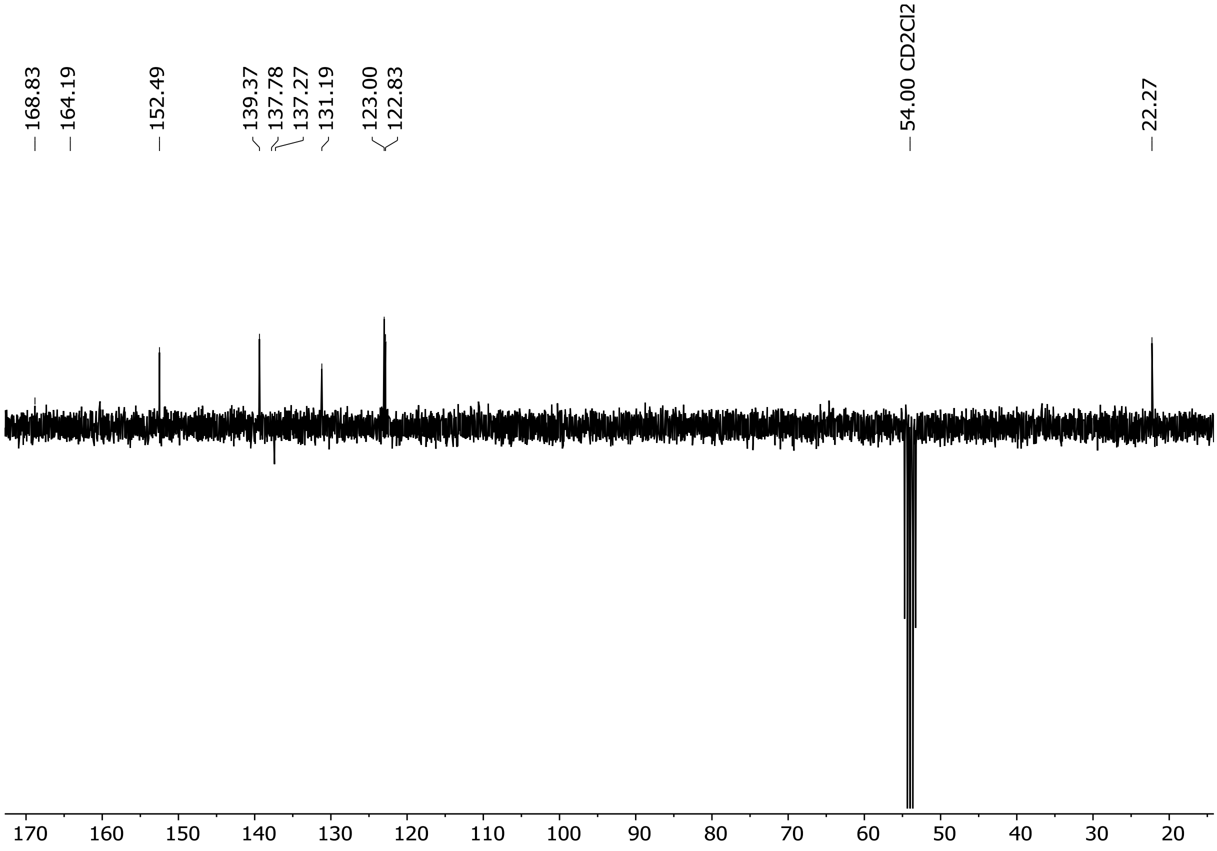

Figure. S014. 75 MHz ^{13}C-NMR spectrum of [Pt(Me$_2$dpb)Cl] measured in CD_2Cl_2 at RT.

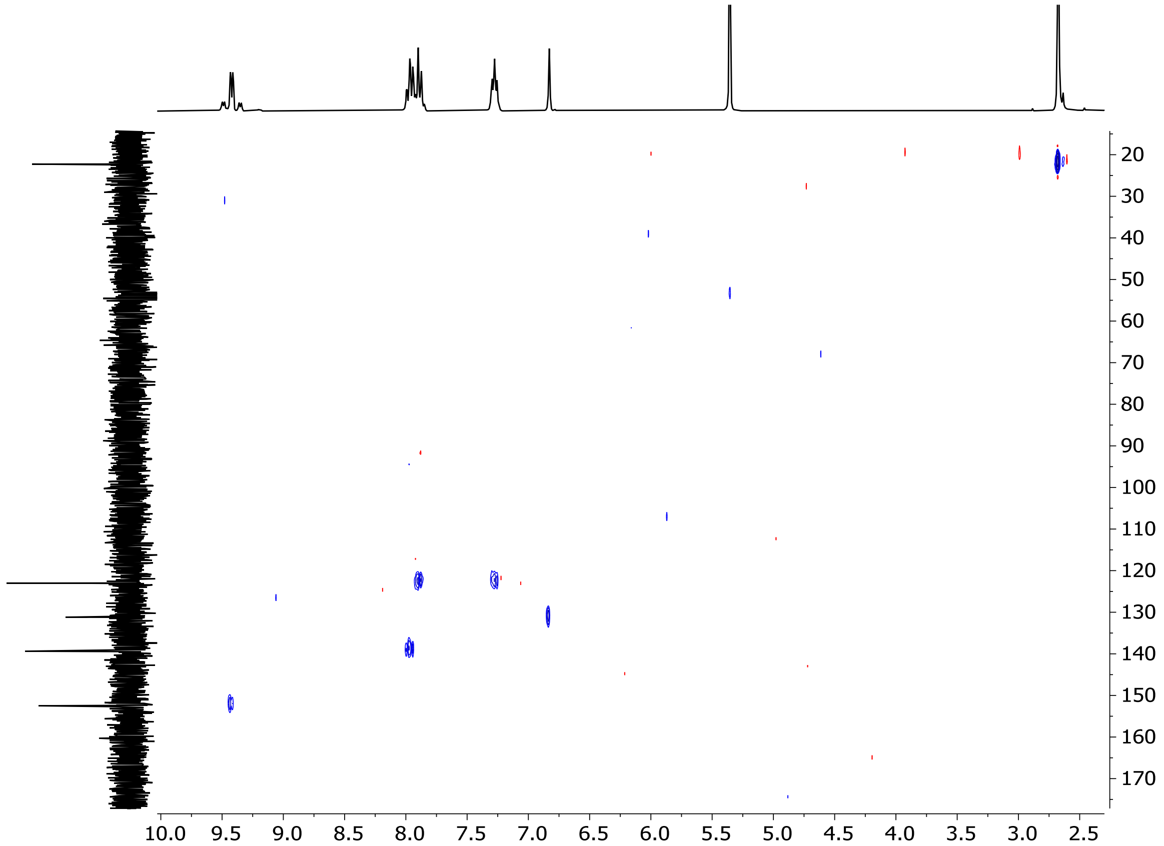

Figure. S015. H,C-HSQC spectrum of [Pt(Me_2dpb)Cl] measured in CD_2Cl_2 at RT.

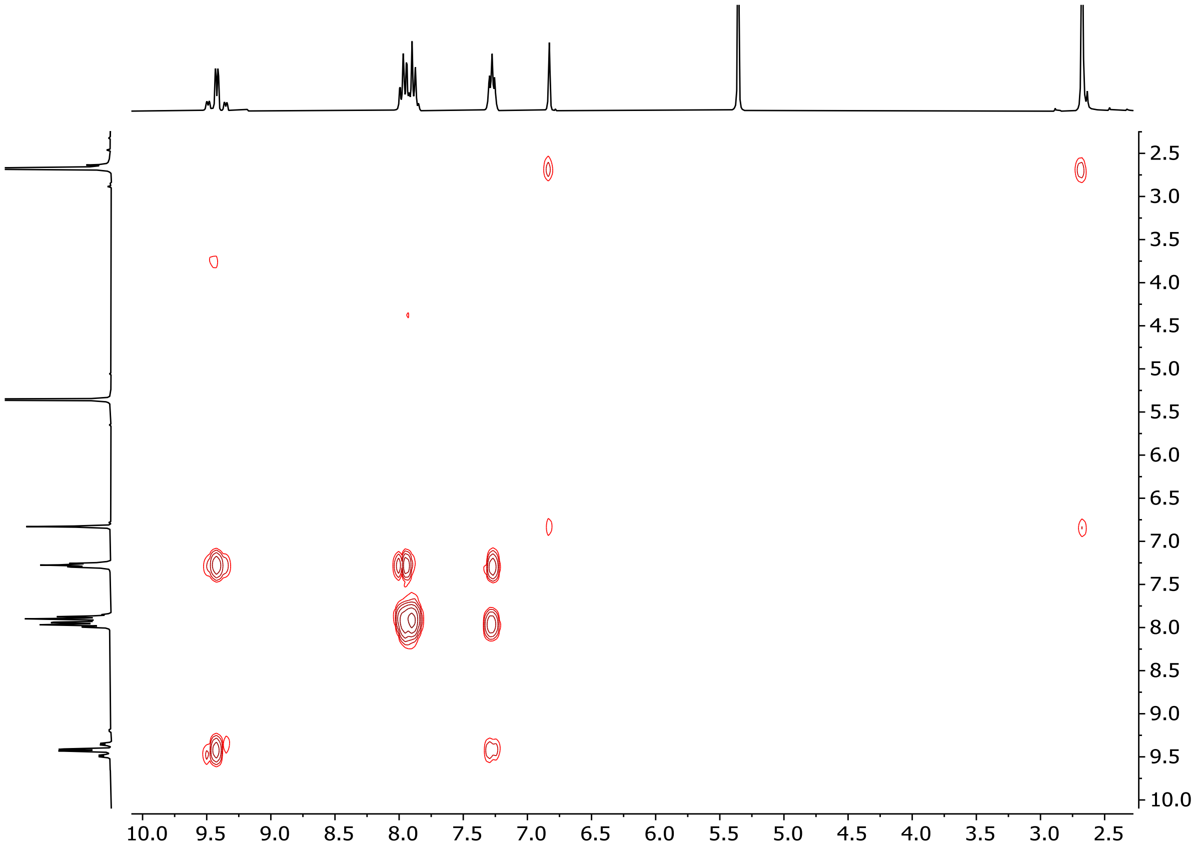

Figure. S016. H,H-COSY spectrum of [Pt(Me_2dpb)Cl] measured in CD_2Cl_2 at RT.

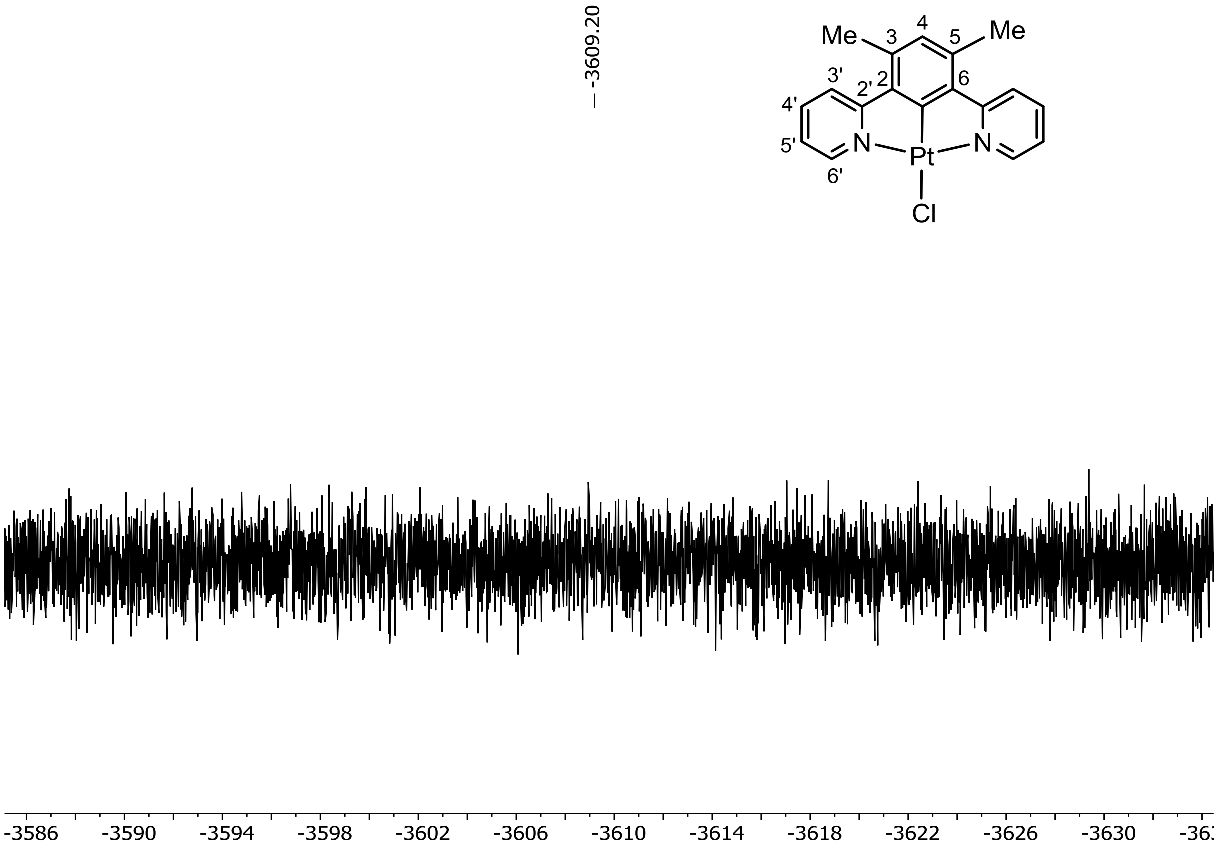

Figure. S017. ^{195}Pt{1H} NMR spectrum of [Pt(Me$_2$dpb)Cl] measured in DMSO-d$_6$ at RT.

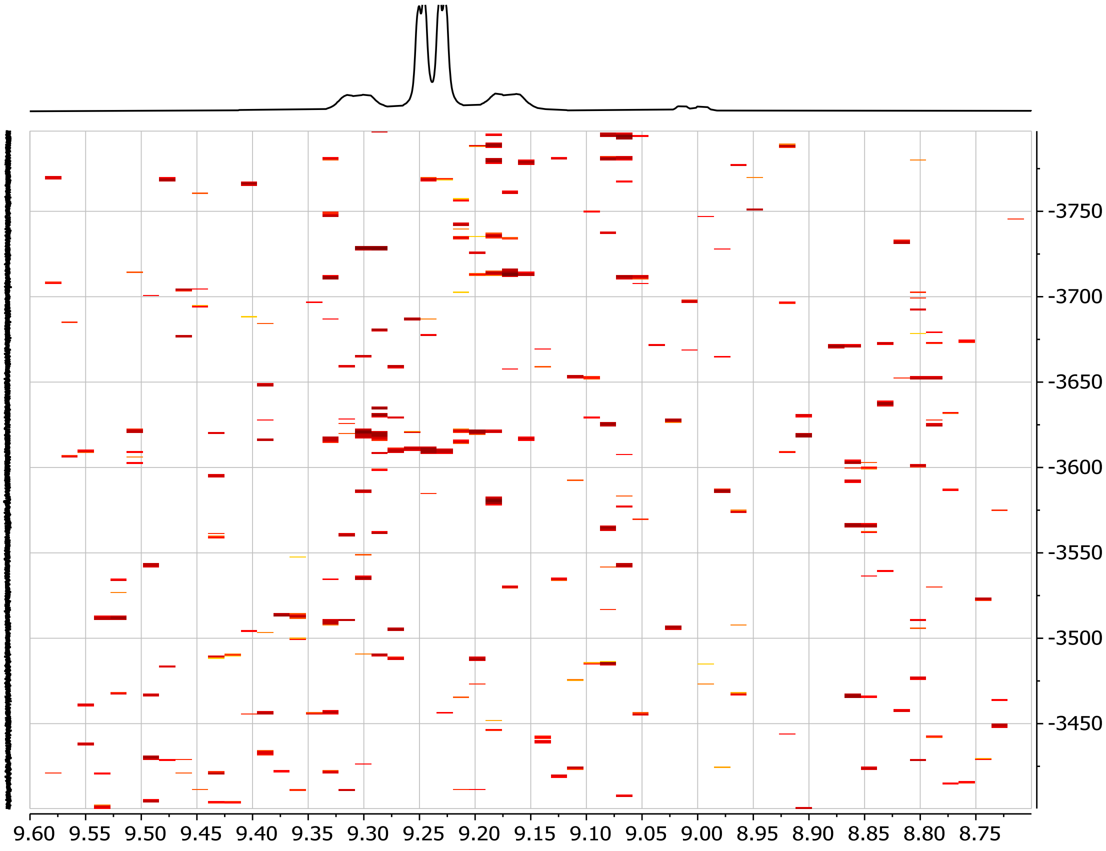

Figure. S018. H,Pt-HMBC spectrum of [Pt(Me$_2$dpb)Cl] measured in DMSO-d$_6$ at RT.

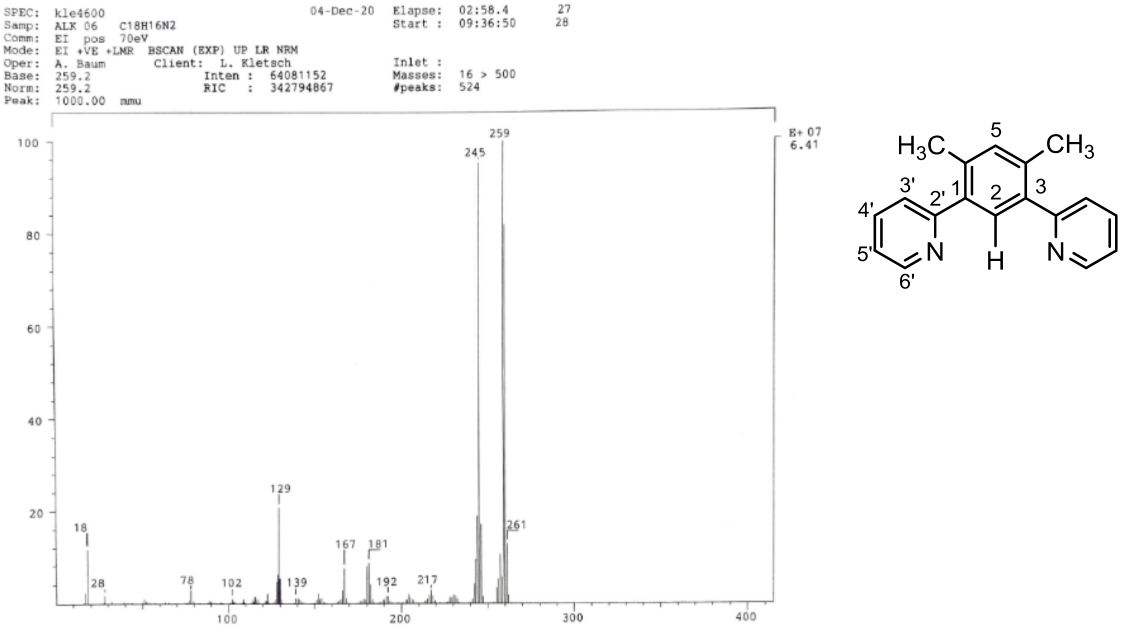

Figure. S019. MS-EI(+) of 1,5-di(2-pyridyl)-2,4-dimethylbenzene (Me_2dpbH).

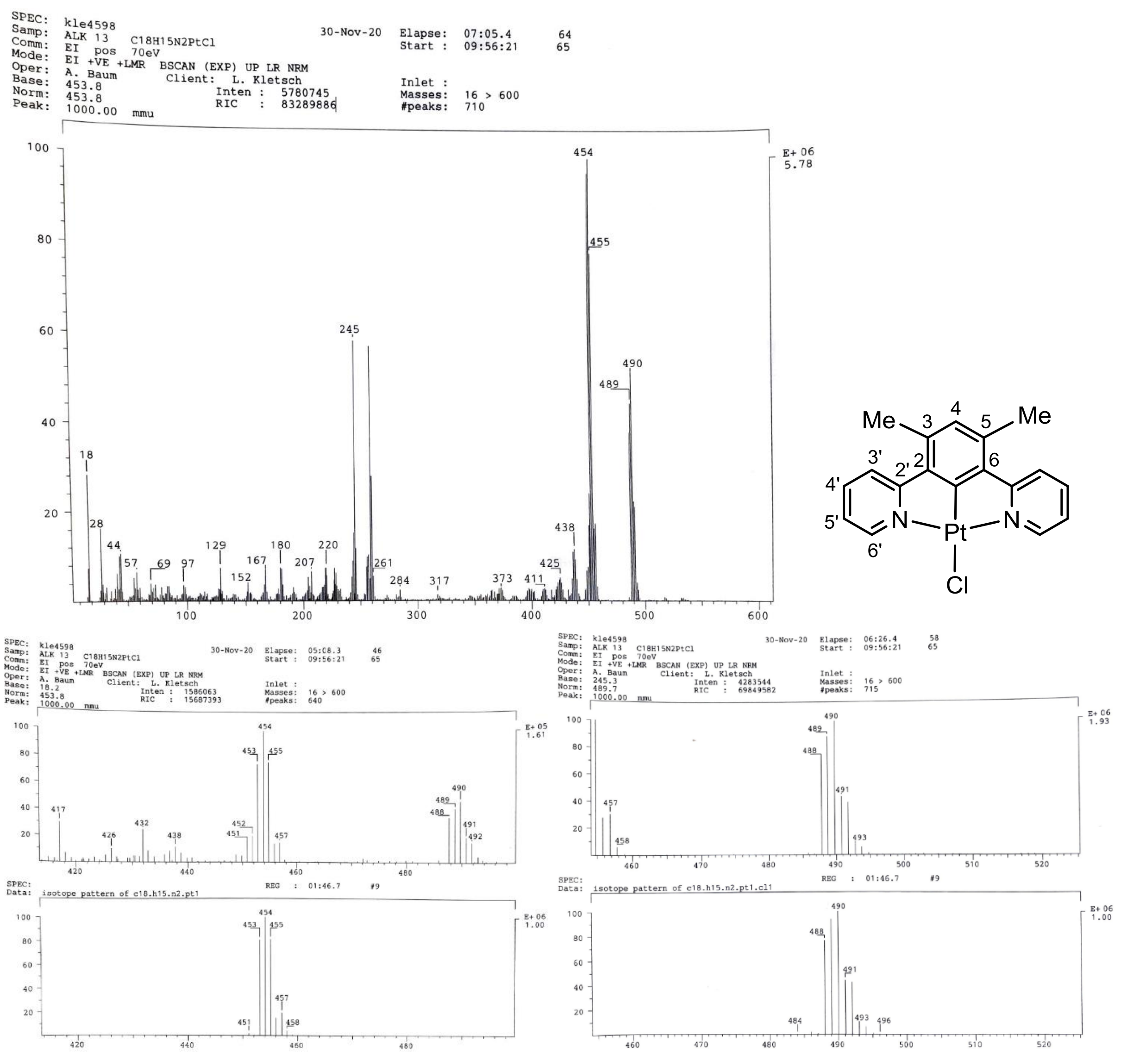

Figure. S020. MS-EI(+) of [Pt(Me_2dpb)Cl].

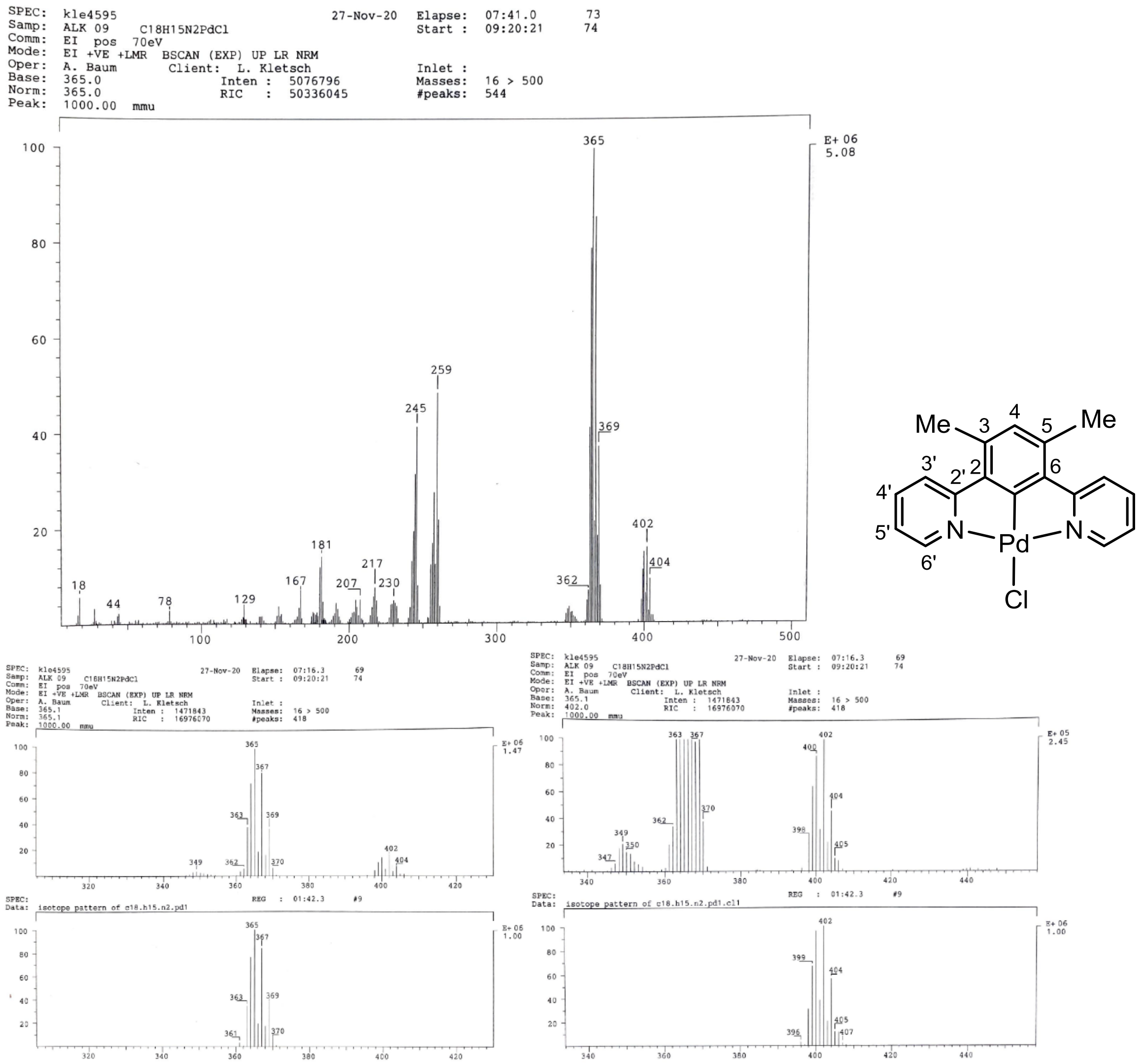

Figure. S021. MS-EI(+) of [Pd(Me2dpb)Cl].

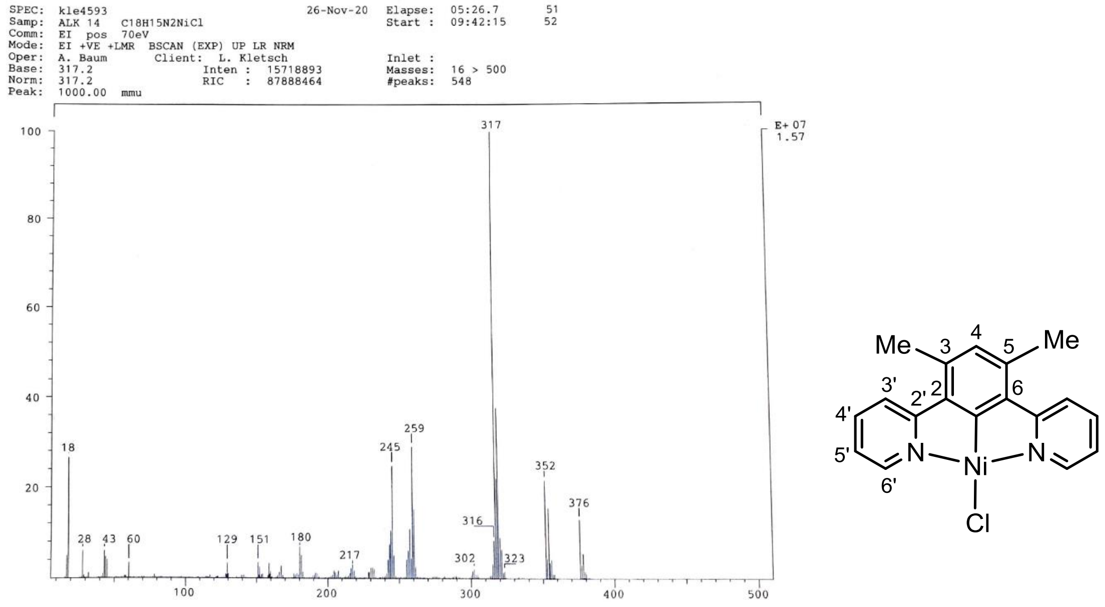

Figure. S022. MS-EI(+) of [Ni(Me2dpb)Cl]

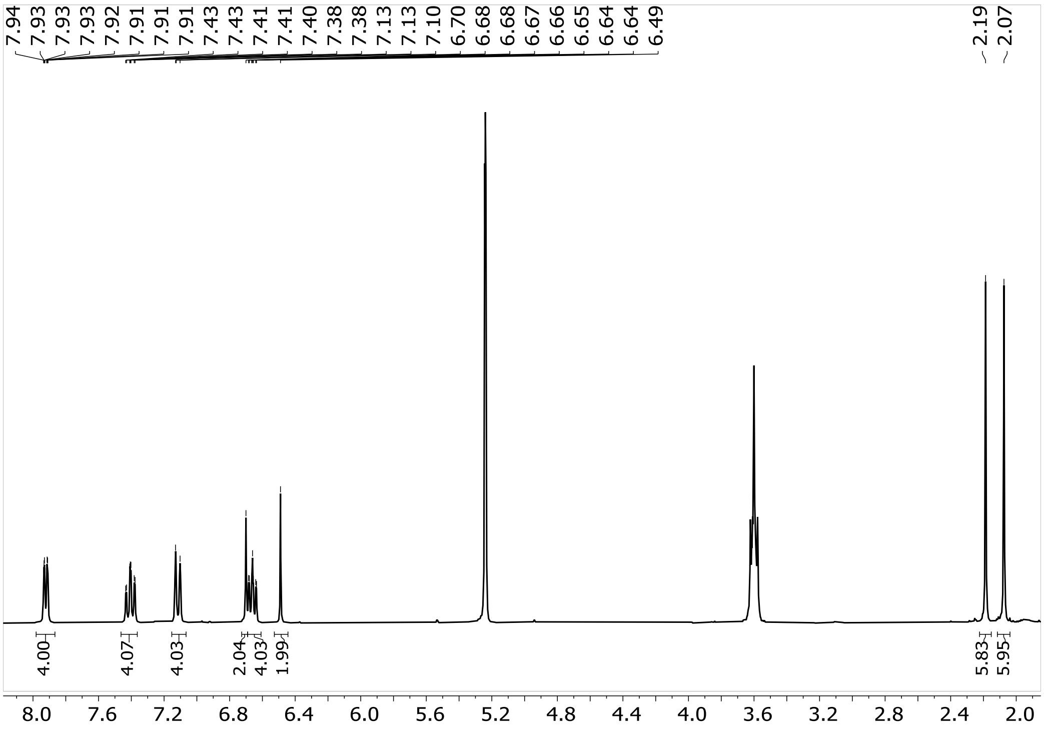

Figure S023. 300 MHz ^{1}H NMR spectrum of [Pd_4(μ-κ^2,κ^{2}dpb)$_2$(μ-κ^1,κ^1-OAc)$_4$] in CD_2Cl_2 (synthetic route using $PdCl_2$).

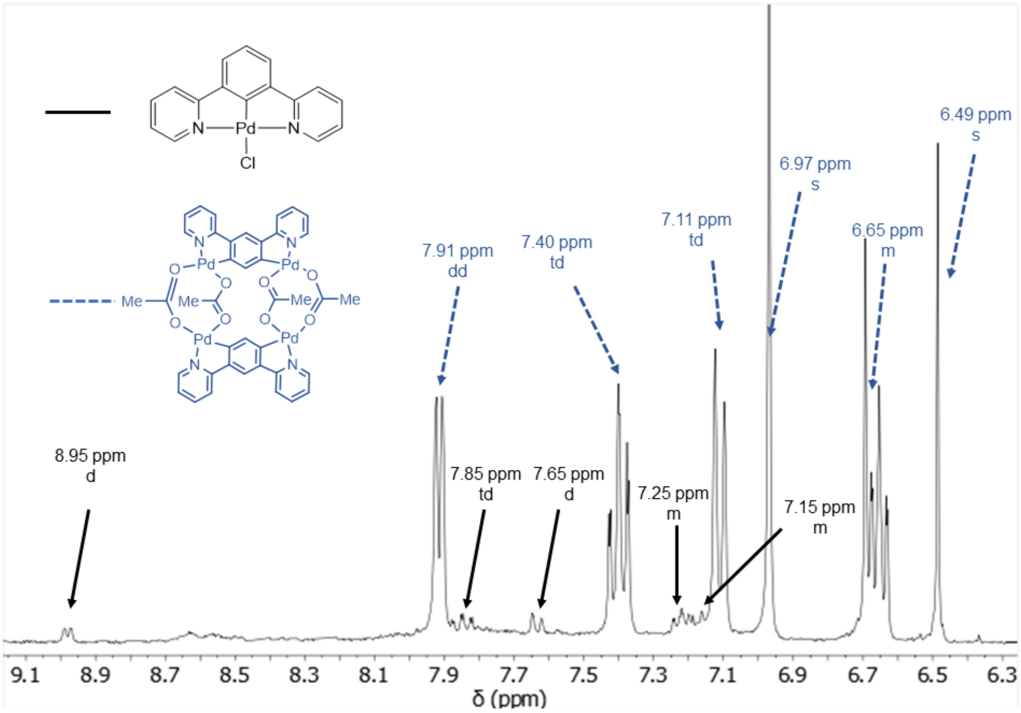

Figure S024. 300 MHz ^{1}H NMR spectrum of [Pd_4(μ-κ^2,κ^{2}dpb)$_2$(μ-κ^1,κ^1-OAc)$_4$] with traces of [Pd(PyPhPy)Cl] in CD_2Cl_2 (synthetic route using [(COD)$PdCl_2$]).

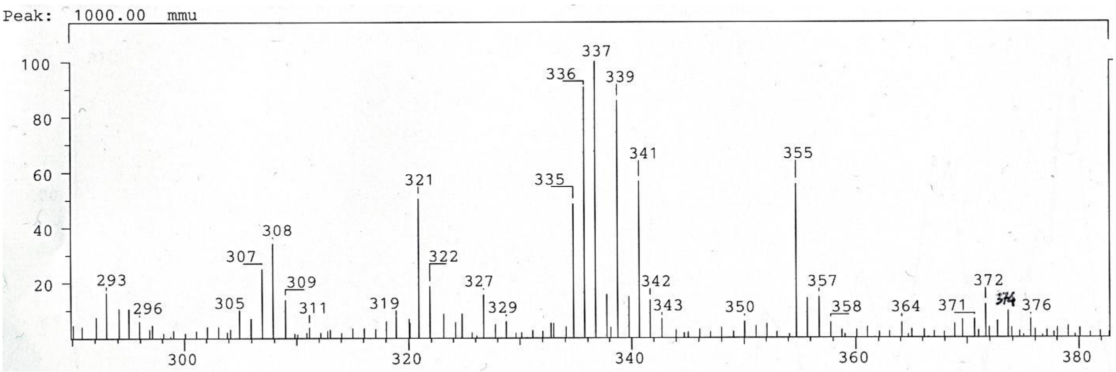

Figure S025. EI-MS(+) of the product mixture of [Pd$_4$(μ-κ^2,κ^{2}dpb)$_2$(μ-κ^1,κ^1-OAc)$_4$] with traces of [Pd(dpb)Cl].

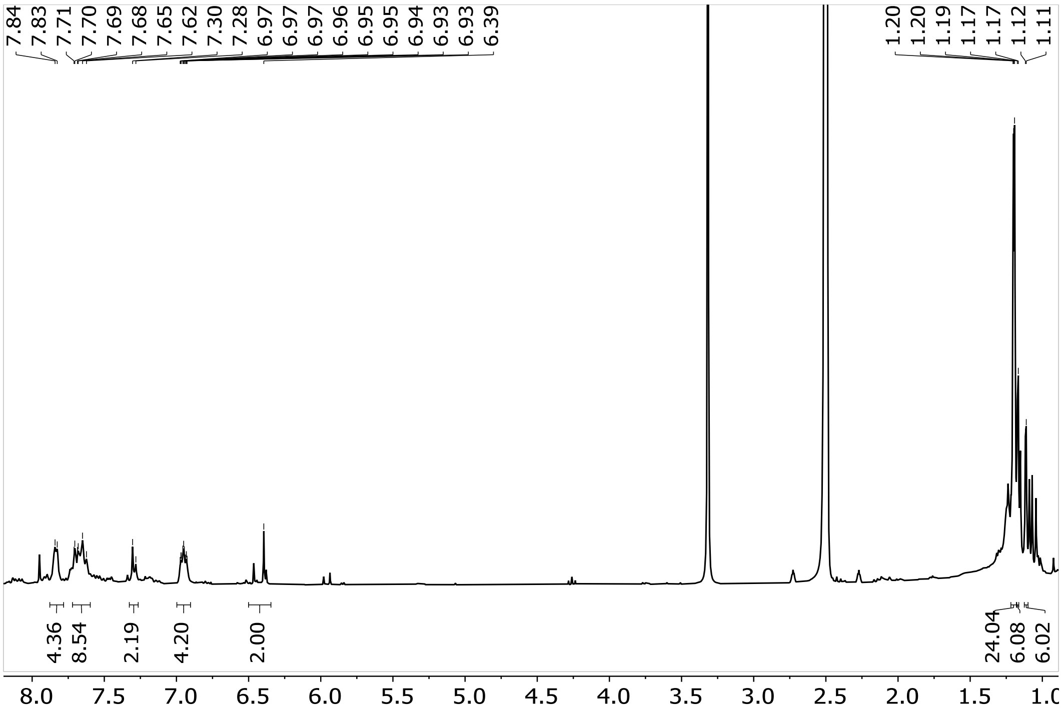

Figure S026. 300 MHz ^{1}H NMR spectrum of [Pd$_4$(μ-κ^2,κ^{2}dpb)$_2$(μ-κ^1,κ^1-OPiv)$_4$] in DMSO-d$_6$.

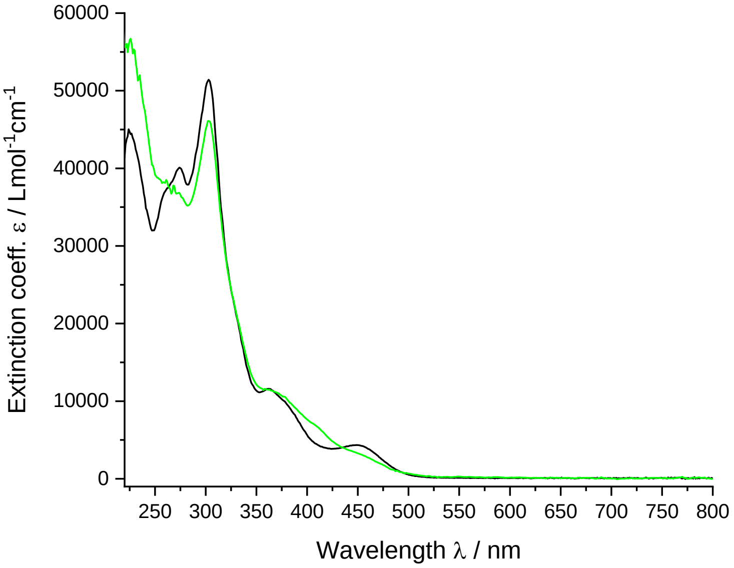

Figure S027. UV-vis absorption spectra of [Pd$_4$(μ-κ^2,κ^{2}dpb)$_2$(μ-κ^1,κ^1-OAc)$_4$] (black) and [Pd$_4$(μ-κ^2,κ^{2}dpb)$_2$(μ-κ^1,κ^1-OPiv)$_4$] (green) in THF.
