# Supplementary material for: Photoluminescence of Ni(II), Pd(II), and Pt(II) Complexes [M(Me2dpb)Cl] Obtained from C‒H Activation of 1,5-Di(2-pyridyl)-2,4-dimethylbenzene (Me2dpbH)
Source: Molecules. 2021 Aug 20;26(16):5051. doi: 10.3390/molecules26165051 (PMC8401505; doi:10.3390/molecules26165051)
Supplement: Supplementary file 1 [file molecules-26-05051-s001.zip › Supplement I Ni Pd Pt Me2DPB Cl.pdf]

Supplementary Materials I
for
Photoluminescence of Ni(II), Pd(II), and Pt(II) complexes [M(Me$_2$dpb)Cl] obtained from C–H activation of 1,5-di(2-pyridyl)-2,4-dimethylbenzene (Me$_2$dpbH)

Contents:

Syntheses
Scheme S1. Overview over attempted syntheses of Pd(II) complexes.
Synthesis of [Pd$_4$(μ-κ^2,κ^{2}dpb)$_2$(μ-κ^1,κ^1-OAc)$_4$] using PdCl$_2$.
Synthesis of [Pd$_4$(μ-κ^2,κ^{2}dpb)$_2$(μ-κ^1,κ^1-OAc)$_4$] using [Pd(COD)Cl$_2$].
Synthesis of [Pd$_4$(μ-κ^2,κ^{2}dpb)$_2$(μ-κ^1,κ^1-OPiv)$_4$] using [Pd(COD)Cl$_2$].

Photophysical characterisation

Supplementary Figures and Tables
Figure S1. Ellipsoid visualisation of the asymmetric unit of [Ni(Me$_2$dpb)Cl]·CH$_2$Cl$_2$ at 50% probability and view on the crystal structure along the crystallographic *b*/*c* axis.
Figure S2. View on the crystal structure of [Ni(Me$_2$dpb)Cl]·CH$_2$Cl$_2$ along the crystallographic *c* axis and close-up of the stacking of the molecules.
Figure S3. View on the crystal structure of [Ni(Me$_2$dpb)Cl]·CH$_2$Cl$_2$ along the crystallographic *b* axis.
Figure S4. Ellipsoid visualisation of the asymmetric unit of [Pd(Me$_2$dpb)Cl] at 50% probability.
Figure S5. View on the crystal structure of [Pd(Me$_2$dpb)Cl] along the crystallographic *a* axis.
Figure S6. View on the crystal structure of [Pd(Me$_2$dpb)Cl] along the crystallographic *b* axis.
Figure S7. Cyclic voltammogramms of Me$_2$dpbH in THF/*n*-Bu$_4$NPF$_6$.
Figure S8. Contour plots and energies of selected DFT-calculated molecular orbitals of [M(Me$_2$dpb)Cl] (M = Ni, Pd, Pt).
Figure S9. TD-DFT-calculated absorptions spectrum of [Pd(Me$_2$dpb)Cl].
Figure S10. UV-vis absorption spectra of [Pd(Me$_2$dpb)Cl] in THF/*n*-Bu$_4$NPF$_6$ recorded during cathodic reduction (A) and anodic oxidation (B).
Figure S11. UV-vis absorption spectra of [Ni(Me$_2$dpb)Cl] in THF/*n*-Bu$_4$NPF$_6$ recorded during cathodic reduction and anodic oxidation.
Figure S12. Normalised photoluminescence excitation and emission spectra of [Pt(dpb)Cl] in DCM and 2-MeTHF at 298 K and in frozen glassy matrices of 2-MeTHF at 77 K.
Figure S13. Normalised photoluminescence excitation and emission spectra of [Pt(Me$_2$dpb)Cl] in DCM and 2-MeTHF at 298 K and in frozen glassy matrices of 2-MeTHF at 77 K.
Figure S14. Normalised photoluminescence excitation and emission spectra of [Pd(Me$_2$dpb)Cl] in frozen glassy matrices of 2-MeTHF at 77 K.
Figure S15. Time-resolved photoluminescence decay of [Pt(dpb)Cl] in air-equilibrated DCM at 298 K, including the residuals (λ_{exc} = 376.7 nm, λ_{em} = 490 nm).
Figure S16. Time-resolved photoluminescence decay of [Pt(dpb)Cl] in deaerated DCM at 298 K, including the residuals (λ_{exc} = 376.7 nm, λ_{em} = 490 nm).
Figure S17. Time-resolved photoluminescence decay of [Pt(dpb)Cl] in air-equilibrated 2-MeTHF at 298 K, including the residuals (λ_{exc} = 376.7 nm, λ_{em} = 490 nm).
Figure S18. Time-resolved photoluminescence decay of [Pt(dpb)Cl] in deaerated 2-MeTHF at 298 K, including the residuals (λ_{exc} = 376.7 nm, λ_{em} = 490 nm).
Figure S19. Time-resolved photoluminescence decay of [Pt(dpb)Cl] in a frozen glassy matrix of 2-MeTHF at 77 K, including the residuals (λ_{exc} = 376.7 nm, λ_{em} = 490 nm).
Figure S20. Time-resolved photoluminescence decay of [Pt(Me$_2$dpb)Cl] in air-equilibrated DCM at 298 K, including the residuals (λ_{exc} = 376.7 nm, λ_{em} = 490 nm).
Figure S21. Time-resolved photoluminescence decay of [Pt(Me$_2$dpb)Cl] in deaerated DCM at 298 K, including the residuals (λ_{exc} = 376.7 nm, λ_{em} = 490 nm).
Figure S22. Time-resolved photoluminescence decay of [Pt(Me$_2$dpb)Cl] in air-equilibrated 2-MeTHF at 298 K, including the residuals (λ_{exc} = 376.7 nm, λ_{em} = 490 nm).
Figure S23. Time-resolved photoluminescence decay of [Pt(Me$_2$dpb)Cl] in deaerated 2-MeTHF at 298 K, including the residuals (λ_{exc} = 376.7 nm, λ_{em} = 490 nm).
Figure S24. Time-resolved photoluminescence decay of [Pt(Me$_2$dpb)Cl] in a frozen glassy matrix of 2-MeTHF at 77 K, including the residuals (λ_{exc} = 376.7 nm, λ_{em} = 485 nm).
Figure S25. Time-resolved photoluminescence decay of [Pd(Me$_2$dpb)Cl] in a frozen glassy matrix of 2-MeTHF at 77 K, including the residuals (λ_{exc} = 376.7 nm, λ_{em} = 470 nm).

Table S1. Crystal data and structure refinement for the [M(Me$_2$dpb)Cl]·compounds.[a]
Table S2. Selected DFT-optimised geometrical data of the complex [Ni(Me$_2$dpb)Cl] compared with experimental data from the crystal structure of [Ni(Me$_2$dpb)Cl]·CH$_2$Cl$_2$.
Table S3. Selected DFT-optimised geometrical data of the complex [Pd(Me$_2$dpb)Cl] compared with experimental data from the crystal structure of [Pd(Me$_2$dpb)Cl]·CH$_2$Cl$_2$.

Table S4. Selected DFT-optimised geometrical data of the complex [Pt(Me_2dpb)Cl] compared with experimental data from the crystal structure of [Pt(Me_2dpb)Cl]·CH_2Cl_2.
Table S5. Selected absorption maxima for Me_2dpbH and the complexes [M(Me_2dpb)Cl] (M = Ni, Pd, Pt).
Table S6. Selected TD-DFT calculated vertical $S_0 \rightarrow S_n$ transitions for [Pt(Me_2dpb)Cl].
Table S7. Selected TD-DFT calculated vertical $S_0 \rightarrow S_n$ transitions for [Pd(Me_2dpb)Cl].
Table S8. Selected TD-DFT calculated vertical $S_0 \rightarrow S_n$ transitions for [Ni(Me_2dpb)Cl].
Table S9. Selected UV-vis absorption maxima of the reduced and oxidised complexes [M(Me_2dpb)Cl]^n.
Table S10. Fractional atomic coordinates (×10^4) and equivalent isotropic displacement parameters (Å^2×10^3) for [Ni(Me_2dpb)Cl]·CH_2Cl_2.
Table S11. Anisotropic displacement parameters (Å^2×10^3) for [Ni(Me_2dpb)Cl]·CH_2Cl_2.
Table S12. Selected bond lengths for [Ni(Me_2dpb)Cl]·CH_2Cl_2.
Table S13. Selected bond angles for [Ni(Me_2dpb)Cl]·CH_2Cl_2.
Table S14. Selected torsion angles for [Ni(Me_2dpb)Cl]·CH_2Cl_2.
Table S15. Hydrogen atom coordinates (Å×10^4) and isotropic displacement parameters (Å^2×10^3) for [Ni(Me_2dpb)Cl]·CH_2Cl_2.
Table S16. Fractional atomic coordinates (×10^4) and equivalent isotropic displacement parameters (Å^2×10^3) for [Pd(Me_2dpb)Cl].
Table S17. Anisotropic displacement parameters (Å^2×10^3) for [Pd(Me_2dpb)Cl].
Table S18. Selected bond lengths for [Pd(Me_2dpb)Cl].
Table S19. Selected bond angles for [Pd(Me_2dpb)Cl].
Table S20. Selected torsion angles for [Pd(Me_2dpb)Cl].
Table S21. Hydrogen atom coordinates (Å×10^4) and isotropic displacement parameters (Å^2×10^3) for [Pd(Me_2dpb)Cl].

References

Syntheses

Overview over the attempted syntheses of [Pd(dpb)Cl] and [Pd(Me_2dpb)Cl]:

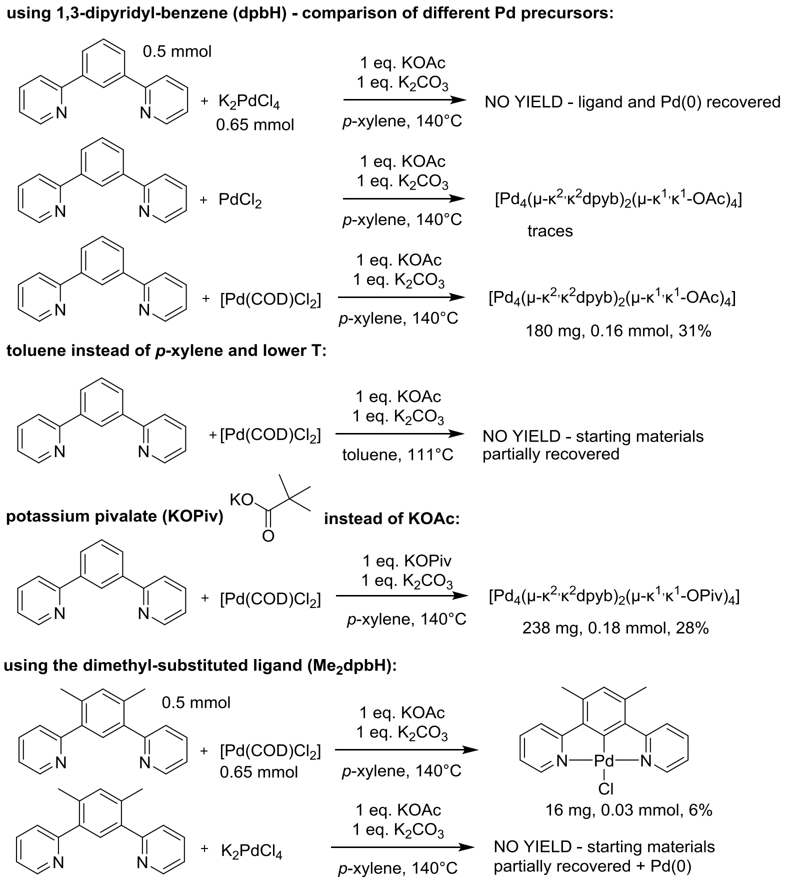

Scheme S1. Overview over attempted syntheses of Pd(II) complexes with the dpbH protoligand and the Me_2dpbH derivative using the base-assisted method.

Attempted synthesis of [Pd(Me_2dpb)Cl]. Solutions of 0.3 mmol Me_2dpbH dissolved in 7.5 mL MeCN and 0.3 mmol $K_2[PdCl_4]$ dissolved in 7.5 mL H_2O were mixed and heated under reflux (~90 °C) for 2 h, as recently described for the synthesis of [Pd(phbpy)Cl] (Hphbpy = 6-phenyl-2,2′-bipyridine [1]. During the reaction, the mixture turned dark.

Filtration gave a light yellow filtrate and a grey-black precipitate. The latter was extracted using CH_2Cl_2 but no product complex was observed in NMR spectra of the obtained grey material. Instead, we found traces of the ligand Me_2dpbH.

Synthesis of [$Pd_4(\mu\text{-}\kappa^2,\kappa^2dpb)_2(\mu\text{-}\kappa^1,\kappa^1\text{-}OAc)_4$] using $PdCl_2$.
In an inert flask with a water trap filled with molecular sieve (3 Å), 0.233 g $PdCl_2$ (1.3 mmol, 1.3 eq.), 0.099 g KOAc (1.0 mmol, 1.0 eq.) and 0.140 g K_2CO_3 (1.0 mmol, 1.0 eq.) were prepared and 0.241 g 1,3-di(2-pyridyl)-benzene (1.0 mmol, 1.0 eq.) and dry *p*-xylene (100 mL) were added and heated under reflux for 72 h. After cooling to ambient temperature, the precipitated black solid was filtered off, washed once with *p*-xylene and the product was extracted using CH_2Cl_2. The solvent was removed and the pale orange solid was dissolved in THF and isolated with a yield of 180 mg (0.16 mmol, 31%). ^{1}H NMR (300 MHz, CD_2Cl_2): δ = 7.92 (ddd, *J* = 5.8, 1.6, 0.7 Hz, 4H), 7.41 (td, *J* = 7.8, 1.6 Hz, 4H), 7.12 (d, *J* = 8.3 Hz, 4H), 6.70 (s, 2H), 6.66 (ddd, *J* = 7.2, 5.7, 1.4 Hz, 4H), 6.49 (s, 2H), 2.19 (s, 6H), 2.07 (s, 6H) ppm.

Synthesis of [$Pd_4(\mu\text{-}\kappa^2,\kappa^2dpb)_2(\mu\text{-}\kappa^1,\kappa^1\text{-}OAc)_4$] using [Pd(COD)$Cl_2$].
In an inert flask with a water trap filled with molecular sieve (3 Å), 0.233 g [Pd(COD)Cl_2] (0.60 mmol, 1.3 eq.), 0.052 g KOAc (0.52 mmol, 1.0 eq.) and 0.072 g K_2CO_3 (0.52 mmol, 1.0 eq.) were prepared and 0.120 g 1,3-di(2-pyridyl)-benzene (0.52 mmol, 1.0 eq.) and dry *p*-xylene (50 mL) were added and heated under reflux for 72 h. After cooling to ambient temperature, the precipitated black solid was filtered off, washed once with *p*-xylene and the product was extracted using CH_2Cl_2. The solvent was removed and the pale orange solid was isolated with a yield of 205 mg (0.18 mmol, 35%). ^{1}H NMR (300 MHz, CD_2Cl_2, signals from [$Pd_4(\mu\text{-}\kappa^2,\kappa^2dpb)_2(\mu\text{-}\kappa^1,\kappa^1\text{-}OAc)_4$]): δ = 7.91 (dd, *J* = 5.7, 0.9 Hz, 2H), 7.40 (td, *J* = 8.1, 1.7 Hz, 2H), 7.11 (d, *J* = 7.8 Hz, 2H), 6.97 (br s, 2H), 6.65 (ddd, *J* = 7.3, 5.7, 1.4 Hz, 1H), 6.49 (s, 1H)., 2.19 (s, 6H), 2.07 (s, 6H) ppm, ^{1}H NMR (300 MHz, CD_2Cl_2, signals from [Pd(PyPhPy)Cl]): δ = 8.95 (d, *J* = 5.4 Hz, 2H), 7.85 (d, *J* = 1.5 Hz, 2H), 7.65 (d, *J* = 8.0 Hz, 2H), 7.25 (m, 4H), 7.15 (m, 2H) ppm. EI-MS(+) m/z = 374 $[Pd(PyPhPy)Cl]^+$ (2%), 337 $[Pd(PyPhPy)]^+$ (6%), 293 $[Pd(PyPh)Cl]^+$, 232 $[PyPhPy]^+$ (100%), 154 $[PyPh]^+$ (16%), 78 $[Py]^+$ (8%).

Synthesis of [$Pd_4(\mu\text{-}\kappa^2,\kappa^2dpb)_2(\mu\text{-}\kappa^1,\kappa^1\text{-}OPiv)_4$] using [Pd(COD)$Cl_2$].
In an inert flask with a water trap filled with molecular sieve (3 Å), 0.186 g [Pd(COD)Cl_2] (0.65 mmol, 1.3 eq.), 0.070 g KOPiv (0.5 mmol, 1.0 eq.) and 0.069 g K_2CO_3 (0.5 mmol, 1.0 eq.) were prepared and 0.116 g 1,3-di(2-pyridyl)-benzene (0.5 mmol, 1.0 eq.) and dry *p*-xylene (150 mL) were added and heated under reflux for 72 h. After cooling to ambient temperature, the precipitated black solid was filtered off, washed once with *p*-xylene and the product was extracted using CH_2Cl_2. The solvent was removed and the pale orange solid was isolated with a yield of 238 mg (0.18 mmol, 28%). ^{1}H NMR (300 MHz, DMSO-d_6): δ = 7.83 (d, *J* = 4.5 Hz, 4H), 7.72-7.60 (m, 9H), 7.29 (d, *J* = 6.8 Hz, 2H), 7.00-6.90 (m, 4H), 6.39 (s, 2H), 1.22-1.18 (m, 24H), 1.17 (d, *J* = 2.0 Hz, 6H), 1.11 (d, *J* = 1.8 Hz, 6H).

Photophysical characterisation

Absorption spectra were measured on a Varian Cary 100 double-beam UV-Vis-NIR spectrometer and baseline-corrected.

Steady-state excitation and emission spectra were recorded on a FluoTime300 spectrometer from PicoQuant equipped with a 300 W ozone-free Xe lamp (250-900 nm), a 10 W Xe flash-lamp (250-900 nm, pulse width < 10 µs) with repetition rates of 0.1 to 300 Hz, an excitation monochromator (Czerny-Turner 2.7 nm/mm dispersion, 1200 grooves/mm, blazed at 300 nm), diode lasers (pulse width < 80 ps) operated by a computer-controlled laser driver PDL-820 (repetition rate up to 80 MHz, burst mode for slow and weak decays), two emission monochromators (Czerny-Turner, selectable gratings blazed at 500 nm with 2.7 nm/mm dispersion and 1200 grooves/mm, or blazed at 1250 nm with 5.4 nm/mm dispersion and 600 grooves/mm), Glan-Thompson polarisers for excitation (Xe-lamps) and emission, a Peltier-thermostatised sample holder from Quantum Northwest (–40 °C to 105 °C), and two detectors, a PMA Hybrid 40 (transit time spread FWHM < 120 ps, 300 to 720 nm) and a R5509-42 NIR-photomultiplier tube (transit time spread FWHM 1.5 ns, 300-1400 nm) with external cooling (–80 °C) from Hamamatsu.

Steady-state and fluorescence lifetimes were recorded in TCSPC mode by a PicoHarp 300 (minimum base resolution 4 ps).

Phosphorescence lifetimes were recorded in MCS mode by a TimeHarp 300 (minimum base resolution 250 ps). Emission and excitation spectra were corrected for source intensity (lamp and grating) by standard correction curves.

The solid sample of [Pd(na(tbppy)tz)Cl] at room temperature was measured on a different FluoTime300 spectrometer from PicoQuant, equipment with a front-face module and a 300 W ozone-free Xe lamp (250-900 nm), a 10 W Xe flash-lamp (250-900 nm, pulse width < 10 µs) with repetition rates of 0.1 to 300 Hz, two excitation monochromators (Czerny-Turner 2.7 nm/mm dispersion, 1200 grooves/mm, blazed at 350 nm and 600 g/mm, blazed at 1250 nm), diode lasers (pulse width < 80 ps) operated by a computer-controlled laser driver PDL-820 (repetition rate up to 80 MHz, burst mode for slow and weak decays), two emission monochromators (Czerny-Turner, selectable gratings blazed at 500 nm with 2.7 nm/mm dispersion and 1200 grooves/mm, or blazed at 1250 nm with 5.4 nm/mm dispersion and 600 grooves/mm) with adjustable slit width between 0 mm and 10 mm, Glan-Thompson polarisers for excitation (Xe-lamps) and emission, a Peltier-thermostatised sample holder (–40 °C to 105 °C), and two detectors, namely a PMA Hybrid 40 (transit time

spread FWHM < 120 ps, 200 to 900 nm) and a R5509-42 NIR-photomultiplier tube (transit time spread FWHM 1.5 ns, 300-1400 nm) from Hamamatsu. Signal-to-noise ratio (optical noise) typically better than 29000:1, as measured with double monochromators in the excitation and emission light path.

Steady-state and fluorescence lifetimes were recorded in TCSPC mode by a PicoHarp 300 (minimum base resolution 4 ps) or MSC mode by a Timeharp 300, where up to several ms can be detected.

Lifetime analysis was performed using the commercial FluoFit software. The quality of the fit was assessed by minimizing the reduced chi squared function (χ^2) and visual inspection of the weighted residuals and their autocorrelation.

Luminescence quantum yields (PLQY) were measured with a Hamamatsu Photonics absolute PL quantum yield measurement system (C9920-02) equipped with a L9799-01 CW Xenon light source (150 W), monochromator, C7473 photonic multi-channel analyser, integrating sphere and employing U6039-05 PLQY measurement software (Hamamatsu Photonics, Ltd., Shizuoka, Japan).

All solvents used were of spectrometric grade (Uvasol®).

Supplementary Figures and Tables

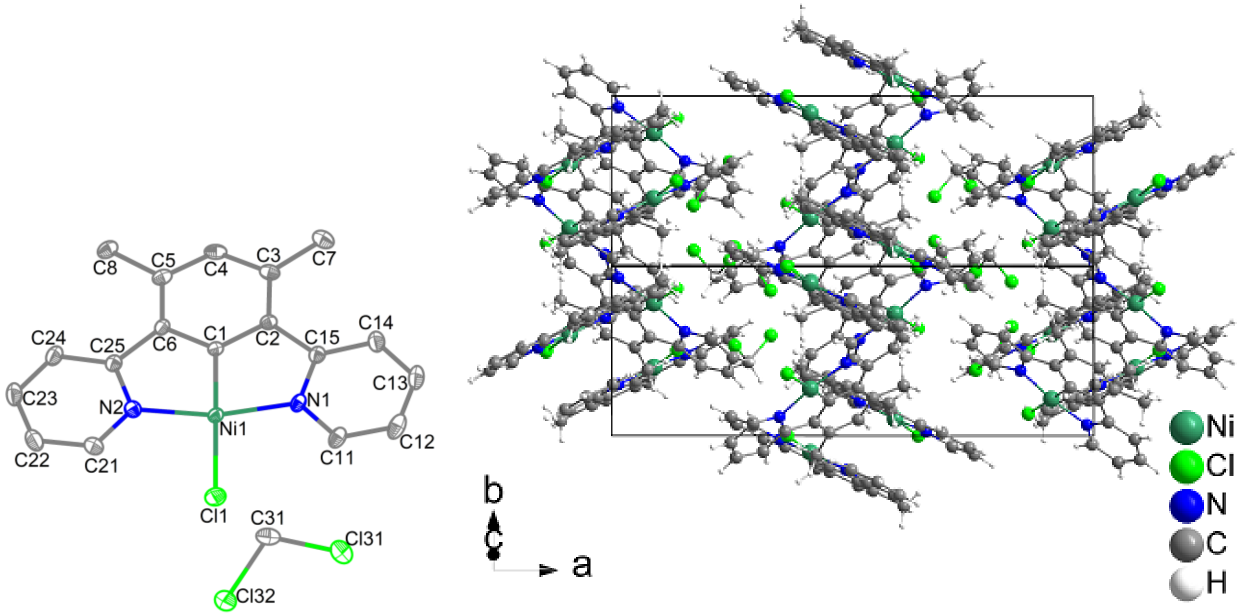

Figure S1. Ellipsoid visualisation of the asymmetric unit of [Ni(Me$_2$dpb)Cl]·CH$_2$Cl$_2$ at 50% probability. Hydrogen atoms are omitted for clarity (left) and view on the crystal structure along the crystallographic *b* /*c* axis.

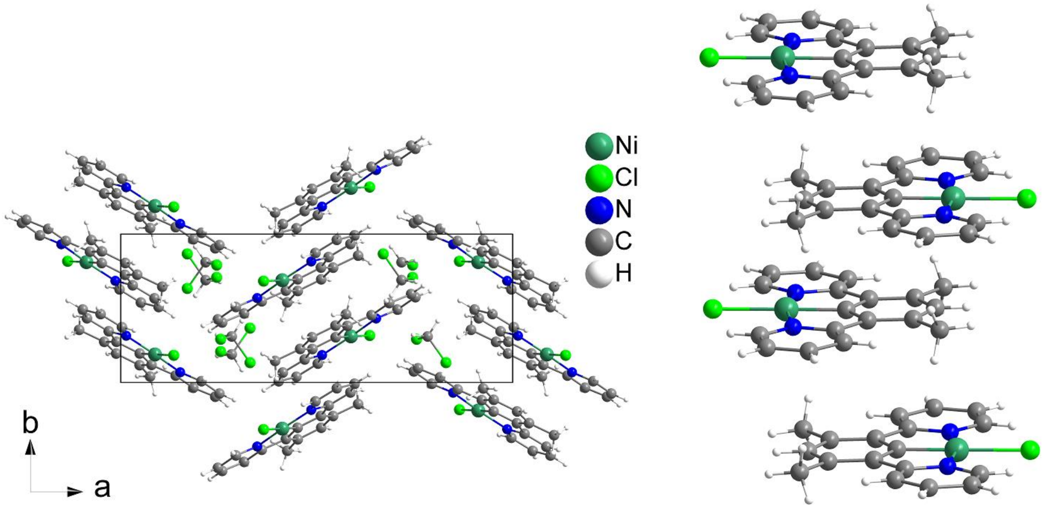

Figure S2. View on the crystal structure of [Ni(Me$_2$dpb)Cl]·CH$_2$Cl$_2$ along the crystallographic *c* axis (left) and close-up of the stacking of the molecules (right).

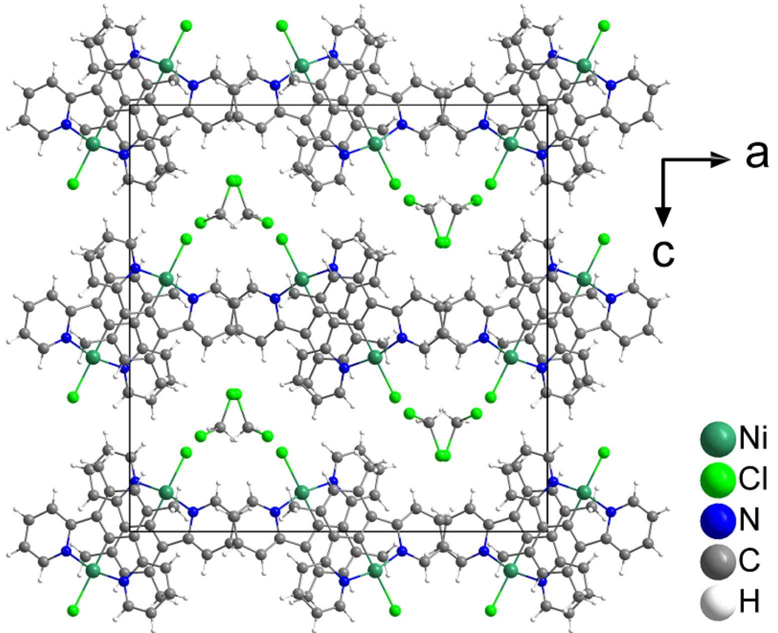

Figure S3. View on the crystal structure of [Ni(Me_2dpb)Cl]·CH_2Cl_2 along the crystallographic *b* axis.

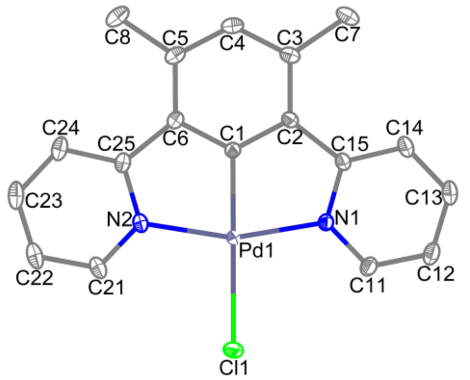

Figure S4. Ellipsoid visualisation of the asymmetric unit of [Pd(Me_2dpb)Cl] at 50% probability. Hydrogen atoms are omitted for clarity.

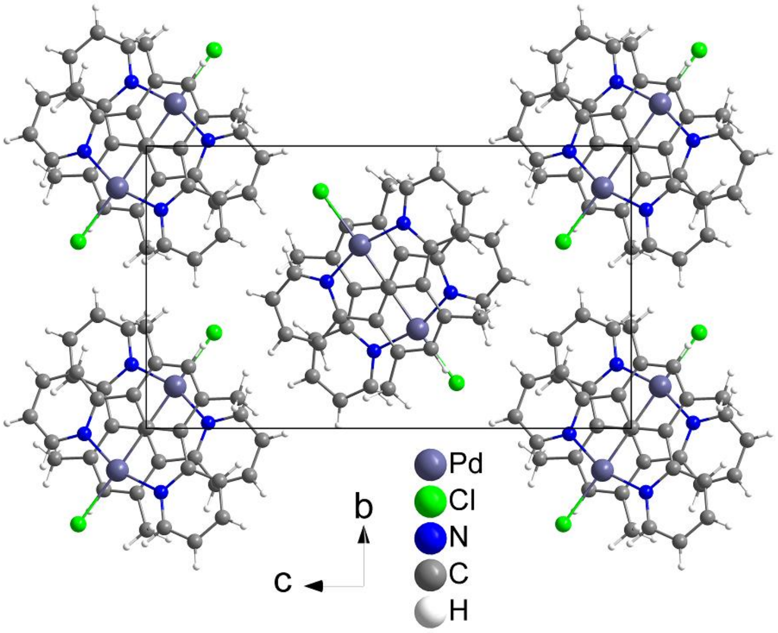

Figure S5. View on the crystal structure of [Pd(Me_2dpb)Cl] along the crystallographic *a* axis.

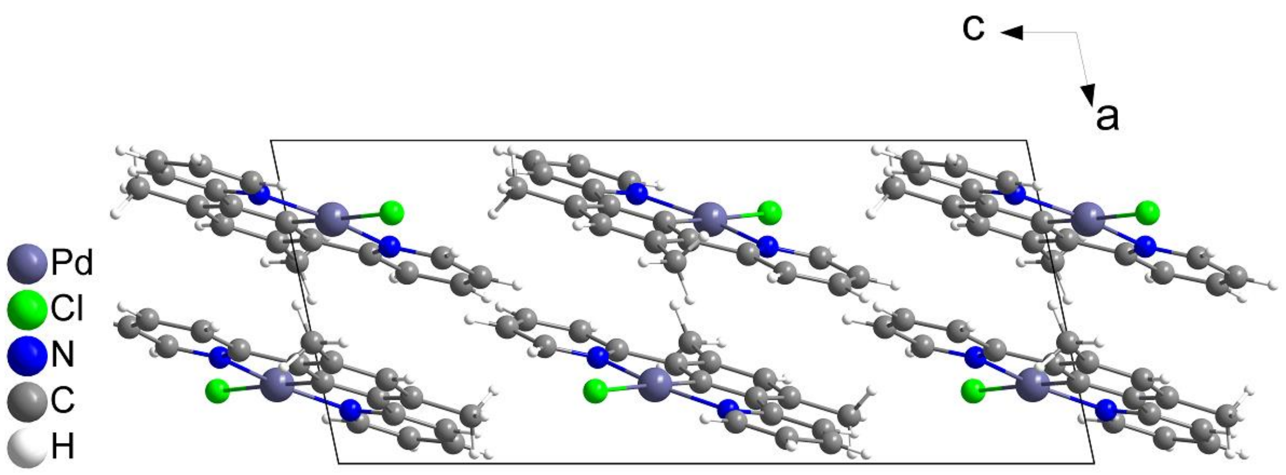

Figure S6. View on the crystal structure of [Pd(Me_2dpb)Cl] along the crystallographic *b* axis.

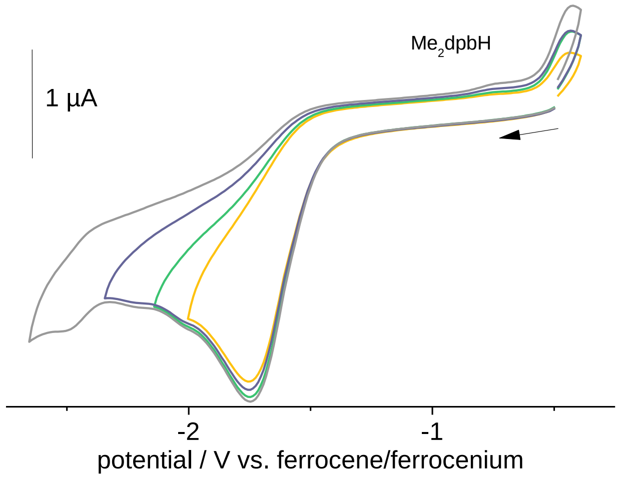

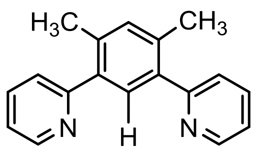

Figure S7. Cyclic voltammogramms of Me_2dpbH in THF/*n*-Bu_4NPF_6.

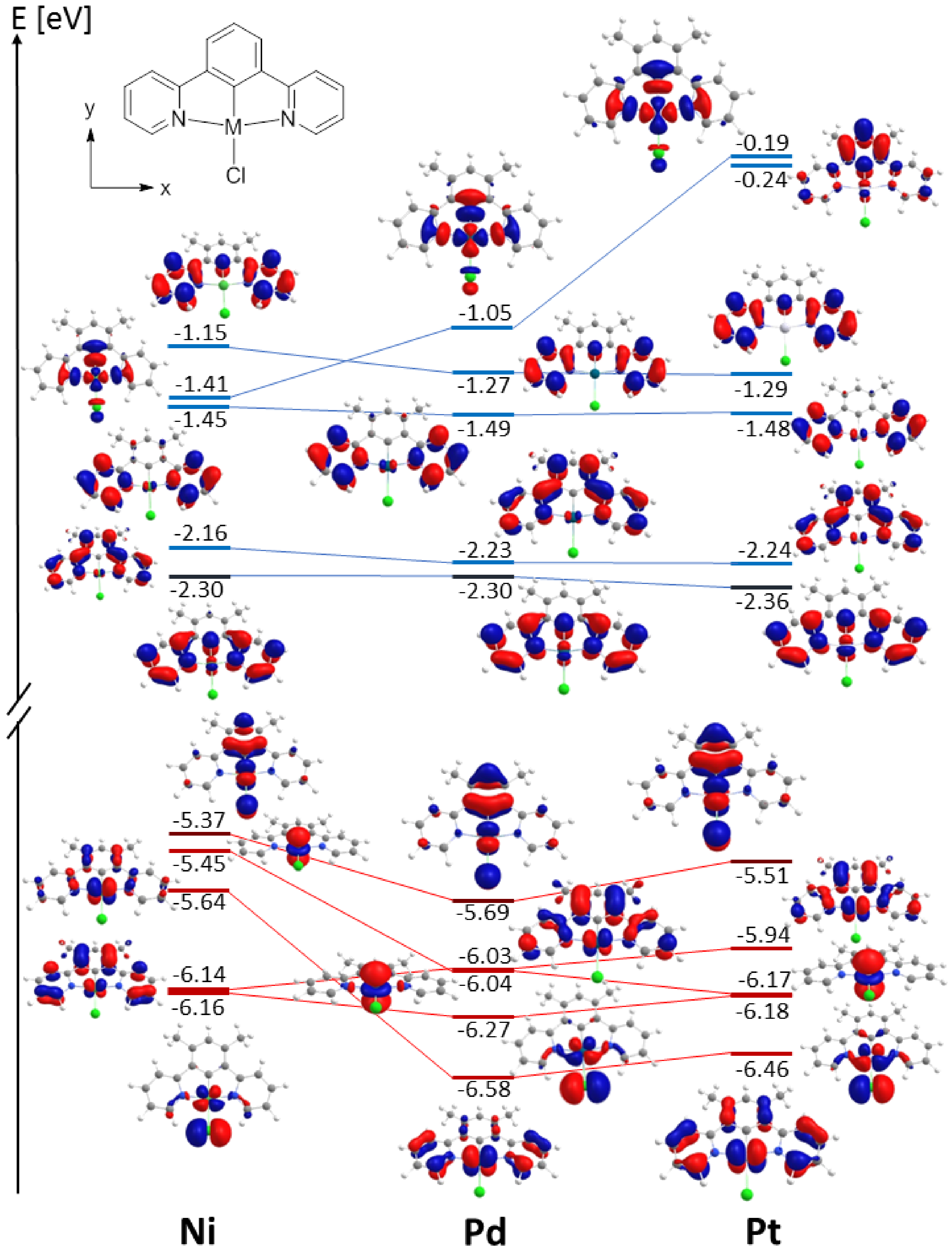

Figure S8. Contour plots and energies of selected DFT-calculated molecular orbitals of [M(Me2dpb)Cl] (M = Ni, Pd, Pt). Calculated at the TPSSh/def2-TZVP level of theory and plotted at an isovalue of 0.04.

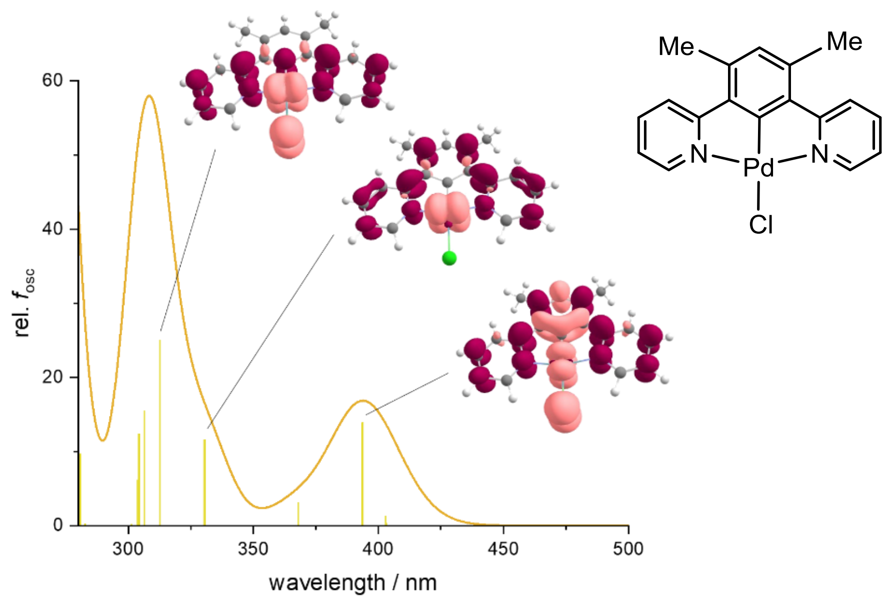

Figure S9. TD-DFT-calculated absorptions spectrum of [Pd(Me_2dpb)Cl]. Calculated at the TPSSh/def2-TZVP level of theory.

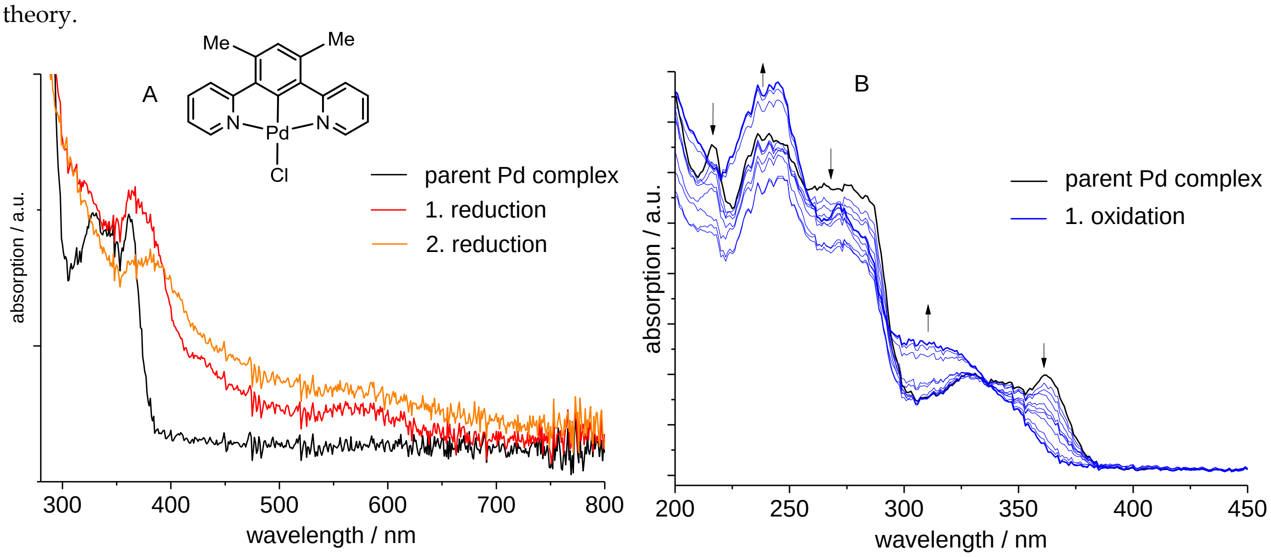

Figure S10. UV-vis absorption spectra of [Pd(Me_2dpb)Cl] in THF/n-Bu_4NPF_6 recorded during cathodic reduction (A) and anodic oxidation (B).

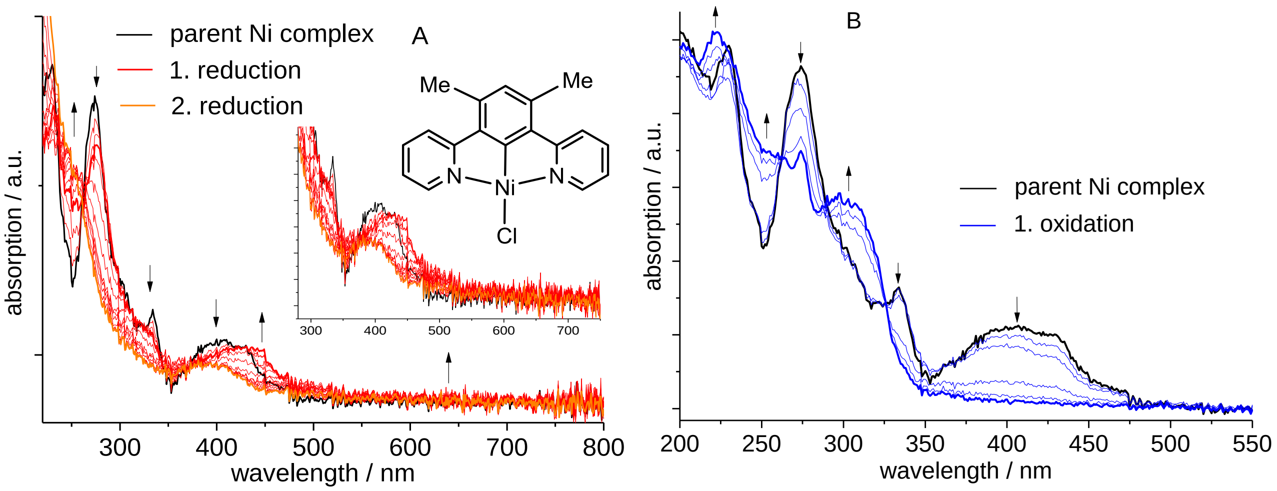

Figure S11. UV-vis absorption spectra of [Ni(Me_2dpb)Cl] in THF/n-Bu_4NPF_6 recorded during cathodic reduction (A) and anodic oxidation (B).

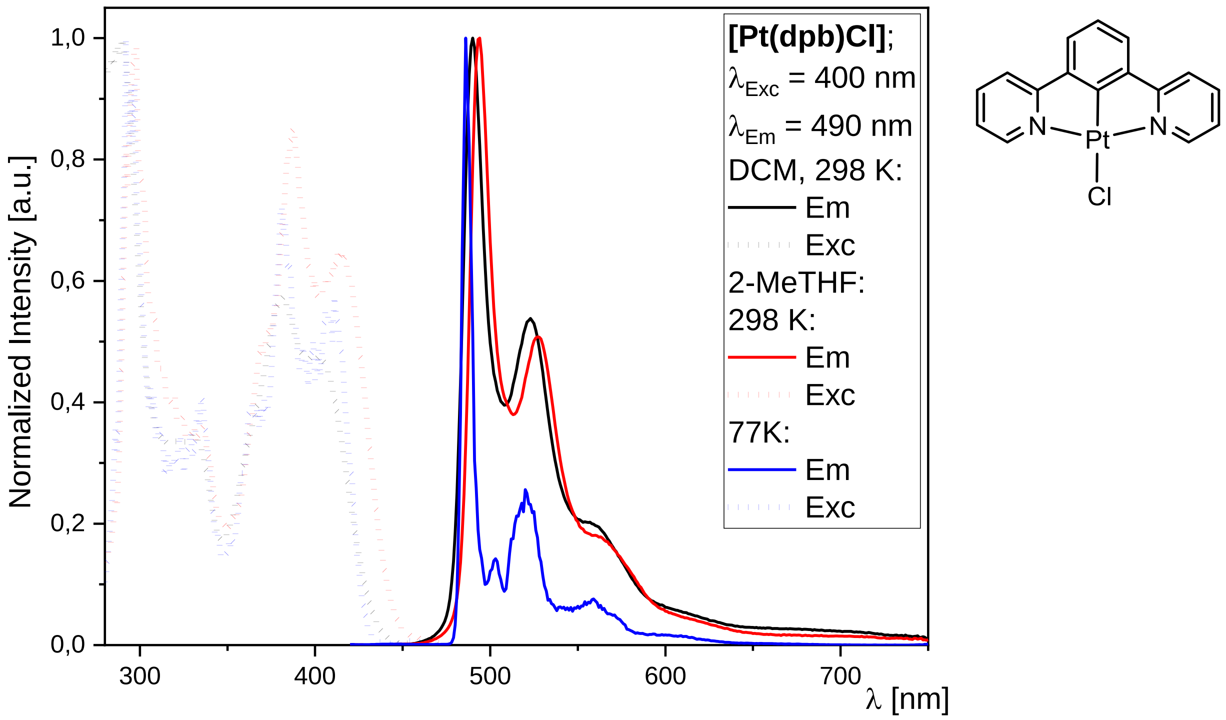

Figure S12. Normalised photoluminescence excitation (Exc, dotted line) and emission spectra (Em, solid line) of **[Pt(dpb)Cl]** in DCM (black) and 2-MeTHF (red) at 298 K and in frozen glassy matrices of 2-MeTHF at 77 K (blue). All solutions were optically diluted (A < 0.1).

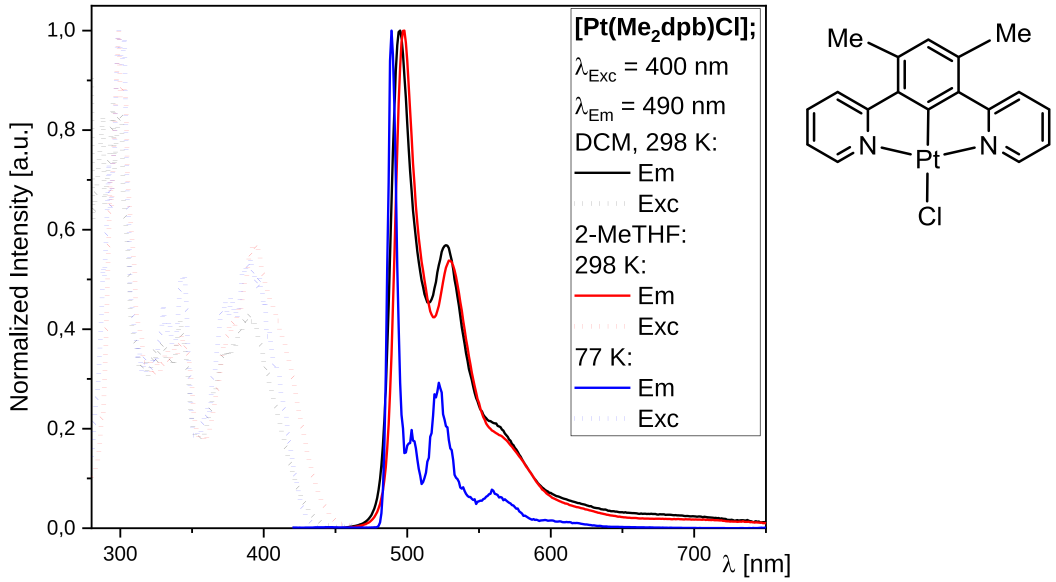

Figure S13. Normalised photoluminescence excitation (Exc, dotted line) and emission spectra (Em, solid line) of **[Pt(Me$_2$dpb)Cl]** in DCM (black) and 2-MeTHF (red) at 298 K and in frozen glassy matrices of 2-MeTHF at 77 K (blue). All solutions were optically diluted (A < 0.1).

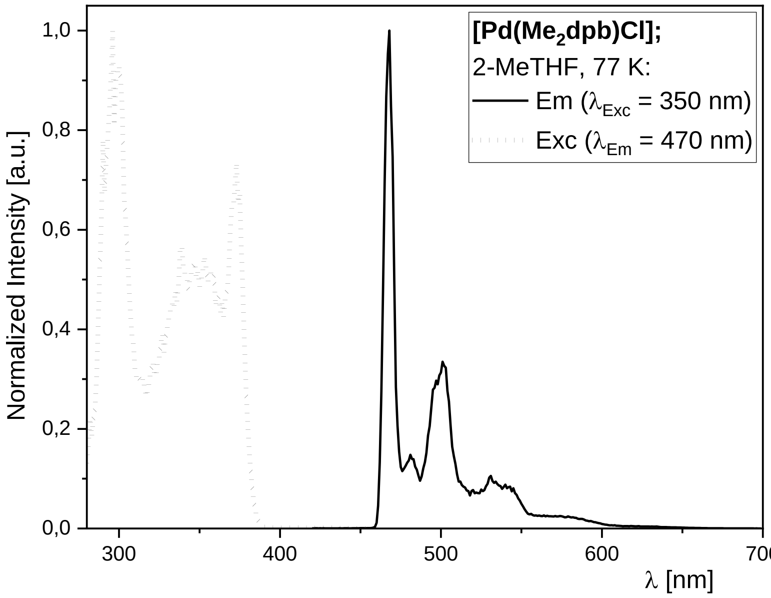

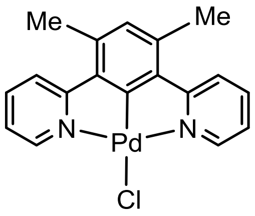

Figure S14. Normalised photoluminescence excitation (Exc, dotted line) and emission spectra (Em, solid line) of **[Pd(Me_2dpb)Cl]** in frozen glassy matrices of 2-MeTHF at 77 K (black). All solutions were optically diluted (A < 0.1).

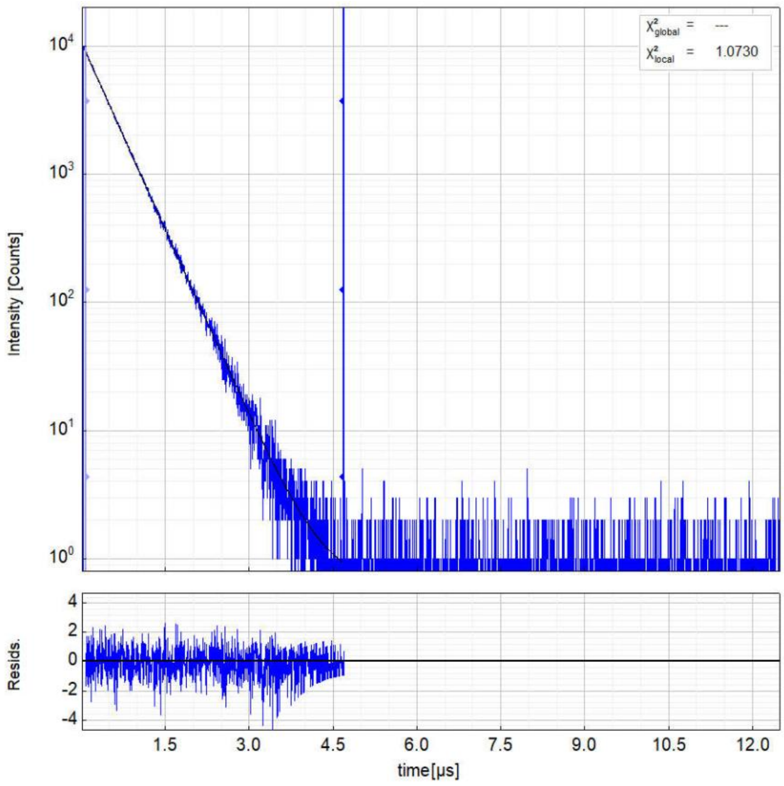

Parameter	Value	Error
A_1[kCnts/Chnl]	8.892	± 0.029
τ_1[ns]	446.43	± 0.75
I_1[kCnts]	992.4	± 1.8
$A_{Rel\,1}$[%]	100.0000	± 0.0000
$I_{Rel\,1}$[%]	100.0000	± 0.0000
$Bkgr_{Dec}$[kCnts]	0.0006	± 0.0002
$\tau_{Av\,Int}$[ns]	446.43	± 0.75
$\tau_{Av\,Amp}$[ns]	446.43	± 0.75

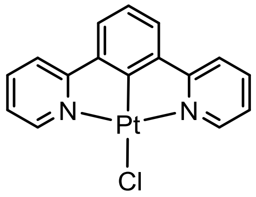

Figure S15. Left: Time-resolved photoluminescence decay of **[Pt(dpb)Cl]** in air-equilibrated DCM at 298 K, including the residuals (λ_{exc} = 376.7 nm, λ_{em} = 490 nm). **Right:** Fitting parameters including pre-exponential factors and confidence limits.

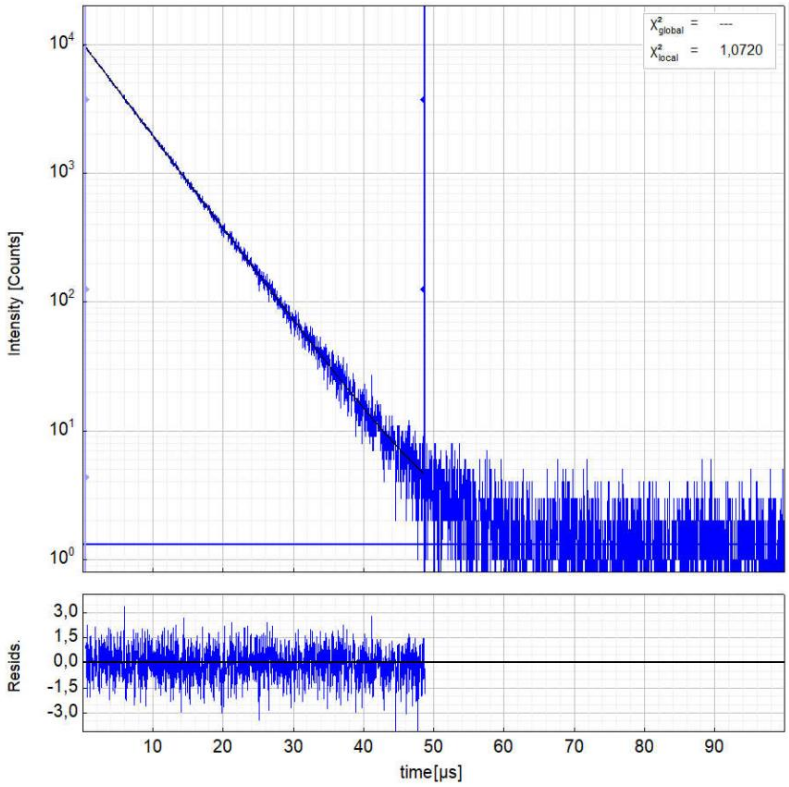

Parameter	Value	Error
A_1[kCnts/Chnl]	9,451	± 0,020
τ_1[ns]	6049,7	± 9,3
I_1[kCnts]	1786,6	± 4,0
$A_{Rel\ 1}$[%]	100,0000	± 0,0000
$I_{Rel\ 1}$[%]	100,0000	± 0,0000
$Bkgr_{Dec}$[kCnts]	0,0013	± 0,0002
$\tau_{Av\,Int}$[ns]	6049,7	± 9,3
$\tau_{Av\,Amp}$[ns]	6049,7	± 9,3

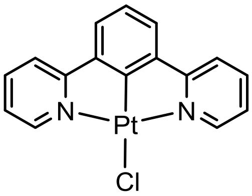

Figure S16. Left: Time-resolved photoluminescence decay of **[Pt(dpb)Cl]** in deaerated DCM at 298 K, including the residuals (λ_{exc} = 376.7 nm, λ_{em} = 490 nm). **Right:** Fitting parameters including pre-exponential factors and confidence limits.

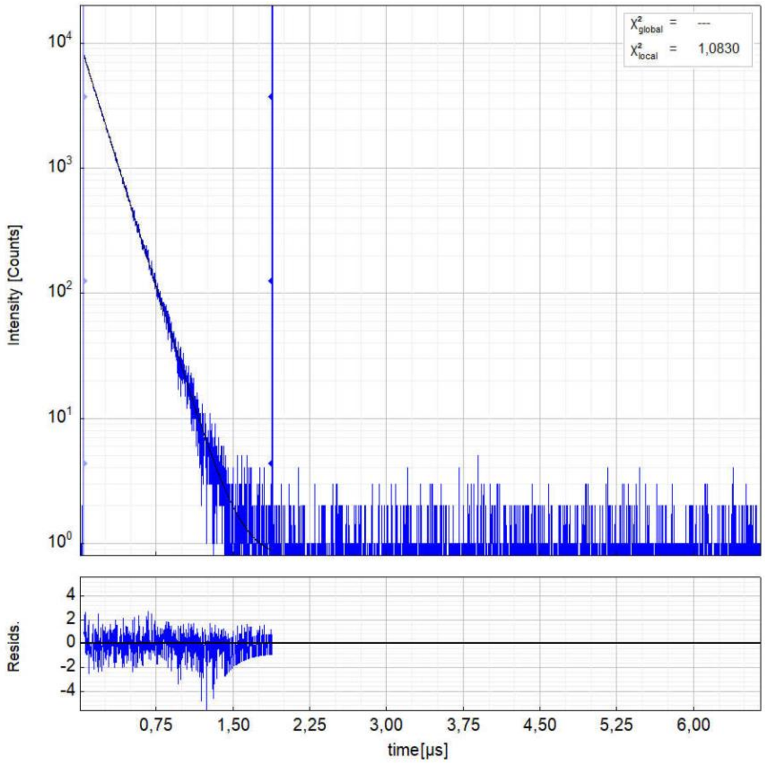

Parameter	Value	Error
A_1[kCnts/Chnl]	7,910	± 0,026
τ_1[ns]	165,90	± 0,32
I_1[kCnts]	656,2	± 1,5
$A_{Rel\ 1}$[%]	100,0000	± 0,0000
$I_{Rel\ 1}$[%]	100,0000	± 0,0000
$Bkgr_{Dec}$[kCnts]	0,0008	± 0,0002
$\tau_{Av\,Int}$[ns]	165,90	± 0,32
$\tau_{Av\,Amp}$[ns]	165,90	± 0,32

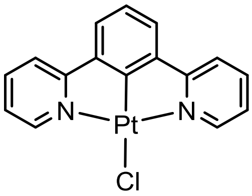

Figure S17. Left: Time-resolved photoluminescence decay of **[Pt(dpb)Cl]** in air-equilibrated 2-MeTHF at 298 K, including the residuals (λ_{exc} = 376.7 nm, λ_{em} = 490 nm). **Right:** Fitting parameters including pre-exponential factors and confidence limits.

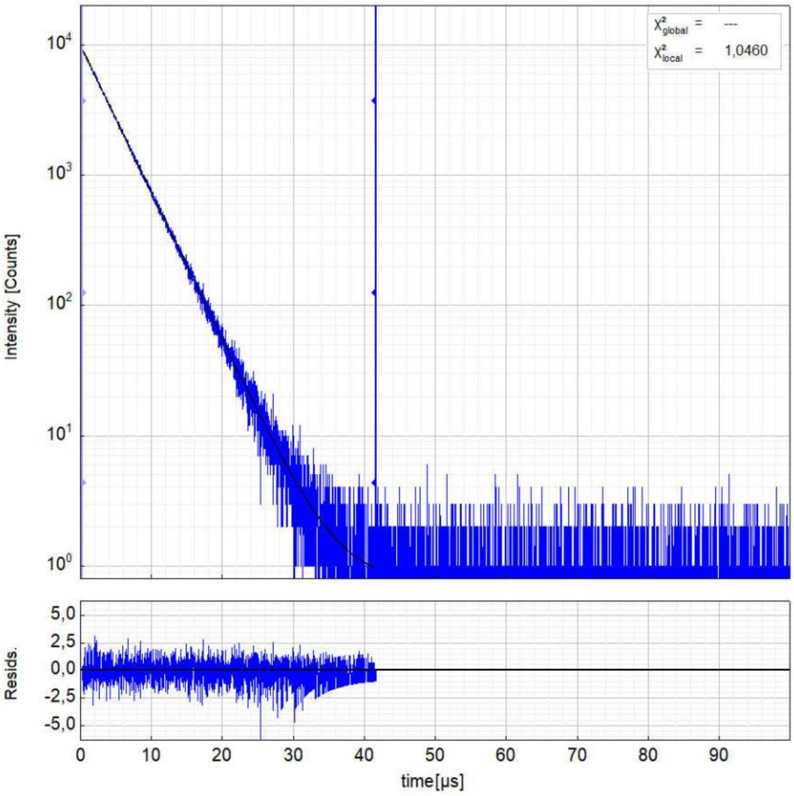

Parameter	Value	Error
A_1[kCnts/Chnl]	9,277	± 0,019
τ_1[ns]	3841,9	± 4,4
I_1[kCnts]	2227,6	± 3,1
$A_{Rel\ 1}$[%]	100,0000	± 0,0000
$I_{Rel\ 1}$[%]	100,0000	± 0,0000
$Bkgr_{Dec}$[kCnts]	0,0008	± 0,0002
$\tau_{Av\ Int}$[ns]	3841,9	± 4,4
$\tau_{Av\ Amp}$[ns]	3841,9	± 4,4

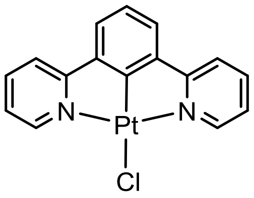

Figure S18. Left: Time-resolved photoluminescence decay of **[Pt(dpb)Cl]** in deaerated 2-MeTHF at 298 K, including the residuals (λ_{exc} = 376.7 nm, λ_{em} = 490 nm). **Right:** Fitting parameters including pre-exponential factors and confidence limits.

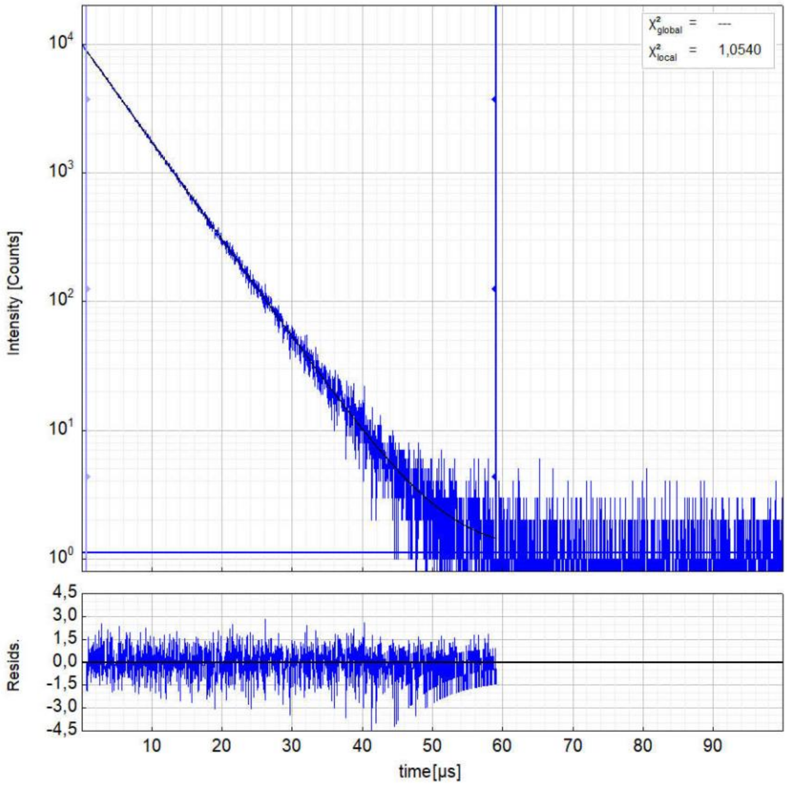

Parameter	Value	Error
A_1[kCnts/Chnl]	8,946	± 0,013
τ_1[ns]	5717,0	± 8,0
I_1[kCnts]	1598,3	± 2,2
$A_{Rel\ 1}$[%]	100,0000	± 0,0000
$I_{Rel\ 1}$[%]	100,0000	± 0,0000
$Bkgr_{Dec}$[kCnts]	0,0011	± 0,0002
$\tau_{Av\ Int}$[ns]	5717,0	± 8,0
$\tau_{Av\ Amp}$[ns]	5717,0	± 8,0

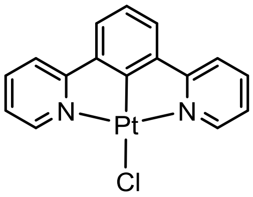

Figure S19. Left: Time-resolved photoluminescence decay of **[Pt(dpb)Cl]** in a frozen glassy matrix of 2-MeTHF at 77 K, including the residuals (λ_{exc} = 376.7 nm, λ_{em} = 490 nm). **Right:** Fitting parameters including pre-exponential factors and confidence limits.

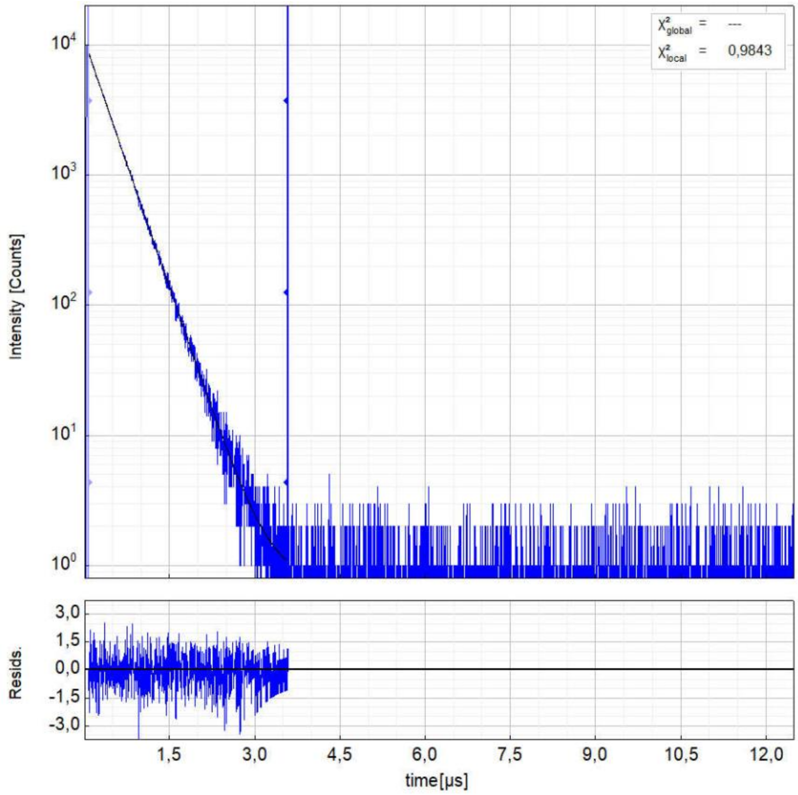

Parameter	Value	Error
A_1[kCnts/Chnl]	8,675	± 0,024
τ_1[ns]	342,80	± 0,77
I_1[kCnts]	743,4	± 1,9
$A_{Rel\,1}$[%]	100,0000	± 0,0000
$I_{Rel\,1}$[%]	100,0000	± 0,0000
$Bkgr_{Dec}$[kCnts]	0,0008	± 0,0003
$\tau_{Av\,Int}$[ns]	342,80	± 0,77
$\tau_{Av\,Amp}$[ns]	342,80	± 0,77

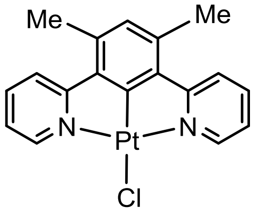

Figure S20. Left: Time-resolved photoluminescence decay of **[Pt(Me2dpb)Cl]** in air-equilibrated DCM at 298 K, including the residuals (λ_{exc} = 376.7 nm, λ_{em} = 490 nm). **Right:** Fitting parameters including pre-exponential factors and confidence limits.

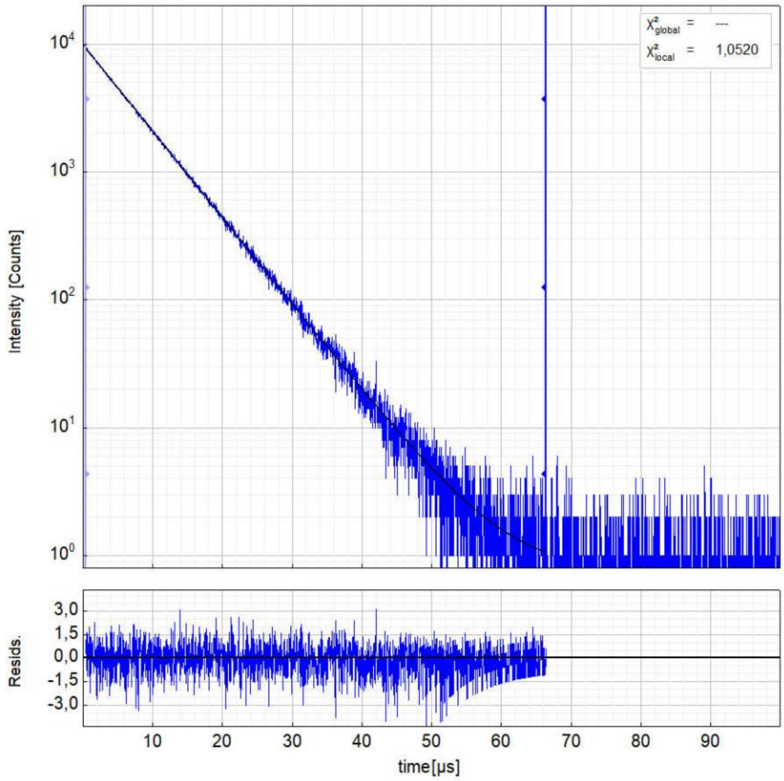

Parameter	Value	Error
A_1[kCnts/Chnl]	9,215	± 0,017
τ_1[ns]	6419,6	± 8,8
I_1[kCnts]	1848,7	± 3,8
$A_{Rel\,1}$[%]	100,0000	± 0,0000
$I_{Rel\,1}$[%]	100,0000	± 0,0000
$Bkgr_{Dec}$[kCnts]	0,0007	± 0,0003
$\tau_{Av\,Int}$[ns]	6419,6	± 8,8
$\tau_{Av\,Amp}$[ns]	6419,6	± 8,8

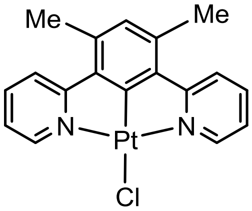

Figure S21. Left: Time-resolved photoluminescence decay of **[Pt(Me2dpb)Cl]** in deaerated DCM at 298 K, including the residuals (λ_{exc} = 376.7 nm, λ_{em} = 490 nm). **Right:** Fitting parameters including pre-exponential factors and confidence limits.

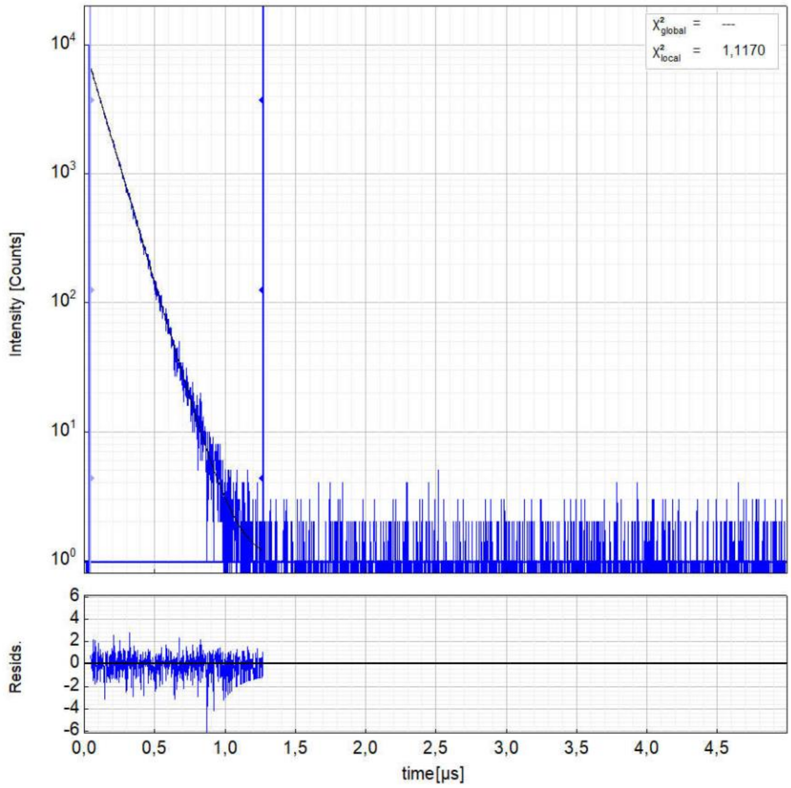

Parameter	Value	Error
A_1[kCnts/Chnl]	6,604	± 0,031
τ_1[ns]	118,50	± 0,30
I_1[kCnts]	391,3	± 1,3
$A_{Rel\,1}$[%]	100,0000	± 0,0000
$I_{Rel\,1}$[%]	100,0000	± 0,0000
$Bkgr_{Dec}$[kCnts]	0,0010	± 0,0003
$\tau_{Av\,Int}$[ns]	118,50	± 0,30
$\tau_{Av\,Amp}$[ns]	118,50	± 0,30

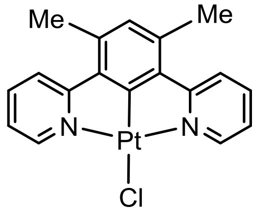

Figure S22. Left: Time-resolved photoluminescence decay of **[Pt(Me$_2$dpb)Cl]** in air-equilibrated 2-MeTHF at 298 K, including the residuals (λ_{exc} = 376.7 nm, λ_{em} = 490 nm). **Right:** Fitting parameters including pre-exponential factors and confidence limits.

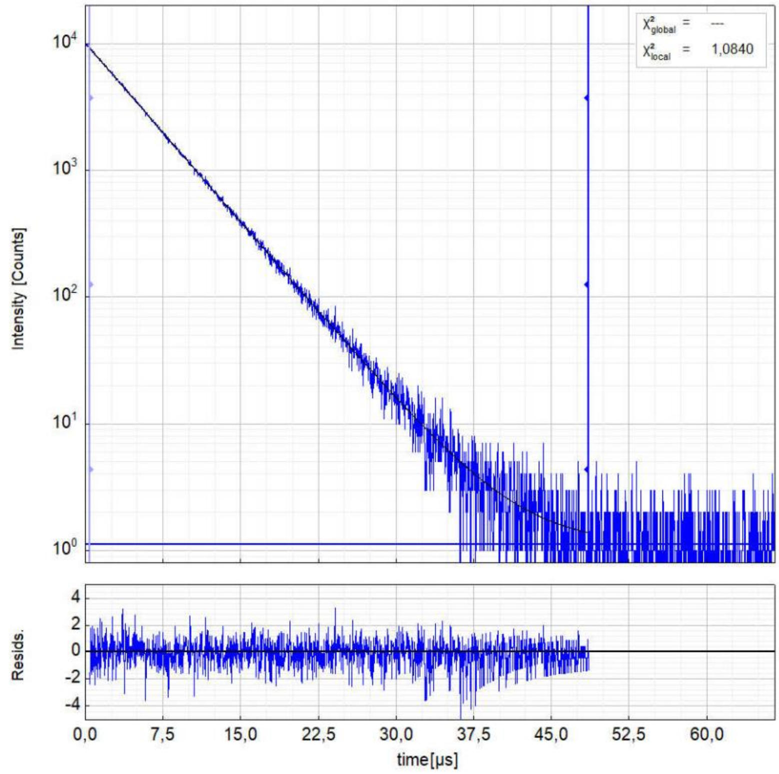

Parameter	Value	Error
A_1[kCnts/Chnl]	9,153	± 0,020
τ_1[ns]	4608,4	± 4,8
I_1[kCnts]	1318,1	± 2,2
$A_{Rel\,1}$[%]	100,0000	± 0,0000
$I_{Rel\,1}$[%]	100,0000	± 0,0000
$Bkgr_{Dec}$[kCnts]	0,0011	± 0,0002
$\tau_{Av\,Int}$[ns]	4608,4	± 4,8
$\tau_{Av\,Amp}$[ns]	4608,4	± 4,8

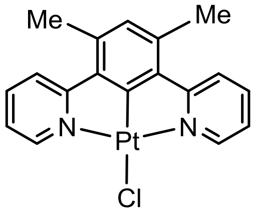

Figure S23. Left: Time-resolved photoluminescence decay of **[Pt(Me$_2$dpb)Cl]** in deaerated 2-MeTHF at 298 K, including the residuals (λ_{exc} = 376.7 nm, λ_{em} = 490 nm). **Right:** Fitting parameters including pre-exponential factors and confidence limits.

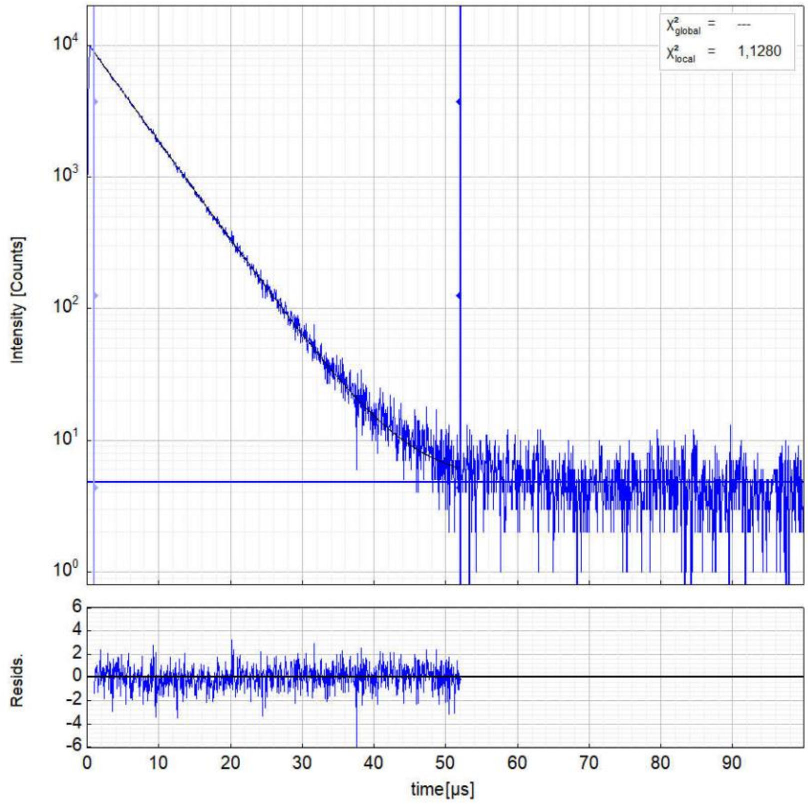

Parameter	Value	Error
A_1[kCnts/Chnl]	8,767	± 0,024
τ_1[ns]	5794,8	± 8,6
I_1[kCnts]	793,8	± 1,3
$A_{Rel\,1}$[%]	100,0000	± 0,0000
$I_{Rel\,1}$[%]	100,0000	± 0,0000
$Bkgr_{Dec}$[kCnts]	0,0048	
$\tau_{Av\,Int}$[ns]	5794,8	± 8,6
$\tau_{Av\,Amp}$[ns]	5794,8	± 8,6

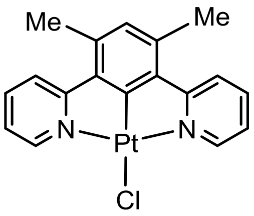

Figure S24. Left: Time-resolved photoluminescence decay of **[Pt(Me$_2$dpb)Cl]** in a frozen glassy matrix of 2-MeTHF at 77 K, including the residuals (λ_{exc} = 376.7 nm, λ_{em} = 485 nm). **Right:** Fitting parameters including pre-exponential factors and confidence limits.

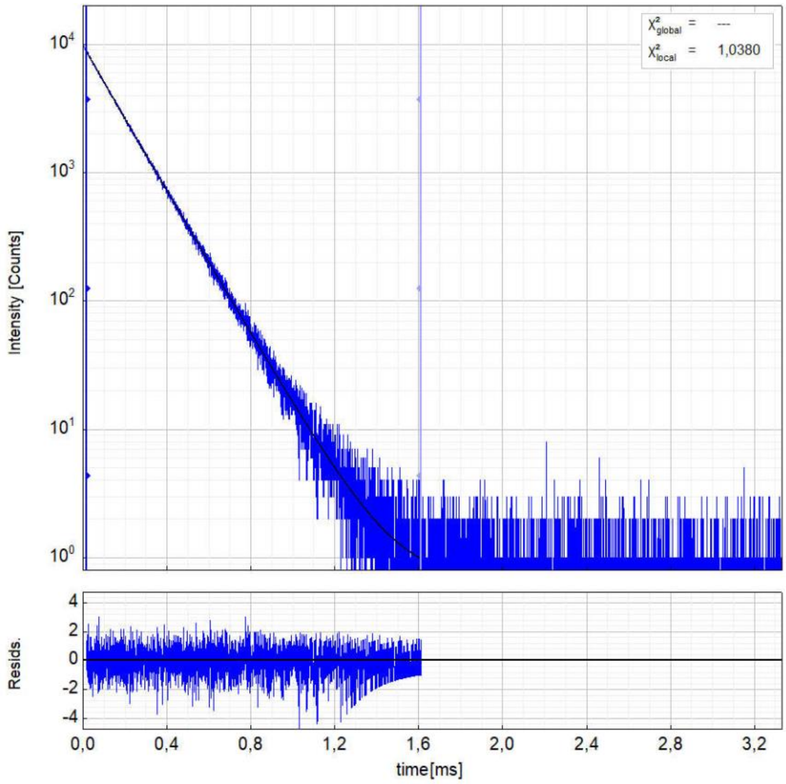

Parameter	Value	Error
A_1[kCnts/Chnl]	8,06	± 0,46
τ_1[µs]	157,4	± 1,6
I_1[kCnts]	2478	± 117
$A_{Rel\,1}$[%]	91,7	± 5,2
$I_{Rel\,1}$[%]	94,4	± 4,5
A_2[kCnts/Chnl]	0,73	± 0,46
τ_2[µs]	102	± 12
I_2[kCnts]	146	± 116
$A_{Rel\,2}$[%]	8,3	± 5,2
$I_{Rel\,2}$[%]	5,6	± 4,5
$Bkgr_{Dec}$[kCnts]	0,0007	± 0,0002
$\tau_{Av\,Int}$[µs]	154,32	± 0,14
$\tau_{Av\,Amp}$[µs]	152,79	± 0,30

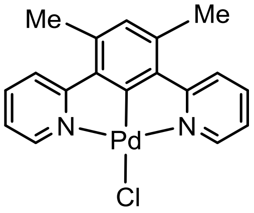

Figure S25. Left: Time-resolved photoluminescence decay of **[Pd(Me$_2$dpb)Cl]** in a frozen glassy matrix of 2-MeTHF at 77 K, including the residuals (λ_{exc} = 376.7 nm, λ_{em} = 470 nm). **Right:** Fitting parameters including pre-exponential factors and confidence limits.

Table S1. Crystal data and structure refinement for the [M(Me$_2$dpb)Cl]·compounds.[a]

Empirical formula	$C_{18}H_{15}ClN_2Ni{\cdot}CH_2Cl_2$	$C_{18}H_{15}ClN_2Pd$	$C_{18}H_{15}ClN_2Pt{\cdot}CH_2Cl_2$ [b]
Formula weight	438.40	401.17	574.78
Crystal system	orthorhombic	monoclinic	triclinic
Space group	*P*bca	*P*2$_1$/c	*P*-1
a/Å	20.9923(9)	7.9526(4)	6.7959(3)
b/Å	7.9234(3)	10.3875(6)	16.0696(7)
c/Å	21.4048(9)	18.2198(10)	16.7798(8)
α/°	90	90	89.408(4)
β/°	90	101.889(2)	87.749(4)
γ/°	90	90	87.427(4)
Volume/Å^3	3560.3(3)	1472.81(14)	1829.15(14)
Z	8	4	4
ρ_{calc} g/cm^3	1.636	1.809	2.096
μ/mm^{-1}	1.544	1.438	8.146
F(000)	1792.0	800.0	1096.0
Crystal size/mm^3	0.07 × 0.07 × 0.01	0.409 × 0.194 × 0.164	0.045 × 0.042 × 0.034
2Θ range for data collection/°	3.806 to 78.912	5.234 to 84.208	5.62 to 55.58
Index ranges	$-37 \le h \le 37$, $-8 \le k \le 14$, $-38 \le l \le 38$	$-15 \le h \le 14$, $-19 \le k \le 19$, $-34 \le l \le 34$	$-8 \le h \le 8$, $-20 \le k \le 21$, $0 \le l \le 21$
Reflections collected	163303	115130	7929
Independent reflections	10641 [R_{int} = 0.2423, R_{sigma} = 0.1213]	10323 [R_{int} = 0.0216, R_{sigma} = 0.0108]	7929 [R_{int} = 0.0450, R_{sigma} = 0.0310]
Data/restraints/parameters	10641/0/229	10323/0/202	7929/0/456
Goodness-of-fit on F^2	1.039	1.109	1.041
Final R indexes [I>=2σ (I)]	R_1 = 0.0876, wR_2 = 0.1316	R_1 = 0.0142, wR_2 = 0.0395	R_1 = 0.0227, wR_2 = 0.0515
Final R indexes [all data]	R_1 = 0.1794, wR_2 = 0.1616	R_1 = 0.0145, wR_2 = 0.0397	R_1 = 0.0264, wR_2 = 0.0527
Largest diff. peak/hole / e Å^{-3}	1.19/–1.30	0.65/–0.72	1.18/–0.93
CCDC	2093819	2095954	1448097

[a] From X-ray diffraction on single crystals. Measured using MoK*α* (*λ* = 0.71073) radiation at 100.0(2) K. [b] Data from Ref. [2].

Table S2. Selected DFT-optimised geometrical data of the complex [Ni(Me$_2$dpb)Cl] compared with experimental data from the crystal structure of [Ni(Me$_2$dpb)Cl]·CH_2Cl_2.[a]

distances / Å	calc.	exp.	angles / °	calc.	exp.
Ni–N	1.921 / 1.922	1.920(2) / 1.921(2)	N–Ni–N	165.5	165.5(1)
Ni–C1	1.830	1.830(3)	N–Ni–C1	82.7	82.9(1) / 82.7(1)
Ni–Cl	2.258	2.2457(8)	C1–Ni–Cl	180.0	179.6(1)
N1–C15 / N2–C25	1.383	1.368(4) / 1.373(4)	dihedral py/ph	0.1/0.2	0.8(1) / 2.7(1)
C2–C15 / C6–C25	1.460	1.467(4) / 1.473(4)	Σ around Ni[b]	359.9	360.0(1)

[a] From DFT (BP86/def2-TZVP) calculated geometries. [b] Sum of angles around the metal.

Table S3. Selected DFT-optimised geometrical data of the complex [Pd(Me$_2$dpb)Cl] compared with experimental data from the crystal structure of [Pd(Me$_2$dpb)Cl].[a]

distances / Å	calc.	exp.	angles / °	calc.	exp.
Pd–N	2.037 / 2.038	2.0348(7) / 2.0355(6)	N–Pd–N	162.0	161.304(25)
Pd–C1	1.919	1.9157(6)	N–Pd–C1	81.0	80.724(23) / 80.591(24)
Pd–Cl	2.427	2.4512(4)	C1–Pd–Cl	180.0	178.271(21)
N1–C15 / N2–C25	1.382	1.3710(8) / 1.3711(7)	dihedral py/ph	0.1 / 0.2	1.046(28) / 2.720(34)
C2–C15 / C6–C25	1.468	1.4712(8) / 1.4726(8)	Σ around Pd [b]	360.0	360.01

[a] From DFT (BP86/def2-TZVP) calculated geometries. [b] Sum of angles around the metal.

Table S4. Selected DFT-optimised geometrical data of the complex [Pt(Me_2dpb)Cl] compared with experimental data from the crystal structure of [Pt(Me_2dpb)Cl]·CH_2Cl_2.[a]

distances / Å	calc.	exp. [b]	angles / °	calc.	exp.
Pt–N	2.029	2.026(4) / 2.022(4)<br>2.018(3) / 2.025(3)	N–Pt–N	162.7	162.3(1) / 162.5(1)
Pt–C1	1.922	1.915(4) / 1.917(5)	N–Pt–C1	81.3	80.9(2) / 81.4(2)<br>81.3(2) / 81.2(2)
Pt–Cl	2.439	2.419(1) / 2.420(1)	C1–Pt–Cl	179.9	178.7(1) / 179.0(1)
N1–C15 / N2–C25	1.389	1.382(6) / 1.369(6)<br>1.377(5) / 1.383(6)	dihedral py/ph	0.1	1.3(1) / 2.5(1)<br>2.6(1) / 3.8(2)
C2–C15 / C6–C25	1.468	1.475(7) / 1.475(7)<br>1.473(7) / 1.482(7)	Σ around Pt [c]	360.0	360.0(1) / 360.0(1)

[a] From DFT (BP86/def2-TZVP) calculated geometries. [b] Data from Ref. [2], two independent molecules in the unit cell. [c] Sum of angles around the metal.

Table S5. Selected absorption maxima for Me_2dpbH and the complexes [M(Me_2dpb)Cl] (M = Ni, Pd, Pt)[a].

λ / nm (ε / L·mol^{-1}·cm^{-1}) [b]	Me_2dpbH	[Ni(Me_2dpb)Cl]	[Pd(Me_2dpb)Cl]	[Pt(Me_2dpb)Cl]
λ_1	230 (26600)	230 (36300)	217 (27300)	230 (37200)
λ_2	262 (17400)	274 (31000)	239 (25400)	259 (27800)
λ_3	-	307 (11400)	266 (19600)	289 (21000)
λ_4	-	333 (8600)	329 (6300)	333 (6900)
λ_5	-	406 (6200)	344 (5800)	364 (6200)
λ_6	-	430 (5800)	364 (6800)	382 (8600)

[a] Measured in THF at 298 K. [b] Absorption maxima λ_1 / nm, extinction coefficient ε / L·mol^{-1}·cm^{-1}.

Table S6. Selected TD-DFT calculated vertical $S_0 \rightarrow S_n$ transitions for [Pt(Me_2dpb)Cl].[a]

n	λ /nm	f_{osc}	participating MOs (contribution)
1	435.4	0.00199	HOMO→LUMO (95%)
2	420.5	0.08224	HOMO→L+1 (92%)
8	349.0	0.03999	H-4→L+1 (21%), H-1→L+1 (61%), HOMO→L+3 (10%)
10	316.9	0.18427	H-4→LUMO (75%), H-1→L+4 (13%)
11	324.8	0.01311	H-1→L+2 (11%), HOMO→L+4 (79%)
12	307.9	0.05936	H-4→L+1 (34%), H-1→L+1 (16%), H-1→L+2 (35%)
13	307.4	0.08646	H-5→LUMO (84%)
20	275.4	0.04416	H-4→L+1 (32%), H-1→L+2 (46%)
21	275.2	0.56954	H-5→L+1 (16%), H-4→LUMO (13%), H-1→L+5 (48%)
22	288.1	0.01842	H-6→L+1 (96%)
28	247.7	0.01056	H-7→LUMO (55%), H-4→L+2 (20%)
30	244.7	0.24251	H-7→L+1 (45%), H-5→L+2 (24%)
33	237.4	0.21964	H-3→L+5 (81%)
35	236.0	0.01578	H-9→L+1 (41%), H-1→L+4 (45%)
36	234.5	0.05677	H-9→LUMO (22%), H-2→L+5 (51%), HOMO→L+4 (11%)
38	230.7	0.43147	H-2→L+5 (12%), HOMO→L+4 (52%)
39	229.9	0.02279	H-9→L+1 (40%), HOMO→L+6 (48%)
41	223.7	0.06388	H-10→LUMO (54%), HOMO→L+6 (18%)

[a] For oscillator strength f > 0.01 except for the lowest-energy transition.

Table S7. Selected TD-DFT calculated vertical $S_0 \rightarrow S_n$ transitions for [Pd(Me_2dpb)Cl].[a]

n	λ /nm	f_{osc}	participating MOs (contribution)
1	403.1	0.00155	HOMO→LUMO (94%)
2	393.7	0.06978	HOMO→L+1 (88%)

5	367.9	0.01557	H-1→LUMO (89%)
8	330.5	0.05802	H-4→L+1 (25%), H-1→L+1 (56%)
10	312.7	0.12513	H-5→L+1 (16%), H-4→LUMO (68%)
12	303.8	0.03080	H-4→L+1 (39%), H-1→L+2 (42%)
15	306.5	0.07723	H-5→LUMO (12%), HOMO→L+3 (69%)
16	304.2	0.06202	H-5→L+1 (71%), H-1→L+3 (12%)
22	280.8	0.04850	H-2→L+4 (71%)
24	273.6	0.03061	H-4→L+1 (24%), H-1→L+2 (50%)
26	269.9	0.45647	H-5→L+2 (11%), H-1→L+3 (51%)
30	262.6	0.04535	H-7→L+1 (12%), H-5→L+2 (60%), H-4→L+3 (16%)
32	248.98	0.01860	H-7→L+1 (55%), H-4→L+2 (20%)
33	249.7	0.01615	H-5→L+2 (11%), H-4→L+3 (72%)
34	246.6	0.34534	H-7→L+1 (66%)
42	231.7	0.15431	H-6→L+4 (10%), HOMO→L+5 (62%)
43	224.4	0.03017	H-10→LUMO (23%), H-9→L+1 (15%), H-1→L+5 (36%), HOMO→L+6 (16%)
45	221.3	0.01453	H-10→LUMO (47%), HOMO→L+6 (39%)

[a] For oscillator strength f > 0.01 except for the lowest-energy transition.

Table S8. Selected TD-DFT calculated vertical $S_0 \rightarrow S_n$ transitions for [Ni(Me_2dpb)Cl].[a]

n	λ /nm	f_{osc}	participating MOs (contribution)
1	512.7	0.00315	H-1→LUMO (96%)
5	429.5	0.06825	H-2→LUMO (52%), HOMO→L+1 (43%)
10	390.0	0.01432	H-2→L+1 (62%), H-1→L+3 (16%)
15	342.8	0.13637	H-5→L+1 (10%), H-3→LUMO (75%)
18	319.8	0.01868	H-5→LUMO (35%), H-3→L+1 (15%), H-2→L+2 (13%), HOMO→L+3 (25%)
19	316.3	0.05371	H-5→LUMO (55%), H-2→L+2 (11%), HOMO→L+4 (12%)
22	292.4	0.13588	H-3→L+1 (48%), H-2→L+2 (25%)
25	294.0	0.35489	H-5→L+1 (38%), H-2→L+4 (37%)
36	255.1	0.22739	H-7→L+1 (11%), H-5→L+2 (22%), H-3→L+4 (27%), H-2→L+5 (24%)
38	247.5	0.13795	H-9→L+1 (10%), H-8→L+3 (26%), H-2→L+5 (38%)
39	245.3	0.01394	H-5→L+4 (67%), HOMO→L+5 (14%)
40	251.0	0.09256	H-8→L+3 (59%), H-2→L+5 (15%)
41	241.2	0.11351	H-9→LUMO (47%), HOMO→L+5 (24%)
48	237.4	0.05961	H-7→L+2 (80%)
50	224.3	0.01363	H-10→LUMO (52%), H-3→L+5 (29%)
61	220.0	0.26143	H-12→L+1 (92%), H-7→L+4 (44%), H-6→L+3 (24%)

[a] For oscillator strength f > 0.01 except for the lowest-energy transition.

Table S9. Selected UV-vis absorption maxima of the reduced and oxidised complexes [M(Me_2dpb)Cl]^n [a]

		λ_1 (ε)	λ_2 (ε)	λ_3 (ε)	λ_4 (ε)	λ_5 (ε)	λ_6 (ε)
[Pt(Me_2dpb)Cl]	n = –1	239	276	312	390	415	582
	n = –2	238	-	316	-	418	591
	n = +1	245	281	323			
[Pd(Me_2dpb)Cl]	n = –1	246	277	319	370	-	570
	n = –2	246	274	-	381	-	582
	n = +1	241	271	310			
[NiMe_2dpb)Cl]	n = –1	230	275	330	-	429	644
	n = –2	230	248	-	393	-	644
	n = +1	222	252	301			

[a] Generated through electrolysis in THF/*n*-Bu_4NPF_6.

Table S10. Fractional atomic coordinates (×10[4]) and equivalent isotropic displacement parameters (Å^2×10^3) for [Ni(Me$_2$dpb)Cl]·CH_2Cl_2.U_{eq} is defined as 1/3 of of the trace of the orthogonalised U_{IJ} tensor.

Atom	*x*	*y*	*z*	U(eq)
Ni1	5875.0(2)	3109.9(5)	4083.4(2)	11.61(8)
Cl1	6354.6(4)	3167.8(11)	3145.8(4)	21.88(15)
Cl32	7550.9(4)	2086.5(12)	1777.4(4)	26.08(18)
Cl31	8300.5(5)	3663.4(14)	2764.8(5)	34.8(2)
N2	5137.8(11)	1848(3)	3830.7(11)	14.1(4)
N1	6517.9(11)	4313(3)	4540.8(12)	13.1(4)
C1	5484.8(12)	3046(4)	4847.5(12)	11.0(4)
C2	5785.3(13)	3812(3)	5355.5(13)	12.2(5)
C6	4891.3(13)	2256(3)	4893.3(14)	12.6(5)
C15	6401.7(14)	4533(4)	5164.4(14)	13.8(5)
C4	4883.4(14)	3049(4)	5972.3(14)	16.5(5)
C5	4579.5(14)	2262(4)	5474.8(14)	14.2(5)
C21	5030.7(16)	1273(4)	3252.3(15)	19.4(6)
C25	4694.4(14)	1551(3)	4289.5(14)	13.6(5)
C3	5481.2(14)	3834(4)	5940.6(14)	15.5(5)
C11	7061.3(14)	4913(4)	4290.6(16)	17.5(6)
C24	4137.9(15)	672(4)	4148.8(16)	18.2(5)
C14	6852.1(15)	5352(4)	5534.4(16)	19.2(6)
C22	4485.4(16)	389(4)	3090.8(16)	22.0(6)
C8	3940.3(14)	1453(4)	5584.6(15)	18.2(6)
C13	7411.4(15)	5957(4)	5271.8(17)	21.2(6)
C23	4031.9(15)	84(4)	3546.8(17)	21.2(6)
C7	5749.9(16)	4598(4)	6522.5(15)	20.6(6)
C12	7519.3(16)	5747(4)	4640.0(17)	21.5(6)
C31	7833.2(17)	1899(5)	2553.3(15)	22.5(6)

Table S11. Anisotropic displacement parameters (Å^2×10^3) for [Ni(Me$_2$dpb)Cl]·CH_2Cl_2. The Anisotropic displacement factor exponent takes the form: $-2\pi^2[h^2a^{*2}U_{11}+2hka^*b^*U_{12}+\ldots]$.

Atom	U_{11}	U_{22}	U_{33}	U_{23}	U_{13}	U_{12}
Ni1	10.51(14)	12.21(14)	12.10(15)	1.40(14)	1.24(13)	-0.50(14)
Cl1	19.7(3)	29.0(4)	17.0(3)	2.0(3)	5.2(3)	-3.1(3)
Cl32	26.8(4)	29.4(4)	22.1(4)	3.1(3)	-3.3(3)	-1.9(3)
Cl31	39.0(5)	39.1(5)	26.4(4)	2.9(4)	-10.6(4)	-8.6(4)
N2	13.1(10)	14.5(10)	14.6(10)	0.7(10)	-0.9(8)	1.0(9)
N1	8.9(9)	11.4(10)	18.9(11)	0.9(9)	1.1(8)	-0.2(8)
C1	10.6(10)	11.2(10)	11.2(10)	1.5(10)	1.0(8)	0.8(9)
C2	12.4(12)	10.9(10)	13.4(12)	2.2(9)	0.2(9)	1.9(9)
C6	10.3(11)	11.2(11)	16.2(13)	2.6(10)	-0.7(9)	-0.7(9)
C15	13.2(11)	10.9(11)	17.4(13)	1.7(10)	-1.2(10)	0.7(9)
C4	17.9(12)	18.1(12)	13.5(12)	3.9(12)	4.5(10)	6.6(11)
C5	13.6(11)	11.5(11)	17.5(13)	4.0(10)	3.3(10)	3.9(9)
C21	22.1(15)	22.6(14)	13.5(13)	-0.3(12)	-0.5(11)	-1.6(12)
C25	12.6(11)	11.1(11)	17.3(12)	2.4(10)	-1.6(10)	1.0(9)
C3	19.0(13)	13.7(11)	13.6(12)	2.5(11)	-0.7(11)	5.2(10)
C11	11.0(11)	14.1(12)	27.3(16)	2.0(12)	1.6(11)	0.0(10)
C24	13.5(11)	16.3(12)	25.0(15)	3.5(12)	-0.9(12)	-2.4(11)
C14	18.4(14)	15.4(12)	23.9(15)	0.3(12)	-6.7(12)	-0.8(11)
C22	24.5(15)	21.9(14)	19.8(15)	-2.8(13)	-7.7(12)	-0.9(13)
C8	14.1(12)	19.0(13)	21.5(14)	4.5(12)	4.9(11)	0.3(11)
C13	15.1(13)	15.6(13)	32.9(18)	-1.3(13)	-6.6(12)	-1.8(11)
C23	16.4(14)	17.6(13)	29.6(17)	0.0(12)	-8.5(12)	-1.9(11)

C7	25.3(16)	22.5(15)	13.9(13)	-1.3(12)	-1.0(11)	3.4(12)
C12	11.6(12)	13.5(12)	39.4(19)	-0.2(13)	2.4(13)	0.4(10)
C31	24.7(15)	25.7(15)	17.3(13)	6.0(14)	2.1(11)	4.8(14)

Table S12. Selected bond lengths for $[Ni(Me_2dpb)Cl]\cdot CH_2Cl_2$.

Atom	Atom	Length/Å	Atom	Atom	Length/Å
Ni1	Cl1	2.2457(8)	C6	C5	1.406(4)
Ni1	N2	1.920(2)	C6	C25	1.467(4)
Ni1	N1	1.921(2)	C15	C14	1.394(4)
Ni1	C1	1.830(3)	C4	C5	1.389(4)
Cl32	C31	1.769(3)	C4	C3	1.402(4)
Cl31	C31	1.767(4)	C5	C8	1.506(4)
N2	C21	1.338(4)	C21	C22	1.386(5)
N2	C25	1.373(4)	C25	C24	1.393(4)
N1	C15	1.368(4)	C3	C7	1.495(4)
N1	C11	1.347(4)	C11	C12	1.386(5)
C1	C2	1.396(4)	C24	C23	1.388(5)
C1	C6	1.398(4)	C14	C13	1.387(5)
C2	C15	1.473(4)	C22	C23	1.385(5)
C2	C3	1.406(4)	C13	C12	1.381(5)

Table S13. Selected bond angles for $[Ni(Me_2dpb)Cl]\cdot CH_2Cl_2$.

Atom	Atom	Atom	Angle/°	Atom	Atom	Atom	Angle/°
N2	Ni1	Cl1	96.91(8)	N1	C15	C14	119.5(3)
N2	Ni1	N1	165.54(11)	C14	C15	C2	128.3(3)
N1	Ni1	Cl1	97.49(8)	C5	C4	C3	125.0(3)
C1	Ni1	Cl1	179.58(10)	C6	C5	C8	123.5(3)
C1	Ni1	N2	82.91(11)	C4	C5	C6	117.8(3)
C1	Ni1	N1	82.68(11)	C4	C5	C8	118.7(3)
C21	N2	Ni1	125.0(2)	N2	C21	C22	122.8(3)
C21	N2	C25	119.3(3)	N2	C25	C6	111.9(2)
C25	N2	Ni1	115.7(2)	N2	C25	C24	120.0(3)
C15	N1	Ni1	115.81(19)	C24	C25	C6	128.1(3)
C11	N1	Ni1	124.6(2)	C2	C3	C7	125.2(3)
C11	N1	C15	119.6(3)	C4	C3	C2	116.3(3)
C2	C1	Ni1	118.8(2)	C4	C3	C7	118.5(3)
C2	C1	C6	122.9(3)	N1	C11	C12	122.8(3)
C6	C1	Ni1	118.3(2)	C23	C24	C25	120.2(3)
C1	C2	C15	110.5(2)	C13	C14	C15	120.3(3)
C1	C2	C3	119.6(3)	C23	C22	C21	118.7(3)
C3	C2	C15	129.9(3)	C12	C13	C14	119.6(3)
C1	C6	C5	118.4(3)	C22	C23	C24	119.1(3)
C1	C6	C25	111.1(3)	C13	C12	C11	118.2(3)
C5	C6	C25	130.5(3)	Cl31	C31	Cl32	111.10(19)
N1	C15	C2	112.2(2)				

Table S14. Selected torsion angles for $[Ni(Me_2dpb)Cl]\cdot CH_2Cl_2$.

A	B	C	D	Angle/°	A	B	C	D	Angle/°
Ni1	N2	C21	C22	-179.0(2)	C2	C1	C6	C25	-179.5(3)
Ni1	N2	C25	C6	-1.1(3)	C2	C15	C14	C13	-180.0(3)
Ni1	N2	C25	C24	178.9(2)	C6	C1	C2	C15	-180.0(3)
Ni1	N1	C15	C2	-1.2(3)	C6	C1	C2	C3	-1.3(4)
Ni1	N1	C15	C14	178.2(2)	C6	C25	C24	C23	-179.6(3)
Ni1	N1	C11	C12	-178.7(2)	C15	N1	C11	C12	0.5(4)
Ni1	C1	C2	C15	-1.2(3)	C15	C2	C3	C4	179.0(3)
Ni1	C1	C2	C3	177.5(2)	C15	C2	C3	C7	-2.0(5)

Ni1	C1	C6	C5	-177.6(2)	C15	C14	C13	C12	0.2(5)
Ni1	C1	C6	C25	1.7(3)	C5	C6	C25	N2	178.9(3)
N2	Ni1	C1	C2	179.4(2)	C5	C6	C25	C24	-1.0(5)
N2	Ni1	C1	C6	-1.8(2)	C5	C4	C3	C2	0.2(4)
N2	C21	C22	C23	-0.1(5)	C5	C4	C3	C7	-178.9(3)
N2	C25	C24	C23	0.5(4)	C21	N2	C25	C6	179.5(3)
N1	Ni1	C1	C2	0.5(2)	C21	N2	C25	C24	-0.6(4)
N1	Ni1	C1	C6	179.3(2)	C21	C22	C23	C24	0.0(5)
N1	C15	C14	C13	0.7(4)	C25	N2	C21	C22	0.4(5)
N1	C11	C12	C13	0.4(5)	C25	C6	C5	C4	-179.5(3)
C1	C2	C15	N1	1.5(3)	C25	C6	C5	C8	1.0(5)
C1	C2	C15	C14	-177.9(3)	C25	C24	C23	C22	-0.2(5)
C1	C2	C3	C4	0.6(4)	C3	C2	C15	N1	-177.0(3)
C1	C2	C3	C7	179.6(3)	C3	C2	C15	C14	3.6(5)
C1	C6	C5	C4	-0.3(4)	C3	C4	C5	C6	-0.3(4)
C1	C6	C5	C8	-179.8(3)	C3	C4	C5	C8	179.2(3)
C1	C6	C25	N2	-0.4(3)	C11	N1	C15	C2	179.5(3)
C1	C6	C25	C24	179.7(3)	C11	N1	C15	C14	-1.0(4)
C2	C1	C6	C5	1.2(4)	C14	C13	C12	C11	-0.7(5)

Table S15. Hydrogen atom coordinates (Å×10^4) and isotropic displacement parameters (Å^2×10^3) for [Ni(Me$_2$dpb)Cl]·CH_2Cl_2.

Atom	*x*	*y*	*z*	U(eq)
H4	4670.11	3055.22	6363.73	20
H21	5341.8	1479.13	2939.11	23
H11	7133.63	4757.9	3856.28	21
H24	3829.96	474.53	4465.77	22
H14	6775.67	5497.28	5968.53	23
H22	4423.89	-0.91	2675.37	26
H8A	3626.23	1942.32	5298.47	27
H8B	3806.72	1653.02	6016.93	27
H8C	3972.05	235.21	5509.73	27
H13	7718.42	6511.96	5524.95	25
H23	3653.58	-518.31	3449.09	25
H7A	6125.45	3956.17	6654.54	31
H7B	5428.04	4568.49	6854.01	31
H7C	5872.14	5771.23	6440.42	31
H12	7896.98	6163.74	4450.19	26
H31A	7466.76	1802.39	2842.14	27
H31B	8091.84	859.27	2592.12	2

Table S16. Fractional atomic coordinates (×10^4) and equivalent isotropic displacement parameters (Å^2×10^3) for [Pd(Me$_2$dpb)Cl].U_{eq} is defined as 1/3 of of the trace of the orthogonalised U_{IJ} tensor.

Atom	*x*	*y*	*z*	U(eq)
Pd1	2441.7(2)	3514.7(2)	4405.2(2)	10.34(1)
Cl1	2245.5(2)	1608.2(2)	3599.2(2)	18.51(3)
N1	1655.1(6)	2738.7(5)	5303.3(3)	12.37(6)
N2	3288.0(6)	4806.9(5)	3723.2(3)	13.24(7)
C1	2534.0(6)	5024.9(5)	5015.7(3)	11.28(7)
C25	3515.1(7)	6037.3(5)	3998.2(3)	13.64(7)
C2	2029.4(6)	4911.0(5)	5706.8(3)	11.96(7)
C15	1539.9(7)	3590.1(5)	5865.8(3)	12.30(7)
C6	3078.0(7)	6181.0(5)	4741.0(3)	12.63(7)
C11	1297.9(9)	1487.1(6)	5371.2(4)	16.52(9)
C12	792.3(9)	994.7(7)	6000.4(4)	19.5(1)
C8	3747.5(10)	8595.2(6)	4988.5(5)	21.53(11)

C14	1036.1(8)	3137.3(7)	6511.1(3)	17.41(9)
C3	2043.9(7)	6030.0(6)	6146.9(3)	14.42(8)
C5	3137.9(7)	7295.8(5)	5189.6(3)	15.20(8)
C21	3630.9(8)	4511.1(7)	3053.4(3)	17.55(9)
C13	655.0(9)	1842.5(7)	6573.6(4)	19.97(10)
C24	4095.2(9)	6984.8(7)	3564.9(4)	19.71(10)
C22	4227.1(9)	5416.4(8)	2607.8(4)	21.77(11)
C23	4455.5(9)	6666.7(8)	2872.3(4)	22.88(12)
C7	1477.1(8)	6073.8(7)	6886.3(4)	19.39(10)
C4	2604.4(8)	7174.8(6)	5872.1(4)	16.49(8)

Table S17. Anisotropic displacement parameters ($Å^2 \times 10^3$) for [Pd(Me_2dpb)Cl]. The anisotropic displacement factor exponent takes the form: $-2\pi^2[h^2a^{*2}U_{11}+2hka^*b^*U_{12}+\ldots]$.

Atom	U_{11}	U_{22}	U_{33}	U_{23}	U_{13}	U_{12}
Pd1	11.86(2)	10.50(2)	8.90(2)	0.16(1)	2.73(1)	-0.22(1)
Cl1	27.98(7)	14.36(5)	13.48(5)	-2.77(4)	4.96(5)	-0.53(4)
N1	13.79(15)	12.48(15)	10.93(14)	0.68(12)	2.74(12)	-1.97(12)
N2	13.10(15)	15.63(17)	11.69(15)	2.39(13)	4.19(12)	0.82(13)
C1	11.29(15)	11.23(16)	11.32(16)	-0.07(13)	2.36(12)	-0.50(12)
C25	11.67(16)	14.57(18)	14.59(18)	3.95(15)	2.52(14)	-0.51(14)
C2	11.52(16)	13.50(17)	10.90(16)	-1.08(13)	2.46(13)	-0.18(13)
C15	11.67(16)	15.01(18)	10.43(16)	0.55(13)	2.77(13)	-0.84(13)
C6	11.92(16)	11.53(17)	14.11(18)	1.23(14)	1.90(13)	-0.53(13)
C11	20.2(2)	13.8(2)	15.4(2)	1.78(15)	3.18(17)	-3.87(16)
C12	21.6(2)	18.0(2)	18.8(2)	5.61(18)	3.87(18)	-4.82(18)
C8	21.9(3)	11.8(2)	29.2(3)	1.57(19)	1.4(2)	-1.71(17)
C14	19.0(2)	21.9(2)	12.78(18)	2.03(17)	6.55(16)	-0.99(18)
C3	13.15(17)	16.4(2)	13.25(18)	-3.45(15)	1.67(14)	1.29(15)
C5	13.99(18)	11.19(17)	19.0(2)	-0.05(15)	0.22(15)	-0.12(14)
C21	18.2(2)	22.5(2)	13.39(19)	3.12(17)	6.72(16)	2.78(18)
C13	20.4(2)	24.3(3)	16.4(2)	6.61(19)	6.39(18)	-2.7(2)
C24	19.2(2)	19.5(2)	20.6(2)	7.64(19)	4.52(18)	-3.40(18)
C22	20.2(2)	31.0(3)	15.9(2)	7.9(2)	7.95(18)	2.8(2)
C23	19.8(2)	29.0(3)	20.6(3)	11.9(2)	6.1(2)	-2.2(2)
C7	18.2(2)	25.3(3)	15.1(2)	-6.53(19)	4.43(17)	1.30(19)
C4	16.9(2)	13.27(19)	18.1(2)	-3.90(16)	0.86(16)	1.10(15)

Table S18. Selected bond lengths for [Pd(Me_2dpb)Cl].

Atom	Atom	Length/Å	Atom	Atom	Length/Å
Pd1	Cl1	2.45121(18)	C2	C3	1.4108(8)
Pd1	N1	2.0349(5)	C15	C14	1.3993(8)
Pd1	N2	2.0354(5)	C6	C5	1.4126(8)
Pd1	C1	1.9158(5)	C11	C12	1.3884(9)
N1	C15	1.3709(7)	C12	C13	1.3875(11)
N1	C11	1.3419(7)	C8	C5	1.5048(9)
N2	C25	1.3711(8)	C14	C13	1.3885(10)
N2	C21	1.3398(8)	C3	C7	1.5067(8)
C1	C2	1.4030(7)	C3	C4	1.3984(9)
C1	C6	1.4032(7)	C5	C4	1.3999(9)
C25	C6	1.4725(8)	C21	C22	1.3882(9)
C25	C24	1.3981(8)	C24	C23	1.3903(11)
C2	C15	1.4713(8)	C22	C23	1.3841(12)

Table S19. Selected bond angles for [Pd(Me_2dpb)Cl].

Atom	Atom	Atom	Angle/°	Atom	Atom	Atom	Angle/°

N1	Pd1	Cl1	99.765(15)	N1	C15	C2	113.16(4)
N1	Pd1	N2	161.30(2)	N1	C15	C14	119.08(5)
N2	Pd1	Cl1	98.935(15)	C14	C15	C2	127.74(5)
C1	Pd1	Cl1	178.272(16)	C1	C6	C25	112.93(5)
C1	Pd1	N1	80.72(2)	C1	C6	C5	118.12(5)
C1	Pd1	N2	80.59(2)	C5	C6	C25	128.95(5)
C15	N1	Pd1	115.13(4)	N1	C11	C12	122.40(6)
C11	N1	Pd1	124.32(4)	C13	C12	C11	117.95(6)
C11	N1	C15	120.53(5)	C13	C14	C15	119.98(6)
C25	N2	Pd1	115.27(4)	C2	C3	C7	124.35(6)
C21	N2	Pd1	124.04(4)	C4	C3	C2	117.58(5)
C21	N2	C25	120.69(5)	C4	C3	C7	118.07(5)
C2	C1	Pd1	117.92(4)	C6	C5	C8	124.66(6)
C2	C1	C6	123.95(5)	C4	C5	C6	117.40(5)
C6	C1	Pd1	118.12(4)	C4	C5	C8	117.94(6)
N2	C25	C6	113.08(5)	N2	C21	C22	122.38(7)
N2	C25	C24	118.94(6)	C12	C13	C14	120.05(6)
C24	C25	C6	127.98(6)	C23	C24	C25	119.93(7)
C1	C2	C15	113.05(5)	C23	C22	C21	117.92(6)
C1	C2	C3	118.05(5)	C22	C23	C24	120.15(6)
C3	C2	C15	128.90(5)	C3	C4	C5	124.87(5)

Table S20. Selected torsion angles for [Pd(Me_2dpb)Cl].

A	B	C	D	Angle/°	A	B	C	D	Angle/°
Pd1	N1	C15	C2	-0.48(6)	C25	C24	C23	C22	0.56(10)
Pd1	N1	C15	C14	-178.96(4)	C2	C1	C6	C25	-179.33(5)
Pd1	N1	C11	C12	178.88(5)	C2	C1	C6	C5	0.64(8)
Pd1	N2	C25	C6	-0.56(6)	C2	C15	C14	C13	-178.47(6)
Pd1	N2	C25	C24	179.96(4)	C2	C3	C4	C5	0.60(9)
Pd1	N2	C21	C22	-179.18(5)	C15	N1	C11	C12	0.36(9)
Pd1	C1	C2	C15	1.73(6)	C15	C2	C3	C7	-2.06(9)
Pd1	C1	C2	C3	-178.02(4)	C15	C2	C3	C4	178.69(5)
Pd1	C1	C6	C25	-0.27(6)	C15	C14	C13	C12	0.74(10)
Pd1	C1	C6	C5	179.70(4)	C6	C1	C2	C15	-179.22(5)
N1	C15	C14	C13	-0.24(9)	C6	C1	C2	C3	1.03(8)
N1	C11	C12	C13	0.14(10)	C6	C25	C24	C23	179.65(6)
N2	C25	C6	C1	0.53(7)	C6	C5	C4	C3	1.06(9)
N2	C25	C6	C5	-179.43(5)	C11	N1	C15	C2	178.17(5)
N2	C25	C24	C23	-0.96(9)	C11	N1	C15	C14	-0.30(8)
N2	C21	C22	C23	-0.48(10)	C11	C12	C13	C14	-0.68(10)
C1	C2	C15	N1	-0.73(7)	C8	C5	C4	C3	-178.41(6)
C1	C2	C15	C14	177.58(6)	C3	C2	C15	N1	178.99(5)
C1	C2	C3	C7	177.65(5)	C3	C2	C15	C14	-2.70(9)
C1	C2	C3	C4	-1.61(8)	C21	N2	C25	C6	-179.88(5)
C1	C6	C5	C8	177.80(6)	C21	N2	C25	C24	0.64(8)
C1	C6	C5	C4	-1.63(8)	C21	C22	C23	C24	0.14(10)
C25	N2	C21	C22	0.08(9)	C24	C25	C6	C1	179.96(6)
C25	C6	C5	C8	-2.23(9)	C24	C25	C6	C5	0.00(10)
C25	C6	C5	C4	178.33(5)	C7	C3	C4	C5	-178.70(6)

Table S21. Hydrogen atom coordinates (Å×10^4) and isotropic displacement parameters (Å^2×10^3) for [Pd(Me_2dpb)Cl].

Atom	*x*	*y*	*z*	U(eq)
H11	1394.43	917.22	4974.2	20
H12	547.9	104.62	6037.4	23
H8A	4911.07	8517.75	4892.2	32
H8B	3757.4	9194.82	5404.65	32
H8C	2971.03	8919.45	4537.67	32

H14	954.82	3715.6	6906.24	21
H21	3459.84	3650.64	2877.11	21
H13	299.95	1536.97	7009.19	24
H24	4242.84	7845.08	3743.57	24
H22	4470.75	5185.1	2136.13	26
H23	4859.46	7308.85	2579.88	27
H7A	315.93	5716.3	6823.98	29
H7B	1475.91	6968.1	7057.47	29
H7C	2270.99	5565.49	7258.25	29
H4	2624.71	7926.39	6170.92	20

References

(1) Von der Stück, R.; Schmitz, S.; Klein, A. C–X vs C–H activation for the synthesis of the cyclometalated complexes [Pd(YPhbpy)X] (HPhbpy = 6-phenyl-2,2′-bipyridine; X/Y = (pseudo)halides), *Inorg. Chem. Res.* **2021**, *5*, 173–192.

(2) Schulze, B.; Friebe, C.; Jäger, M.; Görls, H.; Birckner, E.; Winter, A.; Schubert, U.S. Pt^{II} Phosphors with Click-Derived 1,2,3-Triazole-Containing Tridentate Chelates, *Organometallics* **2018**, *37*, 145–155.
